# Benzimidazole-Based Derivatives as Apoptotic Antiproliferative Agents: Design, Synthesis, Docking, and Mechanistic Studies

**DOI:** 10.3390/molecules29020446

**Published:** 2024-01-16

**Authors:** Bahaa G. M. Youssif, Martha M. Morcoss, Stefan Bräse, Mohamed Abdel-Aziz, Hamdy M. Abdel-Rahman, Dalal A. Abou El-Ella, El Shimaa M. N. Abdelhafez

**Affiliations:** 1 Pharmaceutical Organic Chemistry Department, Faculty of Pharmacy, Assiut University, Assiut 71526, Egypt; 2 Department of Pharmaceutical Chemistry, Faculty of Pharmacy, Nahda University, Beni-Suef 62513, Egypt; martha.moheb@nub.edu.eg; 3 Institute of Biological and Chemical Systems, IBCS-FMS, Karlsruhe Institute of Technology, 76131 Karlsruhe, Germany; 4 Department of Medicinal Chemistry, Faculty of Pharmacy, Minia University, Minia 61519, Egypt; abulnil@hotmail.com (M.A.-A.); shimaanaguib_80@mu.edu.eg (E.S.M.N.A.); 5 Department of Medicinal Chemistry, Faculty of Pharmacy, Assiut University, Assiut 71526, Egypt; hamdym@aun.edu.eg; 6 Pharmaceutical Chemistry Department, Faculty of Pharmacy, Badr University in Assiut (BUA), Assiut 71536, Egypt; 7 Department of Pharmaceutical Chemistry, Faculty of Pharmacy Ain Shams University, Cairo 11566, Egypt; dalal@pharma.asu.edu.eg

**Keywords:** benzimidazole, hydrazone, thiosemicarbazide, antiproliferative, NCI

## Abstract

A new class of benzimidazole-based derivatives (**4a**–**j**, **5**, and **6**) with potential dual inhibition of EGFR and BRAF^V600E^ has been developed. The newly synthesized compounds were submitted for testing for antiproliferative activity against the NCI-60 cell line. All newly synthesized compounds **4a**–**j**, **5**, and **6** were selected for testing against a panel of sixty cancer cell lines at a single concentration of 10 µM. Some compounds tested demonstrated remarkable antiproliferative activity against the cell lines tested. Compounds **4c**, **4e**, and **4g** were chosen for five-dose testing against 60 human tumor cell lines. Compound **4c** demonstrated strong selectivity against the leukemia subpanel, with a selectivity ratio of 5.96 at the GI_50_ level. The most effective in vitro anti-cancer assay derivatives (**4c**, **4d**, **4e**, **4g**, and **4h**) were tested for EGFR and BRAF^V600E^ inhibition as potential targets for antiproliferative action. The results revealed that compounds **4c** and **4e** have significant antiproliferative activity as dual EGFR/BRAF^V600E^ inhibitors. Compounds **4c** and **4e** induced apoptosis by increasing caspase-3, caspase-8, and Bax levels while decreasing the anti-apoptotic Bcl2 protein. Moreover, molecular docking studies confirmed the potential of compounds **4c** and **4e** to act as dual EGFR/BRAF^V600E^ inhibitors.

## 1. Introduction

Cancer has become a global burden due to the significant increase in cancer incidence and mortality rates [1,2]. Tumor resistance, drug toxicity, cancer recurrence, and the inadequate success rate of drug development reaching clinical trials are all limiting variables that exacerbate the difficulties in cancer treatment [3,4]. The search for novel classes of anti-cancer medications focuses on enhancing cancer patients’ treatment efficacy and survival rates. The usual “one-size-fits-all” approach of standard non-targeting medicines damages healthy cells and may not benefit all patients [5,6]. Because cancer is a major focus of the precision medicine program, customized therapy approaches targeted at optimizing outcomes based on individual variability in genetic profile, lifestyle, and environmental factors are gaining popularity [7]. As a result, targeted therapy is the cornerstone for precision medicine, allowing for individualized treatment targeting specific tumor oncogenic markers.

Due to their different biological properties and clinical uses, various benzimidazole compounds have recently attracted attention in anti-cancer agent research [8]. Because of its unique core structure and low toxicity, benzimidazole is an ideal scaffold in the development of anti-cancer drugs [9,10]. Benzimidazole is a bioactive heterocyclic molecule that is one of the top ten most commonly used five-membered nitrogen heterocycles in US Food and Drug Administration (FDA)-approved medications [11]. The electron-rich nitrogen heterocycles of benzimidazole may rapidly receive or donate protons and easily facilitate the creation of different weak contacts, giving it an advantage in binding with a wide range of therapeutic targets and demonstrating broad-ranging pharmacological effects [12,13,14]. A broad pharmacological profile of benzimidazole and its derivatives, substitution at the 1, 2, 5, and/or 6 positions, has been documented in numerous categories of medicinal agents with distinct features, with anti-cancer activity topping the list [8,15].

Benzimidazole and its derivatives have been reported to play important roles as topoisomerase inhibitors, DNA intercalation and alkylating agents, androgen receptor antagonists, poly (ADP-ribose) polymerase (PARP) inhibitors, protein kinase inhibitors, dihydrofolate reductase inhibitors, and microtubule inhibitors [16,17].

**Figure 1:**
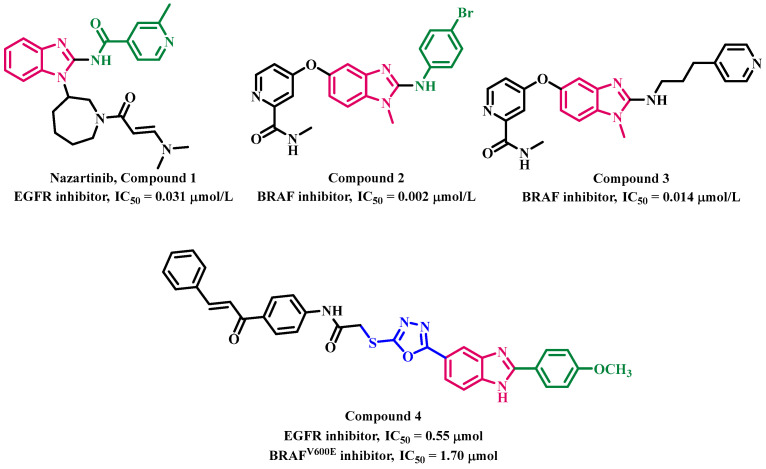
Structures of compounds **1**–**4**.

Kinases are enzymes that mediate the signaling cascade and regulate molecular functions and biological processes like growth, proliferation, differentiation, and apoptosis. The human kinome has roughly 535 protein kinases, which can be divided into tyrosine, serine/threonine, and tyrosine kinase-like enzymes [18,19]. Tyrosine kinases (TKs) include both receptor tyrosine kinases (RTKs) (such as EGFR, FGFR, and PDGFR) and non-receptor tyrosine kinases (NRTKs) (such as ABL, FBK, and SRC). Serine/threonine kinases include BRAF/MEK/ERK, CDKs, and PI3K/AKT/mTOR [20]. A deficiency in cellular kinase phosphorylation can eventually lead to constitutive activation of kinases in live cells, promoting the development of malignancy and tumor progression [18,21]. The benzimidazole scaffold is commonly used as a template for synthesizing kinase inhibitors, as shown in Figure 1.

Nazartinib (**1**, Figure 1) is an irreversible, third-generation EGFR TKI that targets EGFR-activating mutations [22,23]. Nazartinib contains dimethylamino crotonamide, which is known as the optimal group for several covalent pan-EGFR inhibitors, as well as a racemic 3-substituted azepane linker and a chloro substituent at the benzene ring of the benzimidazole nucleus, which contributes to its improved solubility, oral bioavailability, selectivity, and high affinity for EFGR [24,25]. In xenograft models, nazartinib inhibits EGFR signaling and the MAPK pathway, inducing cell cycle arrest, apoptosis, and tumor regression [26]. Nazartinib is now being tested in a phase I/II clinical trial in patients with EGFR-mutant non-small cell lung cancer [22].

Ramurthy and colleagues reported the anti-BRAF^V600E^ activity of two benzimidazole-based compounds, **2** and **3** (Figure 1), with IC_50_ values of 0.002 and 0.014 mmol/L, respectively [27]. The rapidly accelerated fibrosarcoma (RAF) oncogene is associated with cancer cellular processes such as proliferation, invasion, metastasis, and cell survival. Both **2** and **3** can decrease the proliferation of SK-MEL-28 melanoma cells containing B-RAF^V600E^ proteins [27]. Ramurthy and colleagues demonstrated that including a methyl group at the NH of benzimidazole increases the binding affinity to RAF kinase compared to the unmethylated homolog.

Recently [10], we reported on the design and synthesis of a novel series of 1,3,4-oxadiazole/benzimidazole chalcones as antiproliferative agents inducing apoptosis with dual inhibitory effect against EGFR and BRAF^V600E^. Compound **4** (Figure 1) was shown to be the most potent antiproliferative agent, with a GI_50_ value of 1.20 µM against four cancer cell lines, when compared to the reference doxorubicin, which had a GI_50_ value of 1.10 µM. Compound **4** was tested for inhibitory efficacy against EGFR and BRAF^V600E^ as prospective antiproliferative targets. Compound **4** inhibited both targets significantly, with IC_50_ values of 0.55 ± 0.10 µM and 1.70 ± 0.20 µM, respectively, when compared to the reference erlotinib IC_50_ values of 0.08 ± 0.01 and 0.06 ± 0.01 µM, respectively.

**Figure 2:**
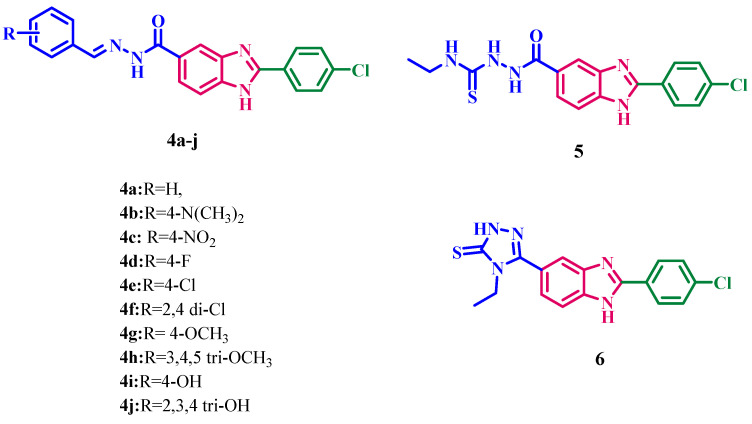
Structures of new targets **4a**–**j**, **5**, and **6**.

Motivated by the promising anti-cancer activity of some benzimidazole-based derivatives and as part of our ongoing effort to develop a dual or multi-targeted anti-cancer agent [28,29,30,31,32,33], we present here the design, synthesis, and antiproliferative activity of a novel series of benzimidazole-based derivatives (**4a**–**j**, **5**, and **6**, Figure 2). NCI chose all newly synthesized compounds for an in vitro one-dose inhibitory experiment against a panel of 60 cancer cell lines. The most active compounds were chosen for five-dose experiments and mechanistic studies against two potential targets, EGFR and BRAFV^600E^. Furthermore, the most effective derivatives were tested for apoptosis-inducing activity against caspase-3, caspase-8, Bax, and anti-apoptotic Bcl2. Finally, docking analyses were undertaken for the most potent derivatives to evaluate their binding mechanisms and interactions with the selected receptors.

## 2. Results and Discussion

### 2.1. Chemistry

**Scheme 1:**
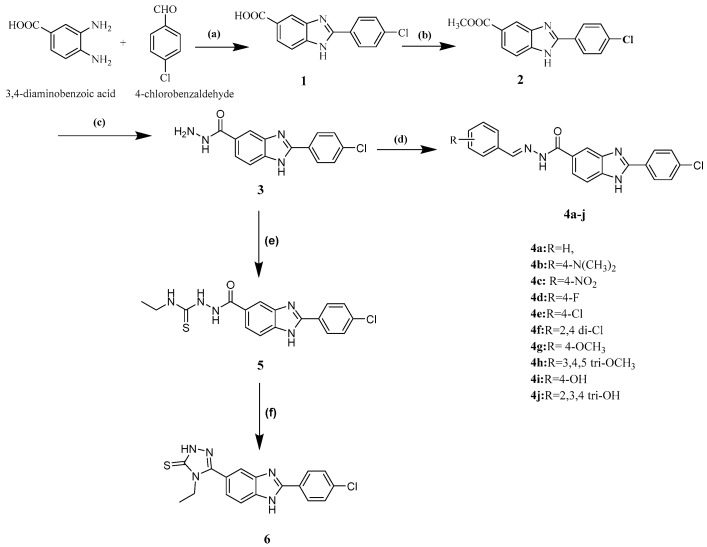
Synthetic steps for compounds **4a**–**j**, **5**, and **6**.

Scheme 1 depicts the synthesis method for compounds **4a**–**j**, **5**, and **6**. Condensation of 3,4-aminobenzoic acid with p-chlorobenzaldehyde in the presence of sodium metabisulphite and DMF resulted in the synthesis of benzimidazole-5-carboxylic acid (**1**) in a good yield [34,35]. Compound **1**’s infrared spectra showed that C=O was present at 1662 cm^−1^. Furthermore, a prolonged peak at 2683–3358 cm^−1^ was identified as the COOH group’s OH. Furthermore, the ^1^HNMR spectra of sample 1 showed the existence of seven aromatic protons at δ 7.63–8.20 ppm and a singlet signal at δ 13.32 ppm that is attributable to the COOH proton, which is exchangeable with D_2_O. The Fischer esterification of carboxylic acid **1** was accomplished using methanol and a few drops of conc. H_2_SO_4,_ and the mixture was heated at reflux for 17 h to obtain compound **2** in 60% yield [36,37]. Compound 2’s IR spectra showed that the OH peak vanished and the C=O group shifted to 1716 cm^−1^ as a result of ester production. Compound **2**’s ^1^HNMR spectra revealed a singlet peak associated with CH_3_ at 3.87 ppm. The hydrazide **3** was prepared in 58% yield by heating the ester **2** at reflux with 99% hydrazine monohydrate in ethanol [38]. Compound’s **3** IR spectra revealed forked peaks in the infrared spectrum corresponding to the NH_2_ and NH protons, respectively, at 3316, 3292, and 3423cm^−1^. In addition,, ^1^H NMR spectra revealed that the methyl protons signal vanished and singlet signals appeared at δ: 4.46 and 9.81 ppm, corresponding to NH2 and NH, respectively. The target compounds **4a**–**j** were prepared in high yield by heating the hydrazide **3** at reflux with substituted benzaldehyde in absolute ethanol, catalyzed by a few drops of glacial acetic acid. The structure of hydrazone derivatives **4a**–**j** was confirmed using different spectroscopic techniques. The IR spectra of **4a**–**j** exhibited the disappearance of forked NH_2_ and the presence of absorption bands at υ_max_; 3423–3225 and at υ_max_; 1441–1566 cm^–1^, corresponding to NH and C=N, respectively. The ^1^H NMR spectra of compounds **4a**–**j** revealed the disappearance of the NH_2_ proton at δ: 4.46–5.66 ppm and the presence of two signals at δ: 8.32–8.86 and δ: 11.60–13.41 ppm attributed to azomethine proton CH=N and hydrazone NH, respectively. Moreover, the ^13^C NMR spectra of **4a**–**j** revealed the presence of a new signal at δ: 161.21–164.69 ppm related to hydrazone C=N carbon.

**Reagent and reaction conditions:** (a) DMF, Na_2_S_2_O_8_; (b) conc. H_2_SO_4,_ methanol, reflux; (c) NH_2_NH_2_, H_2_O; (d) aldehyde, CH_3_COOH; (e) ethyl isothiocyanate, ethanol; (f), NaOH.

The thiosemicarbazide **5** was prepared via heating at reflux of the hydrazide **3** with ethyl isothiocyanate in absolute ethanol [39]. The 1,2,4-triazole-3-thione **6** was synthesized by cyclization of the 1,4-disubstituted thiosemicarbazide **5** in the presence of 2N sodium hydroxide solution followed by acidification with hydrochloric acid until pH = 3. The proposed mechanism for forming 1,2,4-triazole derivative **6** is through the nucleophilic addition of terminal nitrogen atoms to the carbonyl group; then, the species eliminate water to afford the triazole product **6**.

The chemical structure of compound **6** was elucidated by spectroscopic techniques and elemental analyses. The IR spectrum of **6** exhibited a band at υ_max_: 3201–3361 cm^−1^ and υ_max_: 1501–1537 cm^−1^, corresponding to NH of triazole and C=S, respectively. The ^1^H NMR spectrum of the target compound **6** revealed an exchangeable signal at δ: 13.96–14.36 ppm, attributed to NH triazole, and a triplet-quartet pattern was observed at δ: 1.16–1.20 and δ: 4.08–4.15 ppm, attributed to the ethyl protons. The ^13^C NMR spectrum of compound 6 revealed the presence of a signal at δ: 166.69 ppm related to C=S carbon and at δ: 13.91, 18.93 related to CH_3_ and CH_2_ moieties.

### 2.2. Biology

#### 2.2.1. In Vitro Anti-Cancer Activity

The final compounds were submitted to the National Cancer Institute, Bethesda, USA, for screening in vitro for anti-cancer [40,41]. Eleven compounds, **4a**–**j**, **5**, and **6,** were selected according to the Development Therapeutic Program (DTP) protocol. The selected compounds were evaluated for their in vitro cytotoxic activity against a panel of sixty cancer cell lines (NCI-60 cell line panel) derived from six cell lines panel of leukemia, nine cell lines of lung cancer, six cell lines of CNS cancer, seven cell lines of colon cancer, eight cell lines of melanoma, six cell lines of ovarian cancer, eight cell lines of renal cancer, two cell lines of prostate cancer, and eight cell lines of breast cancer, representing the full nine human systems as leukemia, melanoma, and cancers of the lung, colon, brain, breast, ovary, kidney, and prostate.

##### In Vitro One-Dose Assay

The selected compounds **4a**–**j**, **5**, and **6** were tested at a single concentration of 10 µM. The screening results for the selected compounds are illustrated in Table 1.

The screening results of the target compounds revealed that benzimidazole-bearing hydrazone derivatives **4a**–**j** have more potent anti-cancer activity than triazole derivatives **6**. Among hydrazone derivatives, compounds **4c**, **4d**, **4e**, **4f**, **4g**, **4h**, and **4j** showed promising anti-cancer activity in a one-dose assay at 10 µM.

Compound **4c** revealed significant to complete cell death against the leukemia cell line with cell inhibition percentages of 90.13, 89.22, 93.42, 90.32, 95.76, and 108.49. Moreover, it showed remarkable anti-cancer activity against non-small cell lung cancer with cell inhibition percentages of 65.46, 50.97, 55.22, 40.32, 45.22, 83.75, 94.56, and 66.97. In addition, it displayed significant anti-cancer activity against colon cancer with cell inhibition percentages of 97.72, 85.09, 85.40, 70.12, 91.75, 86.97, and 87.34. In addition, it exhibited moderate to significant anti-cancer activity against the CNS cancer cell line with cell inhibition percentages of 51.50, 78.32, 58.34, 47.95, 30.71, and 77.67. It also showed moderate to complete cell death against melanoma with cell inhibition percentages of 100.91, 92.21, 74.09, 52.42, 33.28, 54.65, 97.42, 74.92, and 56.35. Likewise, it displayed moderate to significant anti-cancer activity against ovarian cancer with cell inhibition percentages of 80.88, 88.68, 76.99, 46.76, 78.40, and 39.50. Furthermore, it exhibited complete cell death against renal cancer with cell inhibition percentages of 50.97, 100.50, 67.71, 57.25, 68.40, 80.35, and 57.44. In addition, it displayed remarkable anti-cancer activity against prostate cancer with cell inhibition percentages of 78.57 and 57.97. Finally, it exhibited significant to complete cell death against breast cancer with cell inhibition percentages of 99.28, 63.24, 85.96, 89.50, and 138.86.

Compound **4d** exhibited moderate to significant cytotoxic activity with cell inhibition percentages of 78.63, 32.55, 62.62, 61.21, 76.45, 79.32, 82.16, 39.48, 38.62, 63.77, 58.71, 51.35, 95.67, 35.11, 53.71, 68.72, 39.59, 98.38, 65.96, 88.22, 39.75, 59.83, 63.60, 48.73, 87.83, 64.33, 58.81, 53.21, 82.56, 36.06, and 40.63 against CCRF-CEM, RPMI-8226, HL-60, K-562, MOLT-4, SR, A549, HOP-62, NCI-H23, NCI-H460, COLO 205, HCT-16, HT 29, KM12, SW-620, SF-295, SNB-19, U251, LOXIMVI, MALME-3M, M14, IGROV1, OVCAR-4, OVCAR-8, 786-O, ACHN, SN12C, PC-3, MCF7, MDA-MB-231, and T-47D.

Compound **4e** revealed significant to complete cell death against leukemia with cell inhibition percentages of 84.49, 97.17, 94.23, 91.33, 95.55, and 124.18. In addition, it exhibited moderate to complete cell death against non-small cell lung cancer with cell inhibition percentages of 126.66, 91.77, 91.95, 71.87, 100.37, 72.31, 133.56, and 36.76. Moreover, it displayed significant to complete cell death against colon cancer with cell inhibition percentages of 123.92, 96.31, 101.88, 95.31, 98.33, 129.12, and 128.29. Furthermore, it showed remarkable complete cell death against CNS cancer with cell inhibition percentages of 62.03, 149.27, 62.90, 95.65, and 115.25. Additionally, it exhibited remarkable complete cell death against melanoma with cell inhibition percentages of 155.86, 158.65, 80.33, 61.19, 61.09, 109.23, 63.84, 68.26, and 83.15. It also revealed remarkable complete cell death against ovarian cancer with cell inhibition percentages of 127.35, 152.88, 117.88, 76.28, 85.77, 58.78, and 50.18. In addition, it showed moderate to complete cell death against renal cancer with cell inhibition percentages of 142.12, 43.85, 120.18, 38.77, 81.08, 136.90, 104.33, and 78.42. Finally, it displayed remarkable complete cell death against prostate and breast cancers with cell inhibition percentages of 124.90, 69.46, 119.97, 90.52, 67.05, 113.10, and 144.13.

Compound **4f** exhibited remarkable anti-cancer activity against leukemia cell lines such as CCRF-CEM, K-562, MOLT-4, and SR with cell inhibition percentages of 48.15, 53.70, 72.64, and 54.59. In addition, it showed moderate cytotoxic activity with cell inhibition percentages of 41.02, 31.01, and 36.67 against U251, IGROV1, and A498.

Compound **4g** induced moderate to significant cytotoxic activity with cell inhibition percentages of 34.69, 40.78, 68.14, 31.79, and 99.35 against different leukemia cell lines. Furthermore, it displayed remarkable anti-cancer activity against different cell lines of non-small cell lung cancer with cell inhibition percentages of 88.04, 70.56, 69.68, 64.03, 58.45, 44.09, and 94.09. In addition,, it showed moderate to significant anti-cancer activity against colon cancer with cell inhibition percentages of 77.14, 48.89, 81.02, 42.48, 78.10, 82.72, and 75.58. Moreover, it exhibited a remarkable complete cell death against CNS cancer with cell inhibition percentages of 80.07, 138.85, 57.39, 75.84, and 92.04. Likewise, it revealed moderate to significant cytotoxic activity with cell inhibition percentages of 99.12, 93.47, 73.78, 31.49, 35.90, 87.53, 66.34, 45.62, and 69.11 against different melanoma cell lines. It also exhibited significant to complete cell death against ovarian cancer with cell inhibition percentages of 85.41, 79.87, 130.63, and 68.70. Additionally, it showed moderate to significant cytotoxic activity with cell inhibition percentages of 96.85, 33.43, 78.11, 39.51, 56.23, 93.76, and 60.87 against different renal cancer cell lines. Finally, it displayed remarkable complete cell death against prostate and breast cancer with cell inhibition percentages of 104.40, 83.33, 67.67, 43.07, 76.71, and 75.82. Compound IVo induced moderate cytotoxic activity with cell inhibition percentages of 44.21 and 44.91 against MALME-3M and T-47D.

In the same context, compound **4h** exhibited remarkable anti-cancer activity against leukemia with cell inhibition percentages of 63.44, 80.13, 55.12, 67.33, 58.45, and 54.32. Furthermore, it showed moderate to remarkable cytotoxic activity with cell inhibition percentages of 46.27, 54.34, 63.83, 60.07, and 38.17 against non-small cell lung and colon cancer. Additionally, it induced moderate to remarkable anti-cancer activity against CNS cancer with cell inhibition percentages of 55.97, 56.23, and 46.76. Furthermore, it displayed moderate to significant cytotoxic activity with cell inhibition percentages of 49.36, 71.57, 57.97, 35.98, 53.48, 58.08, 45.65, and 71.28 against different melanoma cell lines. Moreover, it showed moderate to complete cell death against different cell lines of ovarian, renal, prostate, and breast cancer with cell inhibition percentages of 31.57, 38.44, 39.48, 48.80, 101.95, 32.27, 63.67, 31.47, 40.06, 87.88, 51.70, and 84.11.

In the same context, compound **4j** exhibited moderate to significant cytotoxic activity with cell inhibition percentages of 45.33, 51.12, 59.21, 51.83, 59.69, 56.38, 43.95, 32.23, and 90.44 against CCRF-CEM, SR, HOP-62, NCI-H226, NCI-H322M, SF-268, U251, OVCAR-3, and MDA-MB-468. In conclusion, different substitutions of aldehydes such as Cl, NO_2,_ and OCH_3_ have significant anti-cancer activity.

##### In Vitro Five-Dose Assay

**Figure 3:**
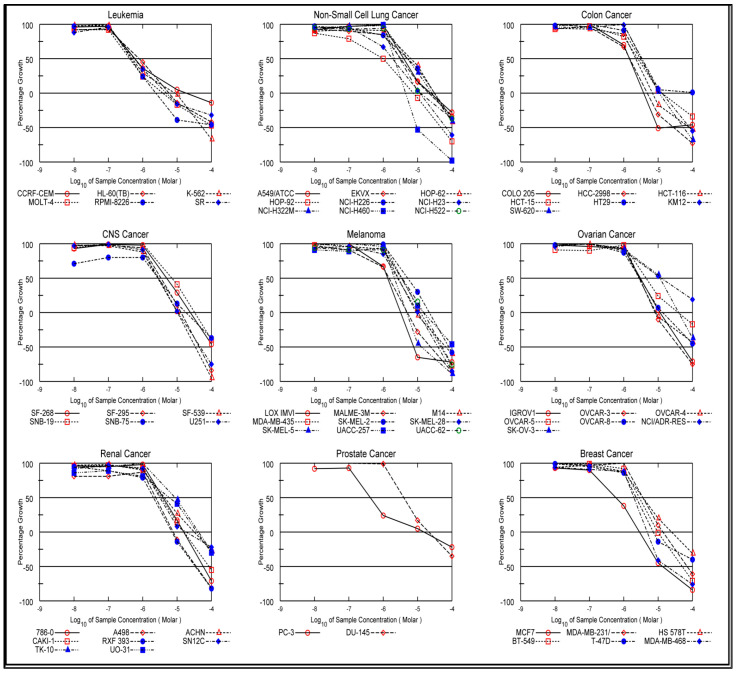
Dose–response curves for all cell lines for compound **4c**.

**Figure 4:**
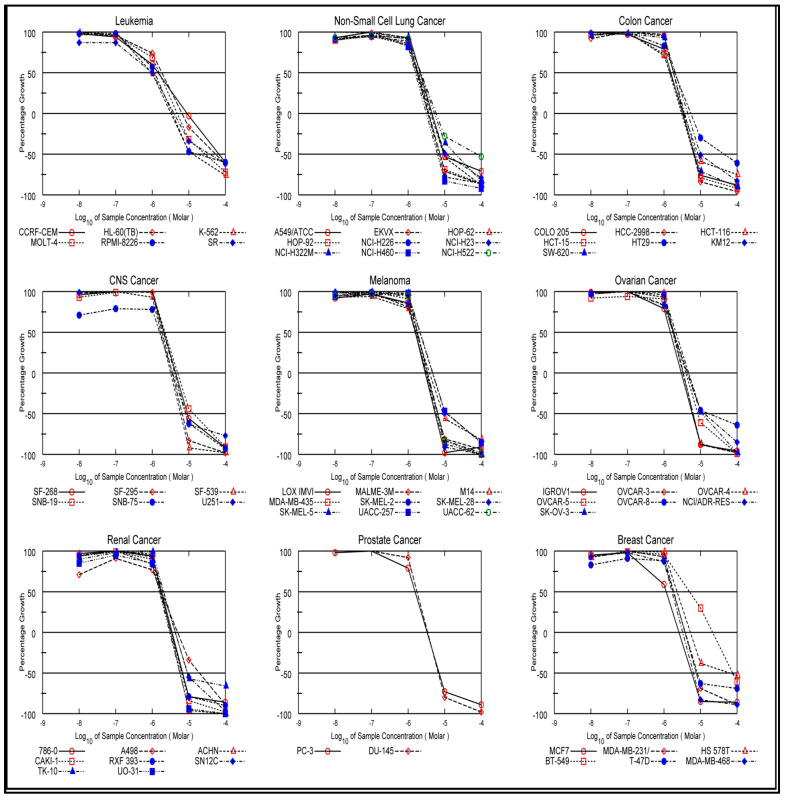
Dose–response curves for all cell lines for compound **4e**.

**Figure 5:**
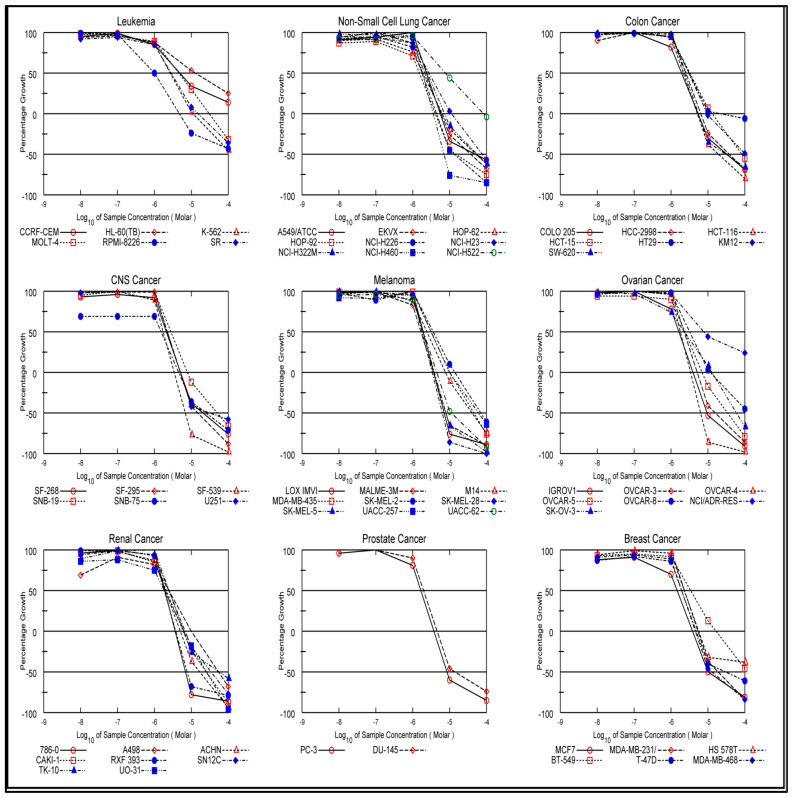
Dose–response curves for all cell lines for compound **4g**.

Compounds **4c**, **4e**, and **4g** were selected for advanced five-dose testing against the full panel of 60 human tumor cell lines [42,43], which represents nine tumor subpanels screened at five concentrations (0.01, 0.1, 1, 10, and 100 µM). The results are used to construct a dose-response curve log of concentration versus percentage growth inhibition (Figure 3, Figure 4 and Figure 5) and calculate three response parameters for each cell line (GI_50_, TGI, and LC_50_). The GI_50_ value (concentration of maximum 50% cell proliferation inhibition) relates to compound concentration causing inhibition by 50% in total cell growth, the TGI value (cytostatic activity) refers to the concentration of the compound resulting in a reduction of total growth, and the LC_50_ value (cytotoxic activity) indicates the concentration of the compound causing a net 50% inhibition of initial cells at the end of the incubation period of 48 h.

The standard for a compound’s selectivity depends on the ratio resulting from dividing the entire MID panel (the average sensitivity of all cell lines to the test agent) (μM) by its individual MID (μM) subpanel. Ratios between 3 and 6 indicate moderate selectivity; ratios > 6 refer to high selectivity towards the respective cell line, whereas compounds that do not meet either of these criteria are rated nonselective [44]. In vitro five-dose assay screening results are illustrated in Table 2, Table 3 and Table 4.

Compounds **4c**, **4e**, and **4g** achieved complete cell death for different cancer cell lines. These compounds were thus selected for five-dose testing to screen their activity at five different concentrations. In addition, these compounds have a broad and potent spectrum of anti-cancer activity without selectivity toward any of the tested cancer cell lines.

Compound **4c** revealed a GI_50_ value ranging from 0.420 to 8.99 µM toward the majority of the examined cell lines, a selectivity ratio from 0.57 to 5.96, a TGI from 2.41 to >100 µM, and a LC_50_ from 9.61 to >100 µM. Compound **4c** showed moderate selectivity toward leukemia cell lines as the ratio between selectivity towards cancer subpanels = 5.96 (Table 2 and Figure 3).

In the same context, compound **4e** was found to have broad-spectrum cell growth inhibition activity against most of the tested tumor subpanels, with GI_50_ values ranging from 0.97 to 4.93 µM, selectivity ratio ranging from 0.79 to 1.35, TGI from 2.13 to 9.03 µM, and LC_50_ from 5.73 to 77.90 µM (Table 3 and Figure 4).

Finally, compound **4g** exhibited potent antitumor activity toward all the examined cell lines, with GI_50_ values ranging between 0.997 and 7.81 µM, selectivity ratio ranging from 0.87 to 1.39, TGI from 1.64 to >100 µM, and LC_50_ between 6.33 and >100 µM (Table 4 and Figure 5).

#### 2.2.2. EGFR Inhibitory Assay

The most effective derivatives from in vitro anti-cancer assay (**4c**, **4d**, **4e**, **4g**, and **4h**) were tested for EGFR inhibition as a potential target for antiproliferative action [45,46]. Table 5 shows the results as IC_50_ values versus erlotinib as the control drug.

The results showed that the investigated compounds had potential EGFR inhibitory action, with IC_50_ values ranging from 0.09 to 0.68 µM compared to erlotinib, which has an IC_50_ value of 0.08 µM. Furthermore, the findings of this investigation are consistent with those of an in vitro five-dose inhibition assay in which compound **4e** was the most potent EGFR inhibitor with an IC_50_ value of 0.09 µM, equivalent to the reference erlotinib. Compound **4c** was 1.2-fold less potent than **4e**, having an IC_50_ value of 0.11 µM. Compounds **4d**, **4g**, and **4h** inhibited EGFR in a weak to moderate approach, with IC_50_ values of 0.68 µM, 0.27 µM, and 0.56 µM, respectively, being seven-fold, three-fold, and six-fold less effective than **4e**. These findings suggested that compounds **4c** and **4e** could be promising antiproliferative agents that target the EGFR inhibitory pathway.

#### 2.2.3. BRAF^V600E^ Inhibitory Assay

Compounds **4c**, **4d**, **4e**, **4g,** and **4h** were investigated further as possible BRAF^V600E^ inhibitors [47]. Table 2 displays the IC_50_ values compared to erlotinib and vemurafenib, which were used as controls. According to Table 5, the compounds tested showed considerable BRAFV^600E^ suppressive action, with IC_50_ values ranging from 0.20 to 0.85 µM. In all cases, the tested derivatives were less potent than erlotinib (IC_50_ = 0.06 µM) and vemurafenib (IC_50_ = 0.03 µM).

Compounds **4c** and **4e**, which were the most potent derivatives in the antiproliferative and EGFR suppressive assays, were also the most effective derivatives as anti-BRAF^V600E^, with IC_50_ values of 0.31 ± 0.07 µM and 0.20 ± 0.02 µM, respectively. These findings suggest that compounds **4c** and **4e** have significant antiproliferative activity as dual EGFR/BRAFV^600E^ inhibitors, implying that additional structural modifications may be required to develop a more potent lead molecule for future development.

#### 2.2.4. Apoptosis Induction Assays

One method of treating cancer is to regulate or stop the uncontrolled proliferation of cancer cells. Using the natural dying process of the cell is a highly successful strategy. Targeting apoptosis is effective for many types of cancer because apoptosis evasion is a hallmark of cancer that is not particular to the etiology or type of cancer. Many anti-cancer drugs target distinct stages of the intrinsic and extrinsic apoptotic pathways [48,49,50]. Compounds **4c** and **4e**, the most effective derivatives in all in vitro investigations, were investigated for their capacity to initiate apoptosis.

#### Assay for Caspase-3 Activation

Caspase-3 is a key caspase that cleaves numerous cell proteins, resulting in apoptosis [51]. Compounds **4c** and **4e** were tested as activators of caspase-3 against the prostatic cancer (PC-3) cell line, compared to staurosporine as the control drug [52], with the results presented in Table 6. Compounds **4c** and **4e** showed high caspase-3 protein overexpression levels of 365 ± 12 and 470 ± 15 Pg/mL, respectively. They elevated the protein caspase-3 in the PC-3 cancer cell line by around seven to nine times compared to untreated control cells. Compared to reference staurosporine, which had a caspase-3 level overexpression of 260 ± 5 pg/mL, compounds **4c** and **4e** were more active as caspase-3 activators. These findings suggested that the compounds under investigation have apoptotic potential, which could provide additional support for their antiproliferative impact.

##### Assays for Caspase-8, Bax, and Bcl2 Levels

Using staurosporine as a control, compounds 4c and 4e were investigated further for their impact on caspase-8, Bax, and anti-apoptotic Bacl-2 levels against the prostate cancer (PC-3) cell line. Table 3 lists the results. Compound 4e (2.20 ng/mL) had the highest level of caspase-8 overexpression, followed by compound 4c (1.90 ng/mL) and the reference drug staurosporine (1.65 ng/mL). Compounds 4c and 4e, increased caspase-8 levels by 16 and 18 times, respectively, compared to the untreated control cell.

Compared to untreated PC-3 cancer cells, compounds **4c** and **4e** increased Bax levels by 26- and 31-fold (185 pg/mL and 220 pg/mL, respectively). Both are more active than staurosporine, which induces Bax levels up to 170 pg/mL, and a 24-fold increase over untreated cells. Finally, compounds **4c** and **4e** induced equipotent down-regulation of anti-apoptotic Bcl-2 protein levels in the PC-3 cancer cell line when compared to staurosporine. These findings suggest that compounds **4c** and **4e** act as caspase-3, caspase-8, and Bax activators and down-regulators of the anti-apoptotic Bcl-2, classifying them as apoptotic triggers.

## 3. Docking Study

The most active compounds, **4c** and **4e**, have multiple structural options regarding their probable binding mechanism and interactions with EGFR and BRAF active sites. These possibilities were investigated by molecular modeling studies using the Molecular Operating Environment (MOE) software (version 09.2022) [53]. Furthermore, the docking methodology was validated, and the co-crystallized ligand erlotinib, GW572016, was re-docked into the active site using the EGFR domain (PDB ID: 1 M17). On the other hand, the structure of BRAF kinase in a complex with the inhibitor vemurafenib was used to validate the docking protocol for BRAF^V600^ (PDB ID: 3OG7) [54]. With a docking score (S) of −13.4442, the re-docked ligand displayed an RMSD of 0.8429 between the docked pose and the co-crystallized ligand for EGFR. When the co-crystallized ligand was docked, the RMSD between the two was 0.2808 (docking score (S) = −10.2469) for the BRAF^V600^.

The most potent molecules, **4c** and **4e**, were docked into the EGFR’s active site along with erlotinib (as a positive control). Table 7 and Table 8 present the results of the docking experiments together with the kind of interactions, the distances (in Å) between the interacting residues, and the binding affinity (in kcal mol^−1^).

**Figure 6:**
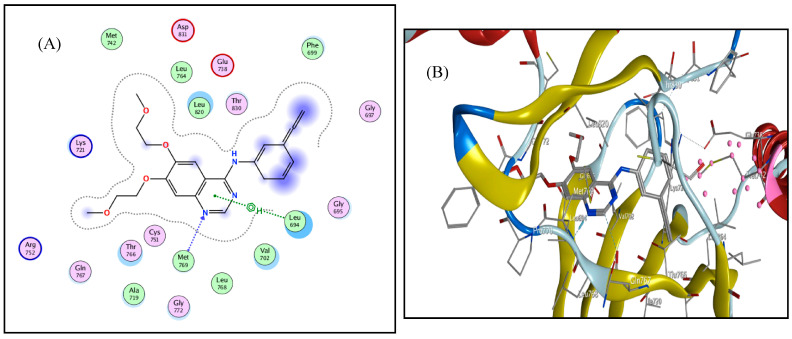
(**A**) 2D interaction, (**B**) 3D interaction of erlotinib inside EGFR binding pocket.

**Figure 7:**
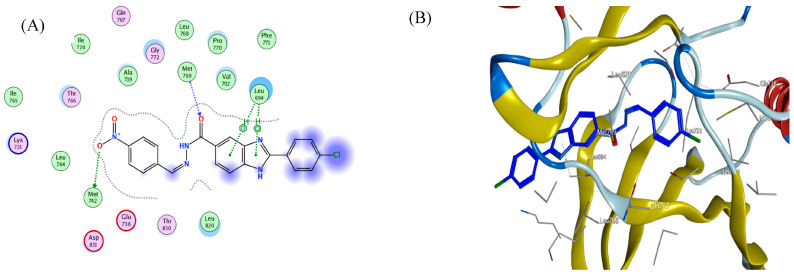
(**A**) 2D interaction, (**B**) 3D interaction of **4c** inside EGFR binding pocket.

**Figure 8:**
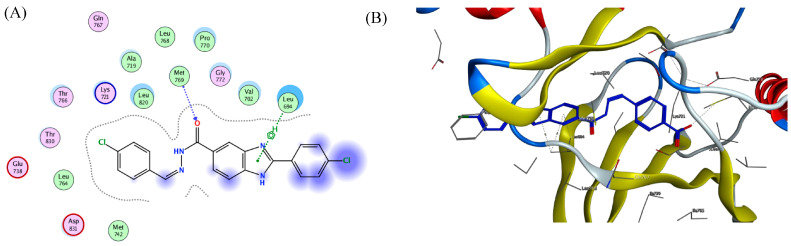
(**A**) 2D interaction, (**B**) 3D interaction of **4e** inside EGFR binding pocket.

Compounds **4c** and **4e** demonstrated substantial interaction with Met793 and other important interacting amino acids required for inhibition of EGFR when compared to co-docked ligand erlotinib as a positive control. Compared to erlotinib, which has a binding affinity of −1.9 kcal mol^−1^, compounds **4c** and **4e** had strong interaction scores of −0.5 and −1.5 kcal mol^−1^, respectively. The many binding interactions with the critical amino acid within the EGFR active site, particularly the gatekeeper Met793 and LEU 694 residues, can account for these significant binding affinities (Figure 6, Figure 7 and Figure 8).

**Figure 9:**
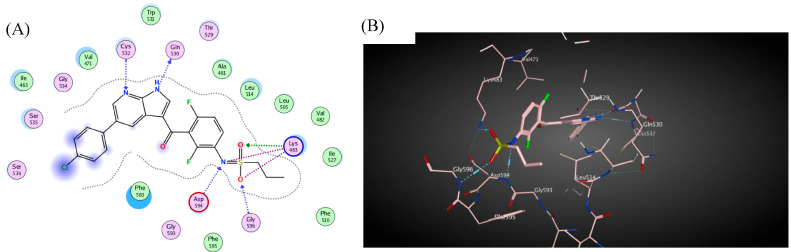
(**A**) 2D interaction, (**B**) 3D interaction of vemurafenib inside BRAF^V600^ binding pocket.

**Figure 10:**
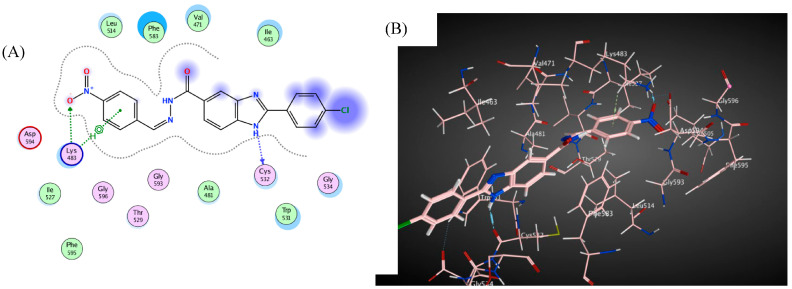
(**A**) 2D interaction, (**B**) 3D interaction of **4c** inside BRAF^V600^ binding pocket.

**Figure 11:**
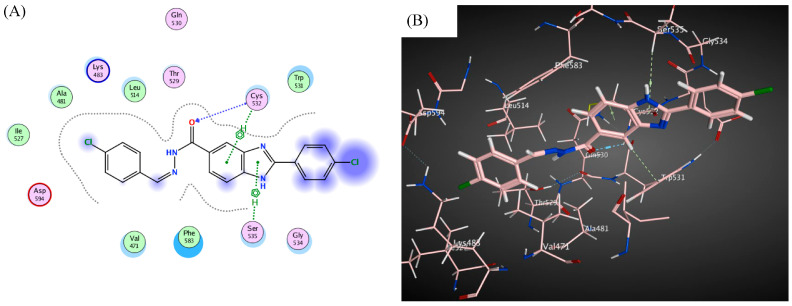
(**A**) 2D interaction, (**B**) 3D interaction of **4e** inside BRAF^V600^ binding pocket.

Additionally, vemurafenib was docked into the BRAF^V600E^ active site among the three most active molecules, **4c** and **4e**. The docking study data are compiled in Table 8, including information on the kind of interactions, binding affinity (in kcal mol^−1^), and distances (in Å) from the interacting residues. A co-docked ligand, vemurafenib, was a positive control in comparing molecules **4c** and **4e**. Strong interactions between these drugs and CYS 532 and other essential interacting amino acids required for BRAF^V600^ were shown. With binding affinities of −5.4 and −1.9 kcal mol^−1^, respectively, compounds **4c** and **4e** showed considerable interaction scores compared to Vemurafenib, which had a binding affinity of −3.5 kcal mol^−1^. The high binding scores of these compounds were established by a significant interaction between the synthesized compounds and the essential amino acid in the BRAF active site, with CYS 532 (Figure 9, Figure 10 and Figure 11).

## 4. Conclusions

Finally, a new class of novel compounds with benzimidazole scaffolds (**4a**–**j**, **5**, and **6**) was synthesized and tested for anticancer activity. NCI chose compounds **4c**, **4e**, and **4g** for five dose tests, with selectivity ratios ranging from 0.60 to 5.80 at the GI_50_ levels. Compounds **4c** and **4e** showed significant EGFR and BRAF^V600E^ inhibition. Apoptotic marker assay results indicate that compounds **4c** and **4e** act as caspase-3, caspase-8, and Bax activators as well as down-regulators of the anti-apoptotic Bcl-2, classifying them as apoptotic triggers. Molecular docking simulations of **4c** and **4e** also revealed good binding modes within the EGFR and BRAF^V600^ active sites. To obtain a more effective lead compound for future development, compounds **4c** and **4e** require further structural modifications as well as the synthesis of more derivatives.

## 5. Experimental

### 5.1. Chemistry

The melting point was obtained utilizing a Thomas-Hoover capillary device and is uncorrected. The infrared (IR) spectra were recorded as films on KBr plates using the FT-IR spectrometer. Thin-layer chromatography was used to evaluate the reaction mixture, homogeneity, and purity of the synthesized compounds (Merck, Darmstadt, Germany).

General Details: See Supplementary Materials.

The key intermediates **1**–**3** were prepared according to previously reported procedures [34,36,38].

#### 5.1.1. General Procedure for Synthesis of N′-(substituted benzylidene)-2-(4-chlorophenyl)-1H-benzimidazole-5-carbohydrazide (**4a**–**j**)

Equimolar quantities of the appropriate compound **3** (2 mmol, 0.50 g) and the appropriate substituted benzaldehyde in absolute ethanol (25 mL) were refluxed for 6 h in the presence of a catalytic amount of glacial acetic acid. The resulting crude solid was filtered off and recrystallized from absolute ethanol to afford compounds **4a**–**j** in a good yield.

##### N′-Benzylidene-2-(4-chlorophenyl)-1H-benzimidazole-5-carbohydrazide (**4a**)

Yellow powder, 80% yield, m.p. 207–209 °C, IR (KBr, υ_max_ cm^−1^): 3426, 3393 (NH benzimidazole, NH hydrazone), 3073 (CH aromatic), 1621 (C=O), 1606 (C=C), 1455 (C=N), 1373–1502 (N-O); ^1^H NMR (DMSO-*d*_6_, 400 MHz, δ ppm): 6.70 (d, 2H, *J =* 8.00 Hz, Ar-2H), 7.45–7.50 (m, 4H, Ar-H), 7.62 (d, 1H, *J =* 8.00 Hz, Ar-H), 7.76 (d, 2H, *J =* 8.00 Hz, Ar-H), 7.88 (d, 2H, *J =* 8.00 Hz, Ar-H), 7.95 (d, 1H, *J =* 8.00 Hz, Ar-H), 8.48 (s, 1H, CH=NH), 11.90 (s, 1H, NH, D_2_O exchangeable), 12.82 (s, 1H, NH benzimidazole, D_2_O exchangeable); ^13^C NMR (DMSO-*d*_6_, 100 MHz, δ ppm):113.65, 116.48, 121.99, 126.37, 127.27, 128.28, 129.15, 129.79, 130.06, 130.98, 133.14, 134.69, 147.10, 151.39, 164.40 (aromatic carbons), 168.14 (C=N), 172.76 (C=O); elemental analysis calculated for C_21_H_15_N_5_O_3_ (M.Wt. 385.38): C, 65.45; H, 3.92; N, 18.17; found C, 65.71; H, 4.13; N, 18.42.

##### N′-(4-(Dimethylamino)benzylidene)-2-(4-chlorophenyl)-1H-benzimidazole-5-carbohydrazide (**4b**)

Buff powder, 80% yield, m.p. 283–285 °C, IR (KBr, υ_max_, cm^−1^): 3385–3226 (NH benzimidazole, NH hydrazone), 3029 (CH aromatic), 1663 (C=O), 1626 (C=C), 1470 (C=N); ^1^H NMR (DMSO-*d*_6_, 500 MHz, δ ppm): 2.96 (s, 6H, 2 CH_3_), 6.76 (d, 2H, *J =* 8.50 Hz, Ar-H), 7.57 (d, 2H, *J =* 8.50 Hz, Ar-H), 7.66 (d, 3H, *J =* 8.50 Hz, Ar-H), 7.81 (d, 1H, *J =* 8.00 Hz, Ar-H), 8.21 (d, 2H, *J =* 8.50 Hz, Ar-H), 8.33 (s, 1H, CH=NH), 11.65 (s, 1H, NH, hydrazone D_2_O exchangeable), 13.42 (s, 1H, NH benzimidazole, D_2_O exchangeable); ^13^C NMR (DMSO-*d*_6_, 125 MHz, δ ppm): 56.54 (CH_3_), 110.95, 111.51, 111.54, 112.27, 122.09, 128.88, 128.94, 129.03, 129.68, 135.33, 135.55, 148.83, 151.38, 151.95, 152.92 (aromatic carbons), 163.43(C=N), 163.73(C=O); elemental analysis calculated for C_23_H_20_ClN_5_O (M.Wt. 417.90): C, 66.11; H, 4.82; N, 16.76; found C, 66.32; H, 4.52; N, 16.99.

##### N′-(4-Nitrobenzylidene)-2-(4-chlorophenyl)-1H-benzimidazole-5-carbohydrazide (**4c**)

Brown powder, 73% yield, m.p. 297–299 °C, IR (KBr, υ_max_, cm^−1^): 3427–3327 (NH benzimidazole, NH hydrazone), 3054 (CH aromatic), 1652 (C=O), 1614 (C=C), 1467 (C=N), 1384-1514 (N-O)**;** ^1^H NMR (DMSO-*d*_6_, 500 MHz, δ ppm): 7.66 (d, 2H, *J =* 8.50 Hz, Ar-H), 7.82–7.85 (m, 1H, Ar-H), 8.01 (d, 2H, *J =* 8.50 Hz, Ar-H), 8.21 (d, 3H, *J =* 9.00 Hz, Ar-H), 8.31 (d, 3H, *J =* 8.50 Hz, Ar-H), 8.57 (s, 1H, CH=NH), 12.25 (s, 1H, NH hydrazone, D_2_O exchangeable), 13.44 (s, 1H, benzimidazole NH, D_2_O exchangeable); ^13^C NMR (DMSO-*d*_6_, 125 MHz, δ ppm): 123.89, 124.18, 124.56, 124.82, 127.90, 128.26, 128.44, 128.85, 128.92, 129.45, 129.70, 135.63, 141.21, 145.36, 148.02 (aromatic carbons), 148.24 (C=N), 164.40 (C=O)**;** elemental analysis calculated for C_21_H_14_ClN_5_O_3_ (M.Wt. 419.83): C, 60.08; H, 3.36; N, 16.68; found C, 60.28; H, 3.54; N, 16.90.

##### N′-(4-Fluorobenzylidene)-2-(4-chlorophenyl)-1H-benzimidazole-5-carbohydrazide (**4d**)

Brown powder, 60% yield, m.p. 285–287 °C, IR (KBr, υ_max_, cm^−1^): 3423–3228 (NH benzimidazole, NH hydrazone), 3061 (CH aromatic), 1663 (C=O), 1625 (C=C), 1470 (C=N); ^1^H NMR (DMSO-*d*_6_, 400 MHz, δ ppm): 7.31 (t, *J =* 7.20 Hz, 2H, Ar-H), 7.66 (d, 3H, *J =* 6.80 Hz, Ar-H), 7.81–7.84 (m, 3H, Ar-H), 8.21 (d, 3H, *J =* 6.80 Hz, Ar-H), 8.48 (s, 1H, CH=NH), 11.97 (s, 1H, NH hydrazone, D_2_O exchangeable), 13.41 (s, 1H, benzimidazole NH, D_2_O exchangeable); ^13^C NMR (DMSO-*d*_6_, 100 MHz, δ ppm): 111.84, 116.31, 116.48, 116.82, 128.53, 128.89, 129.06, 129.70, 129.78, 131.47, 135.60, 146.47, 146.81, 152.91, 162.57(aromatic carbons), 164.18 (C=N), 164.54 (C=O)**;** elemental analysis calculated for C_21_H_14_ClFN_4_O (M.Wt. 392.82): C, 64.21; H, 3.59; N, 14.26; found C, 64.42; H, 3.73; N, 14.39.

##### N′-(4-Chlorobenzylidene)-2-(4-chlorophenyl)-1H-benzimidazole-5-carbohydrazide (**4e**)

Brown powder, 85% yield, m.p. 265–267 °C, IR (KBr, υ_max_, cm^−1^): 3434–3393 (NH benzimidazole, NH hydrazone), 3086 (CH aromatic), 1640 (C=O), 1604 (C=C), 1489 (C=N); ^1^H NMR (DMSO-*d*_6_, 500 MHz, δ ppm): 7.53 (d, 2H, *J =* 8.50 Hz, Ar-H), 7.66 (d, 2H, *J =* 8.50 Hz, Ar-H), 7.71–7.73 (m, 1H, Ar-H), 7.78 (d, 2H, *J =* 8.50 Hz, Ar-H), 7.83–7.84 (m, 1H, Ar-H), 8.21 (d, 3H, *J =* 8.50 Hz, Ar-H), 8.47 (s, 1H, CH=NH), 12.02 (s, 1H, NH hydrazone, D_2_O exchangeable), 13.40 (s, 1H, benzimidazole NH, D_2_O exchangeable); ^13^C NMR (DMSO-*d*_6_, 125 MHz, δ ppm): 128.25, 128.90, 129.19, 129.42, 129.69, 129.78, 131.60, 131.67, 132.98, 133.80, 134.70, 134.93, 135.60, 146.59, 152.65 (aromatic carbons), 164.21(C=N), 172.72(C=O); elemental analysis calculated for C_21_H_14_Cl_2_N_4_O (M.Wt. 409.27): C, 61.63; H, 3.45; N, 13.69; found C, 61.91; H, 3.32; N, 13.70.

##### N′-(2,4-Dichlorobenzylidene)-2-(4-chlorophenyl)-1H-benzimidazole-5-carbohydrazide (**4f**)

Yellow powder, 79% yield, m.p. 294–297 °C, IR (KBr, υ_max_, cm^−1^): 3459-3393 (NH benzimidazole, NH hydrazone), 3025 (CH aromatic), 1663 (C=O), 1620 (C=C), 1467 (C=N); ^1^H NMR (DMSO-*d*_6_, 500 MHz, δ ppm): 7.52 (d, 1H, *J =* 8.50 Hz, Ar-H), 7.64–7.70 (m, 4H, Ar-H), 7.85 (s, 1H, Ar-H), 8.06 (d, 1H, *J =* 8.50 Hz, Ar-H), 8.20 (d, 3H, *J =* 8.00 Hz, Ar-H), 8.84 (s, 1H, CH=NH), 12.22 (s, 1H, NH hydrazone, D_2_O exchangeable), 13.41 (s, 1H, benzimidazole NH, D_2_O exchangeable); ^13^C NMR (DMSO-*d*_6_, 125 MHz, δ ppm): 111.75, 127.19, 128.48, 128.56, 128.86, 128.91, 129.29, 129.59, 129.68, 129.81, 131.23, 134.29, 135.36, 135.49, 135.61, 138.10, 142.74 (aromatic carbons), 164.20(C=N, 172.42(C=O); elemental analysis calculated for C_21_H_13_Cl_3_N_4_O (M.Wt. 443.71): C, 56.85; H, 2.95; N, 12.63; found C, 56.99; H, 3.19; N, 12.91.

##### N′-(4-Methoxybenzylidene)-2-(4-chlorophenyl)-1H-benzimidazole-5-carbohydrazide (**4g**)

Yellow powder, 82% yield, m.p. 273–275 °C, IR (KBr, υ_max_, cm^−1^): 3449–3390 (NH benzimidazole, NH hydrazone), 3029 (CH aromatic), 2951 (CH aliphatic), 1661 (C=O), 1605 (C=C), 1466 (C=N); ^1^H NMR (DMSO-*d*_6_, 500 MHz, δ ppm): 3.85 (s, 3H, OCH_3_), 7.02 (d, 2H, *J =* 8.00 Hz, Ar-H), 7.65 (d, 2H, *J =* 8.50 Hz, Ar-H), 7.69-7.72 (m, 3H, Ar-H), 7.81–7.83 (m, 1H, Ar-H), 8.21 (d, 3H, *J =* 8.50 Hz, Ar-H), 8.42 (s, 1H, CH=NH), 11.87 (s, 1H, NH hydrazone, D_2_O exchangeable); ^13^C NMR (DMSO-*d*_6_, 125 MHz, δ ppm): 55.91 (OCH_3_), 114.42, 114.66, 123.25, 126.83, 127.17, 128.21, 128.79, 128.98, 129.58, 130.72, 132.33, 135.42, 147.61, 152.57, 161.43 (aromatic carbons), 163.93(C=N), 172.79(C=O); elemental analysis calculated for C_22_H_17_ClN_4_O_2_ (M.Wt. 404.85): C, 65.27; H, 4.23; N, 13.84; found C, 65.00; H, 4.03; N, 13.98.

##### N′-(3,4,5-Trimethoxybenzylidene)-2-(4-chlorophenyl)-1H-benzimidazole-5 carbohydrazide (**4h**)

Yellow powder, 84% yield, m.p. 288–290 °C, IR (KBr, υ_max_, cm^−1^): 3440–3318 (NH benzimidazole, NH hydrazone), 3023 (CH aromatic), 2993 (CH aliphatic), 1644 (C=O), 1600 (C=C), 1451 (C=N); ^1^H NMR (DMSO-*d*_6_, 500 MHz, δ ppm): 3.72 (s, 3H, OCH_3_), 3.84 (s, 6H, 2OCH_3_), 7.08 (s, 2H, Ar-H), 7.65 (d, 2H, *J =* 8.50 Hz, Ar-H), 7.71–7.73 (m, 1H, Ar-H), 7.82–7.84 (m, 1H, Ar-H), 8.20-8.23 (m, 3H, Ar-H), 8.41 (s, 1H, CH=NH), 11.98 (s, 1H, NH hydrazone, D_2_O exchangeable); ^13^C NMR (DMSO-*d*_6_, 125 MHz, δ ppm): 56.39 (OCH_3_), 60.60 (OCH_3_), 104.75, 105.98, 122.82, 122.99, 127.84, 128.89, 128.93, 129.68, 130.42, 135.57, 139.52, 147.95, 152.83, 153.63, 153.90 (aromatic carbons), 161.80 (C=N), 164.25 (C=O); elemental analysis calculated for C_24_H_21_ClN_4_O_4_ (M.Wt. 464.91): C, 62.00; H, 4.55; N, 12.05; found C, 62.25; H, 4.82; N, 12.29.

##### N′-(4-Hydroxybenzylidene)-2-(4-chlorophenyl)-1H-benzimidazole-5- carbohydrazide (**4i**)

Yellow powder, 80% yield, m.p. 263–265 °C, IR (KBr, υ_max_, cm^−1^): 3403–3344 (NH benzimidazole, NH hydrazone), 3188 (OH), 3073 (CH aromatic), 1645 (C=O), 1613 (C=C), 1447 (C=N)**;** ^1^H NMR (DMSO-*d*_6_, 500 MHz, δ ppm): 6.85 (d, 2H, *J =* 8.50 Hz, Ar-H), 7.59 (d, 3H, *J =* 8.50 Hz, Ar-H), 7.66 (d, 3H, *J =* 9.00 Hz, Ar-H), 8.21 (d, 3H, *J =* 8.50 Hz, Ar-H), 8.38 (s, 1H, CH=NH), 10.02 (s, 1H, OH, D_2_O exchangeable), 11.72 (s, 1H, NH hydrazone, D_2_O exchangeable), 13.37 (s, 1H, benzimidazole NH, D_2_O exchangeable); ^13^C NMR (DMSO-*d*_6_, 125 MHz, δ ppm): 112.11, 115.56, 116.19, 116.36, 125.87, 127.09, 128.62, 128.89, 129.37, 129.69, 130.02, 130.14, 135.56, 148.29, 159.79 (aromatic carbons), 159.99 (C=N), 163.92 (C=O); elemental analysis calculated for C_21_H_15_ClN_4_O_2_ (M.Wt. 390.83): C, 64.54; H, 3.87; N, 14.34; found C, 64.24; H, 3.67; N, 14.08.

##### N′-(3,4,5-Trihydroxybenzylidene)-2-(4-nitrophenyl)-1H-benzimidazole-5 carbohydrazide (**4j**)

Yellow powder, 84% yield, m.p. 295–297 ^o^C, IR (KBr, υ_max_, cm^−1^): 3462–3423 (NH benzimidazole, NH hydrazone), 3218 (OH), 3054 (CH aromatic), 1650 (C=O), 1615 (C=C), 1480 (C=N); ^1^H NMR (DMSO-*d*_6_, 500 MHz, δ ppm): 6.42 (d, 1H, *J =* 8.50 Hz, Ar-H), 6.80 (d, 1H, *J =* 8.50 Hz, Ar-H), 7.66 (d, 3H, *J =* 8.50 Hz, Ar-H), 7.84 (s, 1H, Ar-H), 8.21 (d, 3H, *J =* 8.50 Hz, Ar-H), 8.49 (s, 1H, CH=NH), 8.58 (s, 1H, OH, D_2_O exchangeable), 9.61 (s, 1H, OH, D_2_O exchangeable), 11.65 (s, 1H, OH, D_2_O exchangeable), 12.06 (s, 1H, NH hydrazone, D_2_O exchangeable), 13.39 (s, 1H, benzimidazole NH, D_2_O exchangeable); ^13^C NMR (DMSO-*d*_6_, 125 MHz, δ ppm): 108.09, 111.36, 111.88, 121.74, 127.17, 128.40, 128.87, 128.91, 129.17, 129.60, 129.69, 133.15, 133.78, 135.62, 147.93, 149.10, 150.38 (aromatic carbons), 163.51(C=N), 172.71(C=O); elemental analysis calculated for C_21_H_15_ClN_4_O_4_ (M.Wt. 422.83): C, 59.65; H, 3.58; N, 13.25; found C, 60.01; H, 3.43; N, 13.51.

#### 5.1.2. General procedure for the synthesis of 2-aryl-1H-benzimidazole-5-carbonyl hydrazine-1-carbothioamide (**5a**)

Equimolar quantities (2 mmol, 0.50 g) of compound **3** and appropriate isothiocyanate in absolute ethanol (25 mL) were heated under reflux for 6 h. The resulting crude solid was filtered off, dried, and used for the following step without further purification.

#### 5.1.3. General procedure for the synthesis of 4-ethyl-5-(2-(4-chlorophenyl)-1H-benzimidazol-5-yl)-2,4-dihydro-3H-1,2,4triazole-3-thione (**6a**)

Appropriate thiosemicarbazide **5a** (10 mmol) was dissolved in 2N sodium hydroxide solution (20 mL), and the resulting solution was stirred under reflux for 4 h. The solution was acidified with dilute HCl until (pH 3); the formed precipitate was filtered off, washed with distilled water, dried, and then recrystallized from ethanol to afford corresponding 1,2,4-triazole **6a** in a good yield.

Yellow powder, 63% yield, m.p. 292–294 °C, IR (KBr, υ_max_, cm^−1^): 3456, 3329 (NH benzimidazole, NH hydrazone), 3133 (CH aromatic), 2863 (CH aliphatic), 1502 (C=S), 1144 (C-N)**;** ^1^H NMR (DMSO-*d*_6_, 500 MHz, δ ppm): 1.16 (t, 3H, *J =* 7.00 Hz, CH_3_), 4.08 (q, 2H, *J =* 7.50 Hz, CH_2_), 6.48 (d, 1H, *J =* 8.50 Hz, Ar-H), 7.64 (d, 2H, *J =* 8.50 Hz, Ar-H), 7.78 (d, 1H, *J =* 8.50 Hz, Ar-H), 7.91 (s, 1H, Ar-H), 8.27 (d, 2H, *J =* 8.50 Hz, Ar-H), 13.96 (s, 1H, NH triazole, D_2_O exchangeable); ^13^C NMR (DMSO-*d*_6_, 125 MHz, δ ppm): 13.91 (CH_3_), 18.93 (CH_2_), 56.50, 120.31, 123.38, 127.26, 128.83, 129.01, 129.48, 129.57, 129.66, 135.57, 152.34, 152.60 (aromatic carbon), 166.96 (C=S ); elemental analysis calculated for C_17_H_14_ClN_5_S (M.Wt. 355.84): C, 57.38; H, 3.97; N, 19.68; S, 9.01; found C, 57.42; H, 4.23; N, 19.74; S, 9.25.

### 5.2. Biology

#### 5.2.1. In Vitro Antiproliferative Activity of Compounds Selected by the NCI

The NCI anti-cancer screening methodology has been described in detail elsewhere (http://www.dtp.nci.nih.gov, accessed on 20 February 2021). Following the Drug Evaluation Branch protocol, National Cancer Institute, Bethesda, USA, a primary anti-cancer assay was performed on approximately 60 human tumor cell line panels derived from nine neoplastic diseases.

#### 5.2.2. Assay for EGFR Inhibitory Effect

Compounds **4c**, **4d**, **4e**, **4g**, and **4h** were investigated for EGFR inhibitory action using erlotinib as the control drug [45]. The detailed methods are described in the Supplementary Materials to this article.

#### 5.2.3. Assay for BRAF^V600E^ Inhibitory Action

Compounds **4c**, **4d**, **4e**, **4g**, and **4h** were tested as possible BRAF^V600E^ inhibitors, compared to erlotinib and vemurafenib as control drugs [47]. Refer to the Supplementary Materials for more details.

#### 5.2.4. Apoptotic Markers Assays

Compounds **4c** and **4e**, the most effective derivatives in all in vitro investigations, were investigated for their capacity to initiate apoptosis through their effect on caspase-3, caspase-8, Bax, and anti-apoptotic Bcl2 [52]. For more details, see Supplementary Materials.

### 5.3. Docking Study

Molecular modelling investigations were conducted using the Molecular Operating Environment (MOE) software version 2022. Compounds 4c and 4e structures were put together in MOE. Furthermore, the protein data bank at the Research Collaboration for Structural Bioinformatics (RSCB) protein database [PDB] (PDB: ID1M17 and 3OG7) provided the X-ray crystal structure of erlotinib and vemurafenib coupled to the EGFR and BRAF^V600^ active site, respectively. Erlotinib and vemurafenib were redocked into the 1M17 and 3OG7 active site to validate the docking strategy. After the co-crystallized ligand and water molecules were eliminated, the enzyme underwent a 3D protonation process in which hydrogen atoms were added to restore its normal geometry, making it ready for docking. Using the GW572016 scoring function, the conformers that were produced were docked into the EGFR and BRAF^V600^ receptor using MOE-dock. The best 50 poses underwent a force field refinement using molecular mechanics [55,56].

## Figures and Tables

**Table 1:** Cell inhibition percentages of NCI-60 cancer cell lines revealed by target compounds **4a**–**j**, **5**, and **6**.

Subpanel Cancer Cell Lines	Cell Inhibition Percent											
	4a	4b	4c	4d	4e	4f	4g	4h	4i	4j	5	6
**Leukemia**												
**CCRF-CEM**	11.20	6.50	**90.13**	**78.63**	**84.49**	**48.15**	**34.69**	**63.44**	13.58	**45.33**	86.90	0
**RPMI-8226**	13.83	5.27	**89.22**	**32.55**	**97.17**	15.43	**40.78**	**80.13**	1.52	5.54	95.85	0.68
**HL-60(TB)**	0	0	**93.42**	**62.62**	**94.23**	5.98	3.67	**55.12**	0	0	0	0
**K-562**	7.37	5.96	**90.32**	**61.21**	**91.33**	**53.70**	**68.14**	**67.33**	0.25	0	88.44	1.43
**MOLT-4**	12.90	3.18	**95.76**	**76.45**	**95.55**	**72.64**	**31.79**	**58.45**	15.60	17.16	90.05	2.85
**SR**	18.05	2.54	**108.49**	**79.32**	**−124.18**	**54.59**	**99.35**	**54.32**	2.26	**51.12**	88.91	17.53
**Non-Small Cell Lung Cancer**												
**A549/ATCC**	0	0	**65.46**	**82.16**	**126.66**	4.70	**88.04**	14.22	0	0	6.17	0
**EKVX**	1.38	2.85	**50.97**	26.79	**91.77**	13.67	**70.56**	4.23	0	82.89	18.21	0.44
**HOP-62**	0	2.51	**55.22**	**39.48**	**91.95**	5.89	**69.68**	18.33	0	**59.21**	0.87	1.71
**HOP-92**	0	0	**40.32**	15.44	**71.87**	0	**64.03**	28.33	0	92.98	1.09	0.32
**NCI-H226**	5.74	4.67	**45.22**	6.86	0.67	18.84	26.17	24.03	20.60	**51.83**	9.79	1.97
**NCI-H23**	1.85	1.51	**83.75**	**38.62**	**100.37**	4.26	**58.45**	**46.27**	4.44	93.41	5.79	4.53
**NCI-H322M**	0	0.24	29.93	22.45	**72.31**	0	**44.09**	12.45	0.47	**59.69**	3.36	0
**NCI-H460**	0	2.77	**94.56**	**63.77**	**133.56**	1.37	**94.09**	17.19	0	97.46	1.06	0
**NCI-H522**	1.00	3.02	**66.97**	3.87	**36.76**	7.79	17.38	21.72	9.85	88.43	5.4	1.85
**Colon Cancer**												
**COLO 205**	0.76	0	**97.72**	**58.71**	**123.92**	0	**77.14**	**54.34**	0.44	0	0	0
**HCC-2998**	0	6.31	**85.09**	20.98	**96.31**	3.25	**48.89**	20.02	0	14.96	5.54	0
**HCT-116**	0	4.70	**85.40**	**51.35**	**101.88**	8.63	**81.02**	**63.83**	0	5.85	0	0
**HCT-15**	4.81	0	**70.12**	33.19	**95.31**	0	**42.48**	3.98	0	4.51	5.90	1.62
**HT29**	3.67	2.26	**91.75**	**95.67**	**98.33**	2.18	**78.10**	**60.07**	0	0	2.33	2.07
**KM12**	3.80	2.41	**86.97**	**35.11**	**129.12**	1.75	**82.72**	**38.17**	2.85	13.12	7.97	0
**SW-620**	0	0	**87.34**	**53.71**	**128.29**	4.30	**75.58**	21.33	0	5.64	0	0
**SF-268**	0	0.73	**51.50**	21.80	**62.03**	3.63	**80.07**	20.31	13.65			
**SF-295**	13.18	1.06	**78.32**	**68.72**	**149.27**	1.19	**138.85**	**55.97**	0	**56.38**	1.33	1.04
**Continued**								56.23	1.95	12.11	4.31	0.56
**SF-539**	9.57	5.22	**58.34**	12.04	**62.90**	5.73	**57.39**			29.47	12.25	3.69
**SNB-19**	0	0	**47.95**	**39.59**	**95.65**	7.76	**75.84**	28.97	0	20.22	2.37	0
**SNB-75**	10.65	0.61	**30.71**	2.77	1.60	12.39	8.15	13.37	0	9.91	7.77	3.91
**U251**	4.35	0	**77.67**	**98.38**	**115.25**	**41.02**	**92.04**	**46.76**	0	**43.95**	2.36	0
**Melanoma**												
**LOX IMVI**	7.23	3.70	**100.91**	**65.96**	**155.86**	18.88	**99.12**	**49.36**	11.95	33.15	12.38	0
**MALME-3M**	1.28	12.86	**92.21**	**88.22**	**158.65**	9.55	**93.47**	**71.57**	21.36	19.90	1.54	2.41
**M14**	8.89	3.58	**74.09**	**39.75**	**80.33**	17.14	**73.78**	**57.97**	0.44	15.66	1.67	0
**MDA-MB-435**	0	0	**52.42**	6.12	**61.19**	0	**31.49**	**35.98**	0	1.11	0.91	0
**SK-MEL-2**	0	0	**33.28**	16.72	**61.09**	2.90	**35.90**	16.41	0	13.30	0	0
**SK-MEL-28**	0	0	**54.65**	28.10	**109.23**	0	**87.53**	**53.48**	0	20.39	0	0
**SK-MEL-5**	4.85	5.02	**97.42**	11.02	**63.84**	2.92	**66.34**	**58.08**	4.32	7.75	9.57	2.04
**UACC-257**	0	0	**74.92**	0	**68.26**	6.59	**45.62**	**45.65**	0	0	0	0
**UACC-62**	0	1.06	**56.35**	11.85	**83.15**	0	**69.11**	**71.28**	5.20	26.16	1.88	1.04
**IGROV1**	4.80	14.96	**80.88**	**59.83**	**127.35**	**31.01**	**85.41**	**31.57**	17.03			
**OVCAR-3**	0	0	**88.68**	29.34	**152.88**	0	**79.87**	**38.44**	0	26.51	14.29	0.16
**OVCAR-4**	0	0	**76.99**	**63.60**	**117.88**	0	**130.63**	0	0	**32.23**	0	0
**OVCAR-5**	0	0	**46.76**	10.84	**76.28**	0	22.66	0	0	25.74	5.13	0.60
**OVCAR-8**	1.37	5.81	**78.40**	**48.73**	**85.77**	17.10	**68.70**	**39.48**	0	11.87	3.26	0
**NCI/ADR-RES**	0	0.43	22.73	22.98	**58.78**	0	26.43	0	0	22.28	6.74	0
**SK-OV-3**	4.70	0	**39.50**	1.74	**50.18**	7.23	27.70	12.22	5.09	0	1.07	0
**Renal Cancer**										21.76	0.37	0
**786-0**	11.58	0	**50.97**	**87.83**	**142.12**	0	**96.85**	**48.80**	0			
**A498**	22.08	12.86	**100.50**	27.11	**43.85**	36.67	**33.43**	**101.95**	5.34	0	0	0
**ACHN**	3.26	1.36	**67.71**	**64.33**	**120.18**	9.01	**78.11**	**32.27**	4.33	22.48	**57.69**	**56.86**
**CAKI-1**	1.57	4.40	**57.25**	15.06	**38.77**	9.61	**39.51**	24.00	1.91	0.62	6.31	0
**RXF 393**	15.54	7.79	**68.40**	22.63	**81.08**	12.49	**56.23**	**63.67**	0	10.39	17.05	7.78
**SN12C**	3.99	1.47	**80.35**	**58.81**	**136.90**	1.23	**93.76**	**31.47**	0	7.62	7.61	13.89
**TK-10**	0	0	10.16	5.63	**104.33**	0	**60.87**	22.64	0	0	10.09	0
**UO-31**	8.73	13.31	**57.44**	24.61	**78.42**	27.84	18.07	11.66	15.86	0	0	0
**Prostate Cancer**										25.13	6.39	16.76
**PC-3**	0	4.81	**78.57**	**53.21**	**124.90**	7.03	**104.40**	**40.06**	0			
**DU-145**	3.83	0	**57.97**	14.62	**69.46**	0	20.74	2.57	0	18.38	7.83	7.76
**Breast Cancer**										5.98	0	0
**MCF7**	23.51	23.84	**99.28**	**82.56**	**119.97**	13.41	**83.33**	**87.88**	25.12			
**MDA-MB 231**	0	15.58	23.15	**36.06**	**90.52**	11.40	**60.16**	0	2.75	11.50	22.10	14.58
**HS 578T**	7.92	6.32	**63.24**	30.02	**67.05**	10.43	**67.67**	**51.70**	5.94	27.19	20.49	2.36
**BT-549**	4.12	0	**85.96**	0	0	0	**43.07**	29.28	0	16.99	9.75	1.05
**T-47D**	18.22	14.13	**89.50**	**40.63**	**113.10**	12.30	**76.71**	**84.11**	24.20	15.72	0	7.91
**MDA-MB-468**	0	0	**138.86**	23.87	**144.13**	0	**75.82**	0	0	19.77	18.43	0.30
										**90.44**	0	0

**Table 2:** Screening results of in vitro five doses in µM for compound **4c**.

Panel Name	Cell Name	GI_50_			TGI ^b^	LC_50_
		Conc. per Cell Line	Subpanel MID ^b^	Selectivity Ratio (MID ^a^/MID ^b^)		
**Leukemia**	**CCRF-CEM**	0.591	0.55	5.96	17.70	>100
	**HL-60(TB)**	0.797			5.73	>100
	**K-562**	0.499			8.44	54.60
	**MOLT-4**	0.421			3.85	>100
	**RPMI-8226**	0.461			2.41	>100
	**SR**	0.566			4.87	>100
**Non-Small Cell Lung Cancer**	**A549/ATCC**	3.98	3.52	0.93	24.10	>100
	**EKVX**	3.19			21.20	>100
	**HOP-62**	6.42			30.80	>100
	**HOP-92**	0.985			7.52	47.60
	**NCI-H226**	5.03			31.20	>100
	**NCI-H23**	1.85			11.50	67.60
	**NCI-H322M**	5.04			26.40	>100
	**NCI-H460**	2.10			4.49	9.61
	**NCI-H522**	3.05			11.70	>100
**Colon Cancer**	**COLO 205**	1.46	2.53	1.30	3.76	-
	**HCC-2998**	1.48			4.81	28.10
	**HCT-116**	2.25			6.90	82.30
	**HCT-15**	2.60			13.30	>100
	**HT29**	3.02			>100	>100
	**KM12**	3.43			13.00	82.60
	**SW-620**	3.44			10.80	55.60
**CNS Cancer**	**SF-268**	4.97	4.05	0.81	24.30	>100
	**SF-295**	3.40			13.10	43.80
	**SF-539**	2.73			10.10	33.80
	**SNB-19**	7.48			32.40	>100
	**SNB-75**	2.78			18.00	>100
	**U251**	2.92			10.80	48.00
**Melanoma**	**LOX IMVI**	1.34	2.87	1.14	3.22	7.74
	**MALME-3M**	1.48			5.00	23.60
	**M14**	2.73			9.13	67.10
	**MDA-MB-435**	3.57			12.20	47.60
	**SK-MEL-2**	5.10			21.90	81.30
	**SK-MEL-28**	2.60			10.30	39.00
	**SK-MEL-5**	2.11			4.78	12.80
	**UACC-257**	3.37			15.40	>100
	**UACC-62**	3.51			14.90	52.20
**Ovarian Cancer**	**IGROV1**	3.04	5.77	0.57	11.40	52.40
	**OVCAR-3**	3.25			8.23	40.90
	**OVCAR-4**	2.75			8.64	>100
	**OVCAR-5**	4.43			37.80	>100
	**OVCAR-8**	2.89			13.70	>100
	**NCI/ADR-RES**	12.60			>100	>100
	**SK-OV-3**	11.40			39.60	>100
**Renal Cancer**	**786-0**	3.92	4.30	0.76	15.70	58.20
	**A498**	2.40			7.75	36.40
	**ACHN**	4.36			31.90	>100
	**CAKI-1**	3.49			16.10	85.40
	**RXF 393**	2.03			7.07	34.00
	**SN12C**	3.14			18.10	>100
	**TK-10**	8.99			40.23	>100
	**UO-31**	6.05			38.30	>100
**Prostate Cancer**	**PC-3**	0.420	2.20	1.49	15.30	>100
	**DU-145**	3.99			21.40	>100
**Breast Cancer**	**MCF7**	0.582	2.50	1.31	2.85	13.30
	**MDA-MB-231**	3.75			13.90	69.10
	**HS 578T**	3.58			24.40	>100
	**BT-549**	2.84			9.70	50.50
	**T-47D**	2.29			7.21	>100
	**MDA-MB-468**	1.97			4.79	17.70
**MID ^a^**	**3.28**					

MID ^a^: Average sensitivity of all cell lines in µM. MID ^b^: Average sensitivity of all subpanel cell lines in µM.

**Table 3:** Screening results of in vitro five doses in µM for compound **4e**.

Panel Name	Cell Name	GI_50_			TGI ^b^	LC_50_
		Conc. per Cell Line	Subpanel MID ^b^	Selectivity Ratio (MID ^a^/MID ^b^)		
**Leukemia**	**CCRF-CEM**	1.43	1.33	1.35	9.03	65.80
	**HL-60(TB)**	1.85			6.53	54.80
	**K-562**	0.969			3.23	12.30
	**MOLT-4**	1.55			4.81	28.20
	**RPMI-8226**	1.16			3.52	16.50
	**SR**	1.01			3.95	37.00
**Non-Small Cell Lung Cancer**	**A549/ATCC**	2.16	1.9	0.94	4.53	9.52
	**EKVX**	1.82			3.68	7.43
	**HOP-62**	1.80			4.10	9.32
	**HOP-92**	1.67			3.53	7.47
	**NCI-H226**	1.77			3.48	6.86
	**NCI-H23**	1.93			4.43	10.80
	**NCI-H322M**	2.15			5.26	19.80
	**NCI-H460**	1.56			3.13	6.30
	**NCI-H522**	2.24			5.85	77.90
**Colon Cancer**	**COLO 205**	1.55	1.72	1.04	3.25	8.82
	**HCC-2998**	1.82			3.44	6.50
	**HCT-116**	1.45			3.51	8.52
	**HCT-15**	1.42			3.03	6.46
	**HT29**	1.97			5.47	44.10
	**KM12**	2.03			4.46	9.80
	**SW-620**	1.83			3.69	7.43
**CNS Cancer**	**SF-268**	2.08	1.94	0.92	4.35	9.09
	**SF-295**	1.86			3.50	6.56
	**SF-539**	1.70			3.17	5.93
	**SNB-19**	2.34			5.06	13.30
	**SNB-75**	1.59			3.62	8.22
	**U251**	2.10			4.18	8.35
**Melanoma**	**LOX IMVI**	1.57	1.77	1.01	2.94	5.50
	**MALME-3M**	1.55			3.15	6.41
	**M14**	1.65			3.84	8.96
	**MDA-MB-435**	1.73			3.35	6.49
	**SK-MEL-2**	2.09			4.64	11.40
	**SK-MEL-28**	1.58			3.04	5.86
	**SK-MEL-5**	1.81			3.39	6.33
	**UACC-257**	2.14			4.72	11.60
	**UACC-62**	1.78			3.40	6.49
**Ovarian Cancer**	**IGROV1**	1.50	1.81	0.99	2.98	5.92
	**OVCAR-3**	1.84			3.39	6.28
	**OVCAR-4**	1.74			3.29	6.23
	**OVCAR-5**	1.85			3.97	8.52
	**OVCAR-8**	2.11			4.74	16.50
	**NCI/ADR-RES**	1.83			4.43	12.70
	**SK-OV-3**	1.79			4.31	11.00
**Renal Cancer**	**786-0**	1.79	1.77	1.01	3.48	6.76
	**A498**	1.74			4.94	19.60
	**ACHN**	1.73			3.17	5.78
	**CAKI-1**	1.70			3.29	6.37
	**RXF 393**	1.61			3.27	6.64
	**SN12C**	1.96			4.26	9.26
	**TK-10**	2.06			4.30	8.98
	**UO-31**	1.57			2.99	5.67
**Prostate Cancer**	**PC-3**	1.55	1.65	1.08	3.30	7.05
	**DU-145**	1.76			3.44	6.70
**Breast Cancer**	**MCF7**	1.15	2.27	0.79	2.56	5.73
	**MDA-MB-231**	1.85			3.77	7.67
	**HS 578T**	2.28			5.27	63.20
	**BT-549**	4.93			2.13	75.80
	**T-47D**	1.79			3.83	8.21
	**MDA-MB-468**	1.64			3.23	6.36
**MID ^a^**	**1.79**					

MID ^a^: Average sensitivity of all cell lines in µM. MID ^b^: Average sensitivity of all subpanel cell lines in µM.

**Table 4:** Screening results of five doses in µm for compound **4g**.

Panel Name	Cell Name	GI_50_			TGI ^b^	LC_50_
		Conc. per Cell Line	Subpanel MID ^b^	Selectivity Ratio (MID ^a^/MID ^b^)		
**Leukemia**	**CCRF-CEM**	4.90	2.88	0.87	>100	>100
	**HL-60(TB)**	1.30			>100	>100
	**K-562**	2.69			11.20	>100
	**MOLT-4**	4.53			30.40	>100
	**RPMI-8226**	0.997			4.77	>100
	**SR**	2.87			15.40	>100
**Non-Small Cell Lung Cancer**	**A549/ATCC**	2.46	2.71	0.93	5.65	48.10
	**EKVX**	2.11			5.84	36.60
	**HOP-62**	1.88			6.37	53.60
	**HOP-92**	1.52			4.11	15.10
	**NCI-H226**	1.79			4.43	13.50
	**NCI-H23**	2.74			11.20	75.90
	**NCI-H322M**	2.49			7.20	55.40
	**NCI-H460**	1.83			3.59	7.05
	**NCI-H522**	7.60			81.50	>100
**Colon Cancer**	**COLO 205**	1.94	2.63	0.96	5.43	33.00
	**HCC-2998**	2.57			6.44	37.80
	**HCT-116**	2.14			5.12	18.90
	**HCT-15**	3.24			12.80	82.00
	**HT29**	3.09			22.10	>100
	**KM12**	3.03			9.58	>100
	**SW-620**	2.41			5.58	31.00
**CNS Cancer**	**SF-268**	2.09	2.19	1.15	5.10	20.80
	**SF-295**	2.23			5.05	15.00
	**SF-539**	1.73			3.46	6.92
	**SNB-19**	3.13			7.95	49.60
	**SNB-75**	1.53			4.57	25.70
	**U251**	2.43			5.22	30.40
**Melanoma**	**LOX IMVI**	2.19	2.43	1.04	4.01	7.34
	**MALME-3M**	1.67			3.63	7.90
	**M14**	2.41			7.76	40.50
	**MDA-MB-435**	3.11			9.84	45.50
	**SK-MEL-2**	3.59			13.80	69.70
	**SK-MEL-28**	1.79			3.37	6.35
	**SK-MEL-5**	1.89			3.88	7.95
	**UACC-257**	3.27			10.10	60.00
	**UACC-62**	1.94			4.47	10.90
**Ovarian Cancer**	**IGROV1**	1.65	3.05	0.83	3.97	9.57
	**OVCAR-3**	2.28			5.14	16.00
	**OVCAR-4**	1.79			3.36	6.33
	**OVCAR-5**	2.35			6.89	33.50
	**OVCAR-8**	3.19			11.50	>100
	**NCI/ADR-RES**	7.81			>100	>100
	**SK-OV-3**	2.30			12.80	59.10
**Renal Cancer**	**786-0**	1.93	2.20	1.14	3.67	6.98
	**A498**	2.45			9.87	54.60
	**ACHN**	2.00			5.04	16.90
	**CAKI-1**	2.10			6.03	25.60
	**RXF 393**	1.86			3.80	7.76
	**SN12C**	2.46			6.79	33.90
	**TK-10**	2.95			6.59	56.70
	**UO-31**	1.86			6.38	25.70
**Prostate Cancer**	**PC-3**	1.65	1.81	1.39	3.76	8.55
	**DU-145**	1.98			4.61	14.00
**Breast Cancer**	**MCF7**	1.47	2.24	1.12	3.84	10.00
	**MDA-MB-231**	2.42			5.45	18.80
	**HS 578T**	2.30			5.64	>100
	**BT-549**	3.36			1.64	>100
	**T-47D**	1.94			4.83	30.00
	**MDA-MB-468**	1.94			4.55	12.60
**MID ^a^**	**2.52**					

MID ^a^: Average sensitivity of all cell lines in µM. MID ^b^: Average sensitivity of all subpanel cell lines in µM.

**Table 5:** IC_50_ values of compounds **4c**, **4d**, **4e**, **4g**, and **4h against** EGFR and BRAF^V600E^.

Compd. No.	EGFR Inhibition IC_50_ ± SEM (µM)	BRAF^V600E^ Inhibition IC_50_ ± SEM (µM)
**4c**	0.11 ± 0.01	0.31 ± 0.07
**4d**	0.68 ± 0.05	0.95 ± 0.07
**4e**	0.09 ± 0.01	0.20 ± 0.02
**4g**	0.27 ± 0.03	0.60 ± 0.05
**4h**	0.56 ± 0.05	0.82 ± 0.06
**Erlotinib**	0.08 ± 0.04	0.06 ± 0.02
**Vemurafenib**	--	0.03 ± 0.01

**Table 6:** Caspase-3, caspase-8, Bax, and Bcl-2 levels for compounds **4c**, **4e**, and staurosporine on prostatic cancer (PC-3) cell line.

Compd. No.	Caspase-3		Caspase-8		Bax		Bcl-2	
	Conc (Pg/mL)	Fold Change	Conc (ng/mL)	Fold Change	Conc (Pg/mL)	Fold Change	Conc (ng/mL)	Fold Reduction
**4c**	365 ± 12	7	1.90	16	185	26	0.80	4
**4e**	470 ± 15	9	2.20	18	220	31	0.75	4
**Staurosporine**	260 ± 5	5	1.65	14	170	24	1.00	3
**Control**	52	1	0.12	1	7	1	3.00	1

**Table 7:** The target compound’s binding energy score throughout the EGFR docking study.

EGFR (PDB: 1 M17)				
Compound	Binding Affinity (kcal mol^−1^)	Distance from the Main Residue (in Å)	Interaction Type	Amino Acids Residue
**4c**	−0.5	3.60	H-donor	MET 742
		3.30	H-acceptor	MET 769
		4.09	pi-H	LEU 694
		4.36	pi-H	LEU 694
**4e**	−1.5	3.35	H-acceptor	MET 769
		3.69	pi-H	LEU 694
Erlotinib	−1.9	3.35	H-acceptor	MET 769
		3.69	pi-H	LEU 694

**Table 8:** The target compounds’ binding energy scores throughout the BRAF^V600^docking study.

BRAF^V600E^ (PDB: 3OG7)				
Compd.	Binding Affinity (kcal mol^−1^)	Distance from the Main Residue (Å)	Interaction Type	Amino Acids Residue
**4c**	−5.4	2.92	H-donor	CYS 532
		3.12	H-acceptor	LYS 483
		3.66	pi-H	LYS 483
**4e**	−1.9	3.07	H-acceptor	CYS 532
		3.52	pi-H	CYS 532
		3.54	pi-H	SER 535
Vemurafenib	−3.5	3.15	H-donor	GLN 530
		3.14	H-acceptor	CYS 532
		3.15	H-acceptor	ASP 594
		3.08	H-acceptor	LYS 483
		2.98	H-acceptor	GLY 596
		3.23	Ionic	LYS 483
		3.08	Ionic	LYS 483
		3.88	Ionic	LYS 483

## Data Availability

The data will be provided upon request.

## Supplementary Materials

The following supporting information can be downloaded at https://www.mdpi.com/article/10.3390/molecules29020446/s1, Figures S1–S47.
