# Supplementary material for: Benzimidazole-Based Derivatives as Apoptotic Antiproliferative Agents: Design, Synthesis, Docking, and Mechanistic Studies
Source: Molecules. 2024 Jan 16;29(2):446. doi: 10.3390/molecules29020446 (PMC10819888; doi:10.3390/molecules29020446)
Supplement: Supplementary file 1 [file molecules-29-00446-s001.zip › molecules-2731927-supplementary.pdf]

Contents

❒ Copies of ^{1}H NMR, ^{13}C NMR and IR spectra of all compounds

Figure S1: ^{1}H- NMR spectrum of compound 1 (400 MHz, DMSO-d_6)

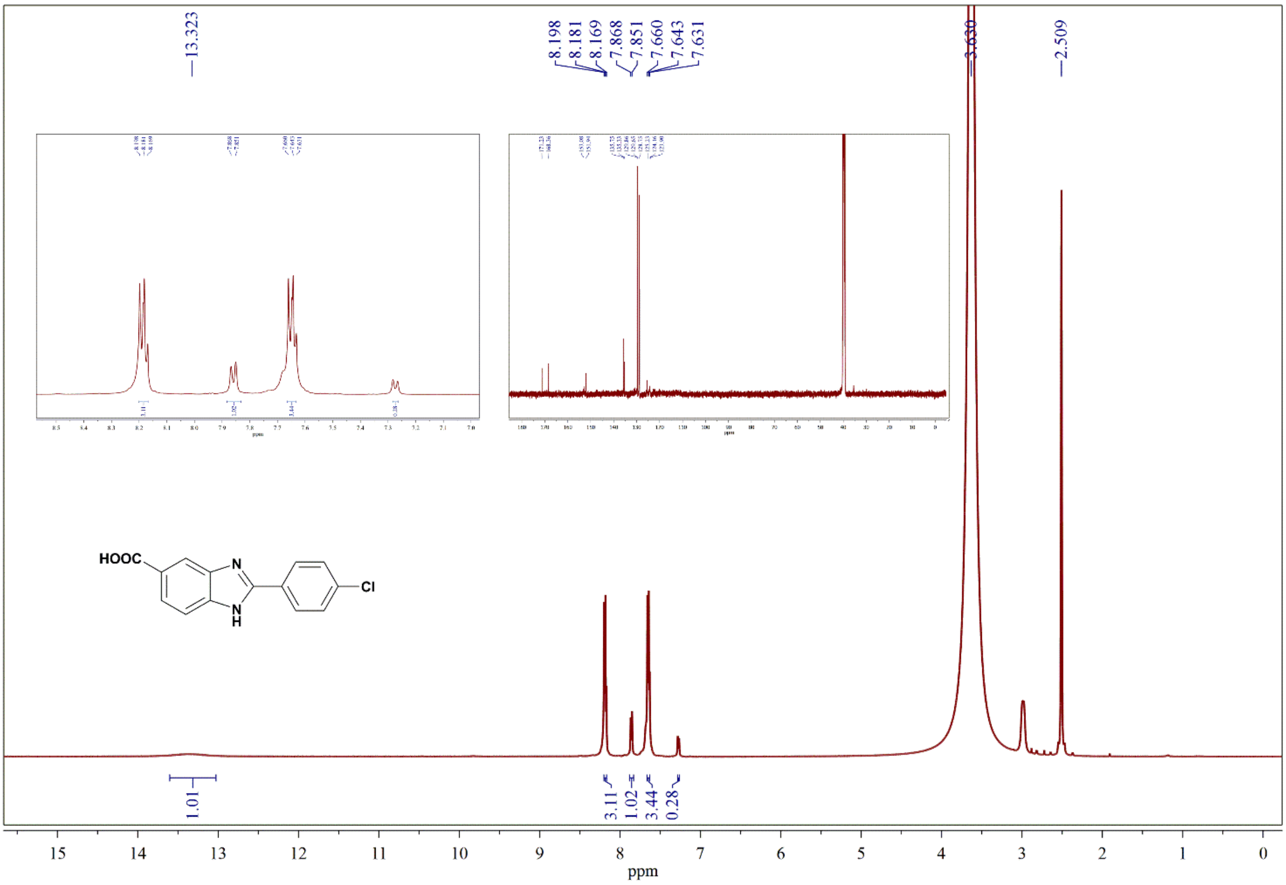

Figure S2: ^{13}C- NMR spectrum of compound 1 (100 MHz, DMSO-d_6)

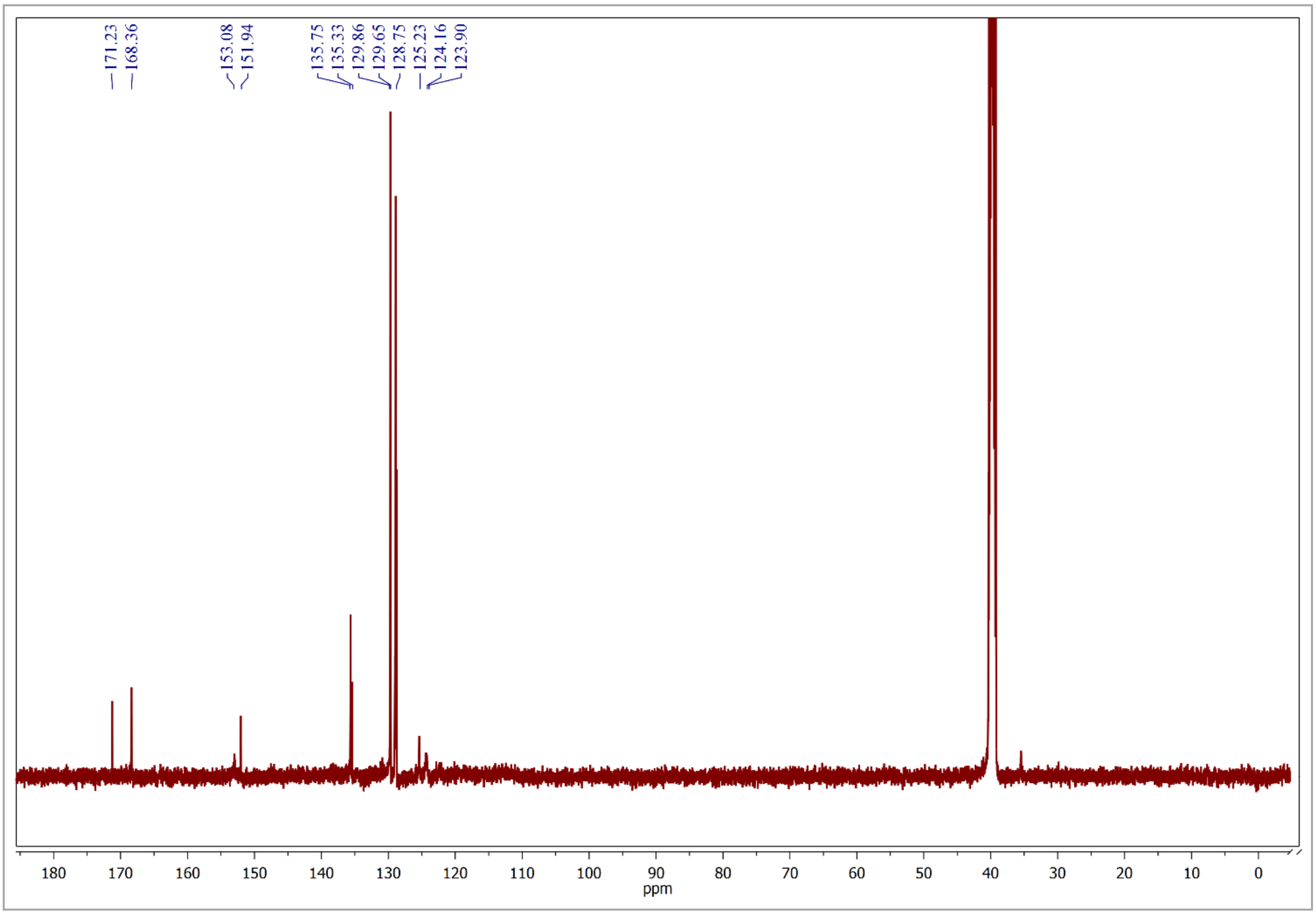

Figure S3: IR spectrum of compound 1 (KBr, υ_{max} cm^{-1})

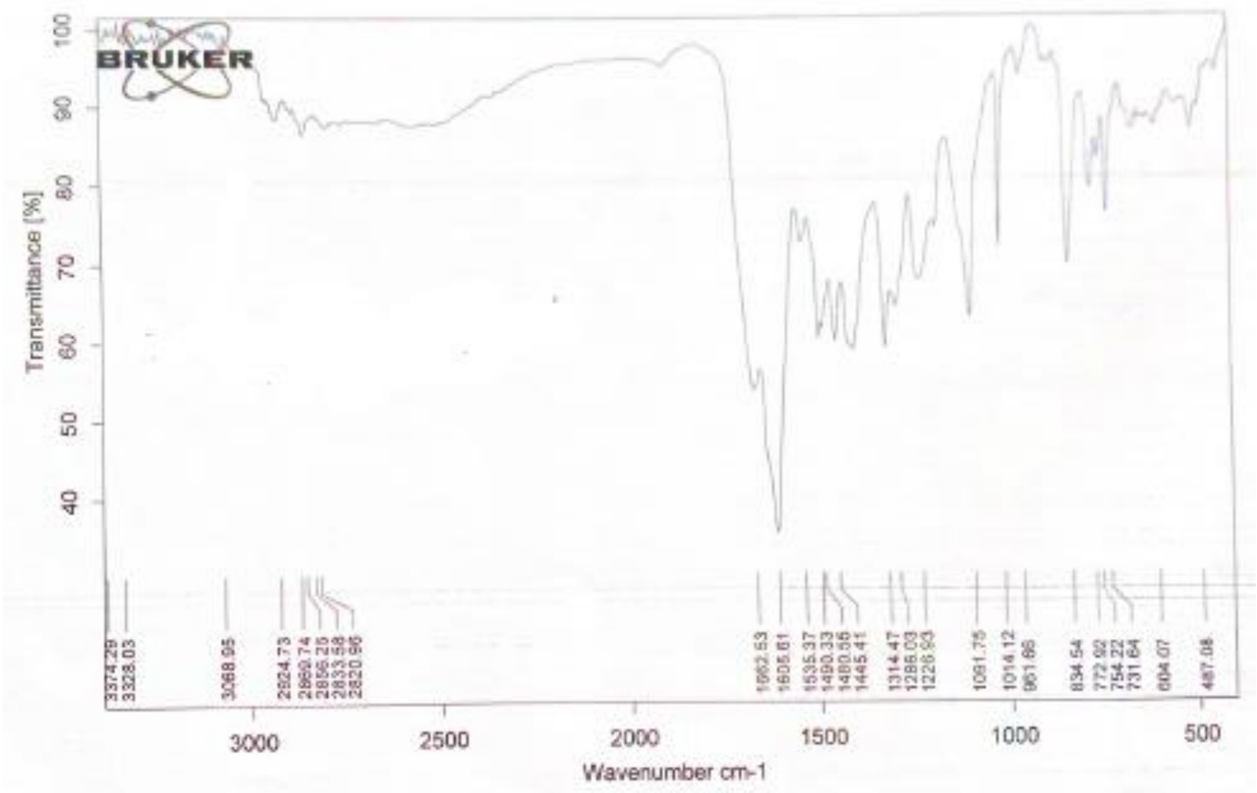

Figure S4: ^{1}H- NMR spectrum of compound 2 (400 MHz, DMSO-*d6*)

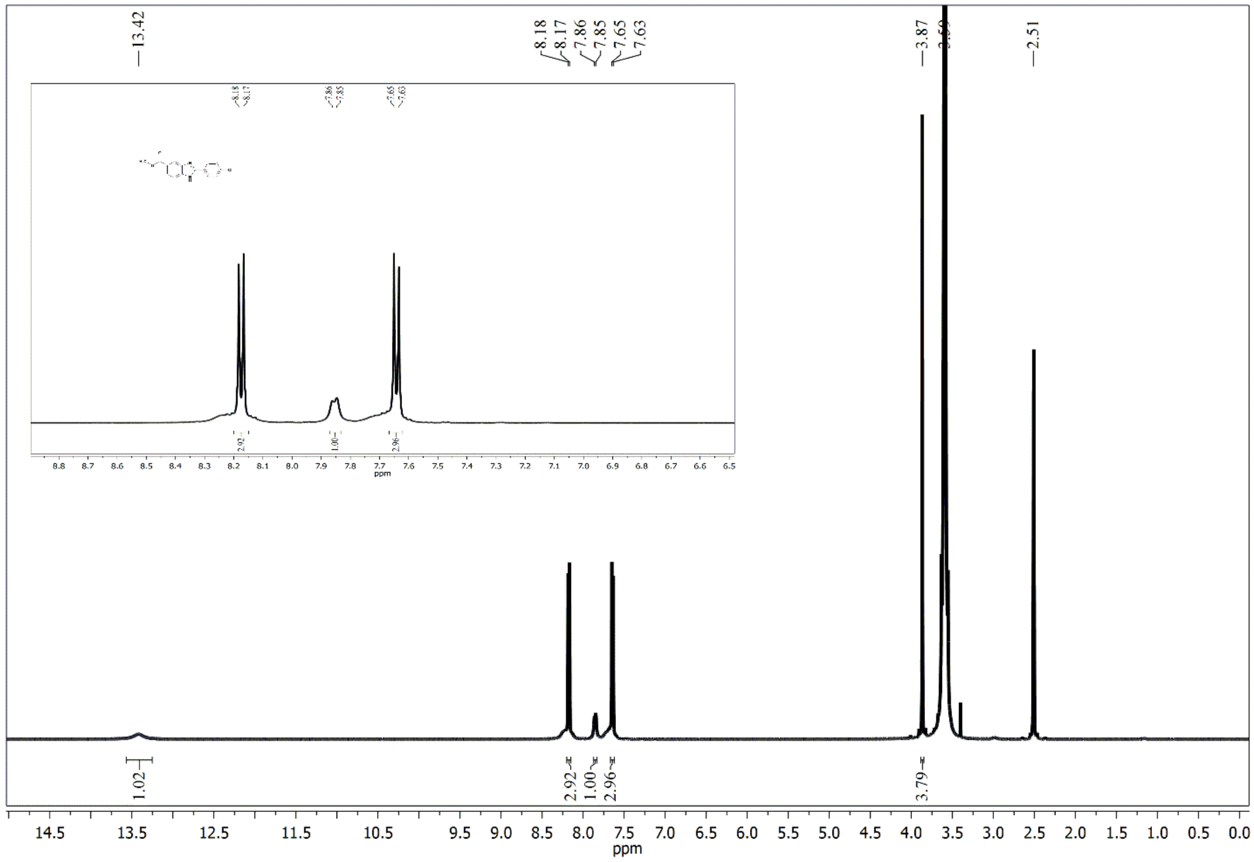

Figure S5: ^{13}C- NMR spectrum of compound 2 (100 MHz, DMSO-*d6*)

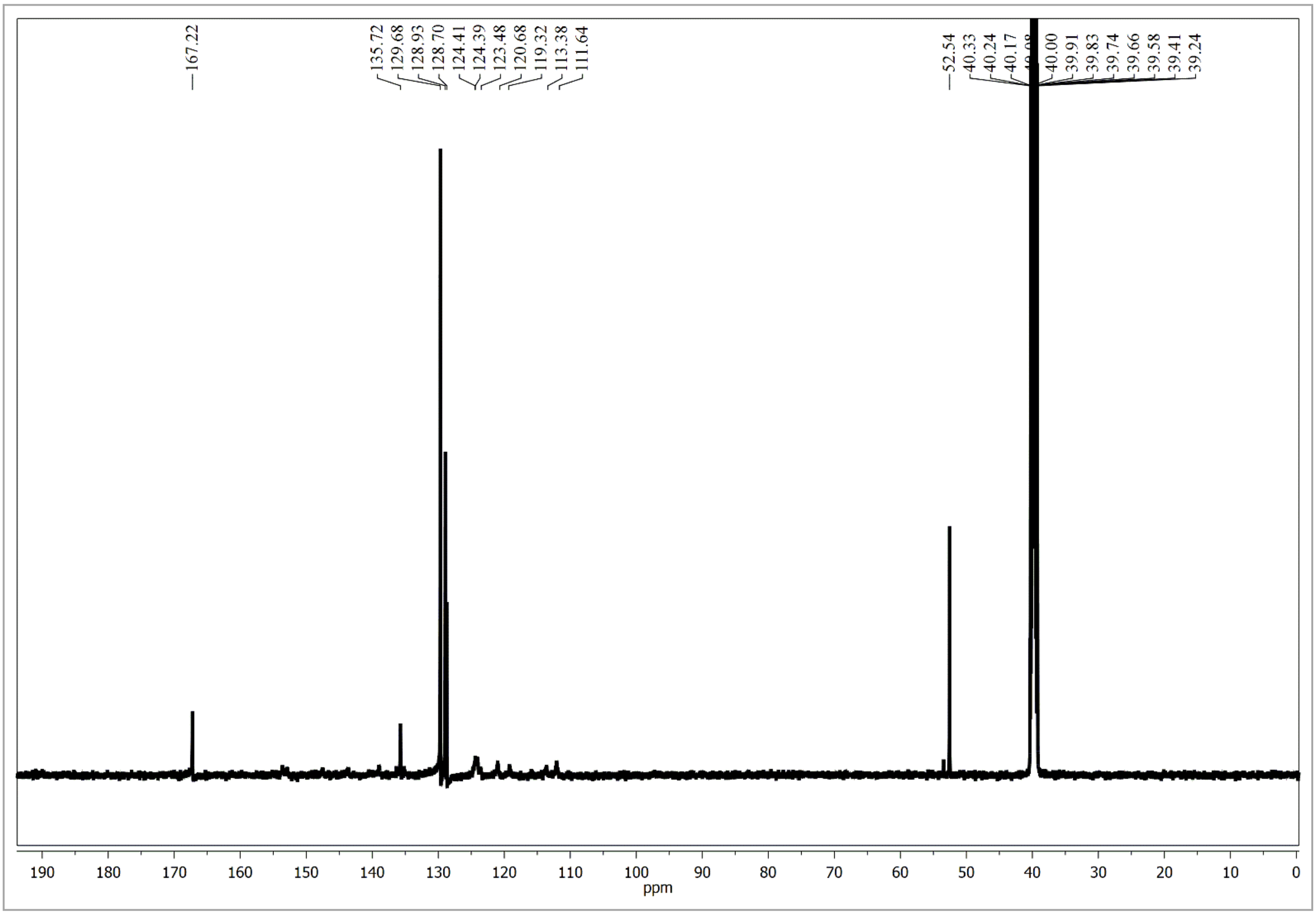

Figure S6: IR spectrum of compound 2 (KBr, υ_{max} cm^{-1})

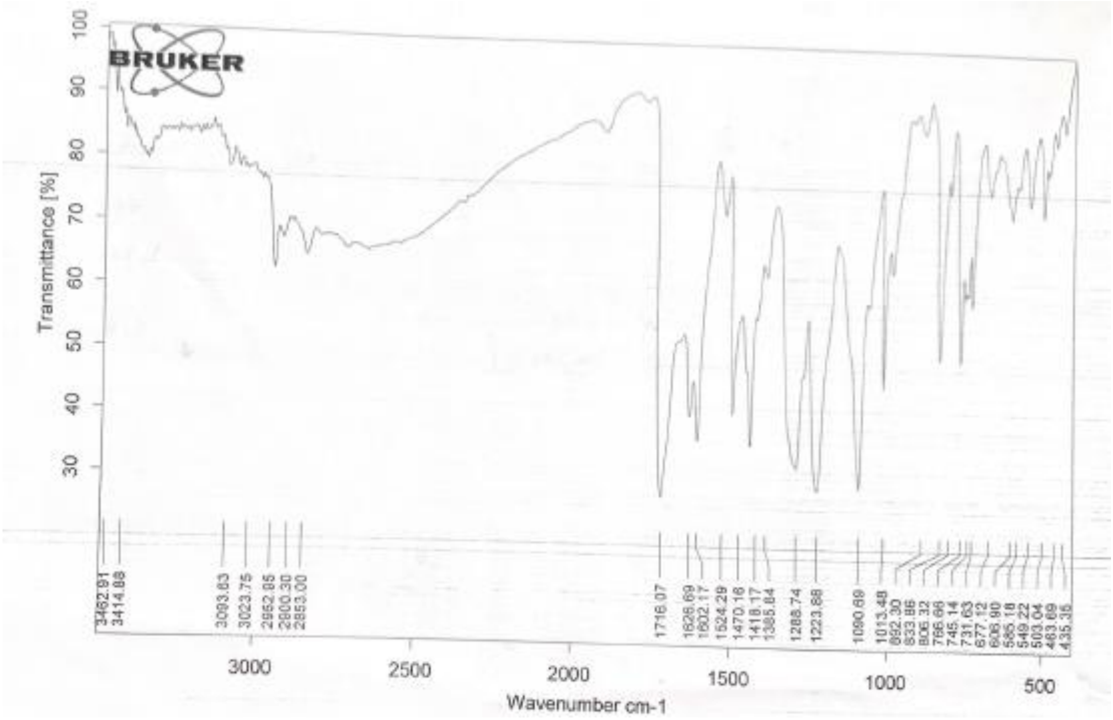

Figure S7: ^{1}H- NMR spectrum of compound <u>3</u> (400 MHz, DMSO-*d6*)

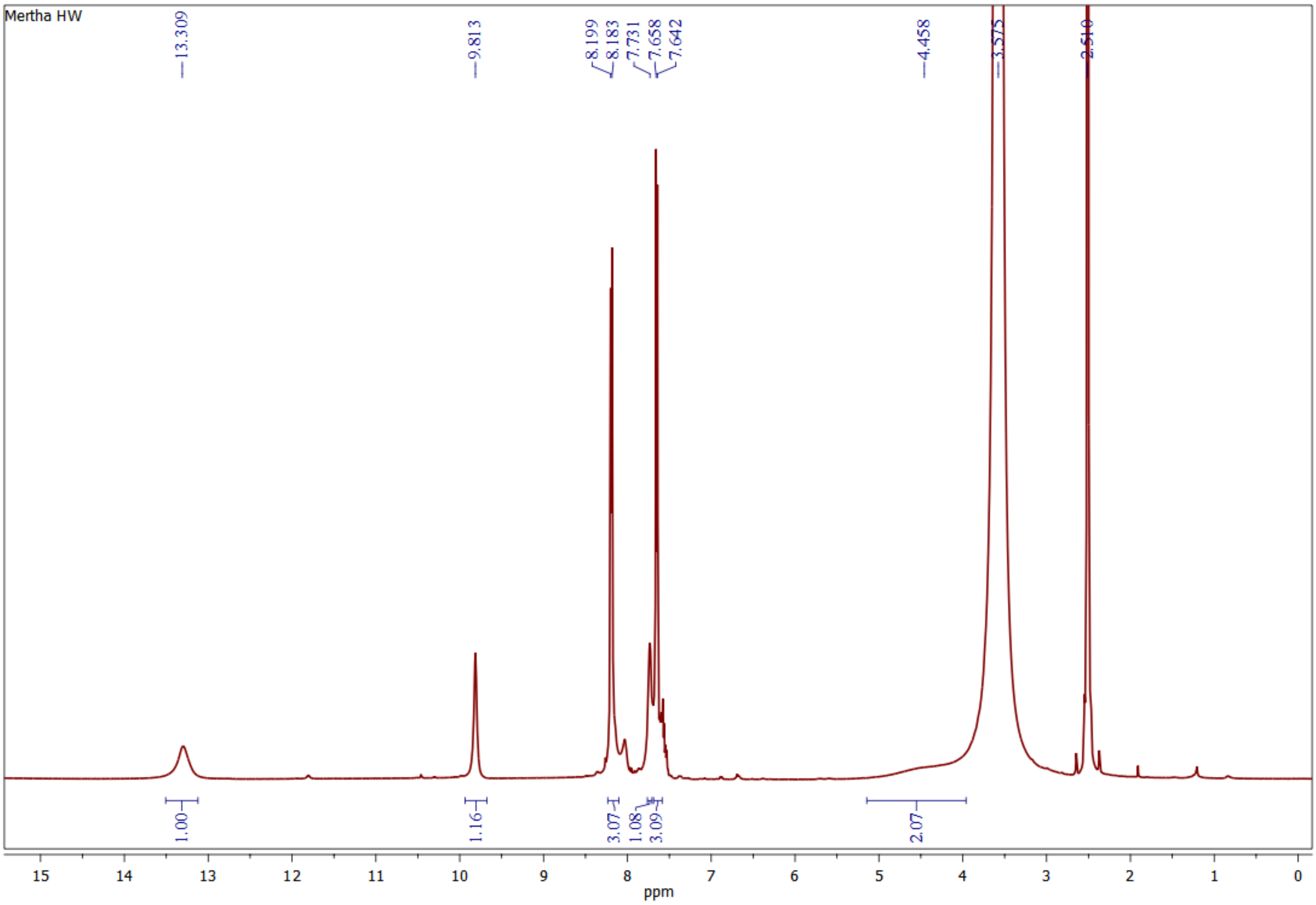

Figure S8: ^{13}C- NMR spectrum of compound 3 (100 MHz, DMSO-*d6*)

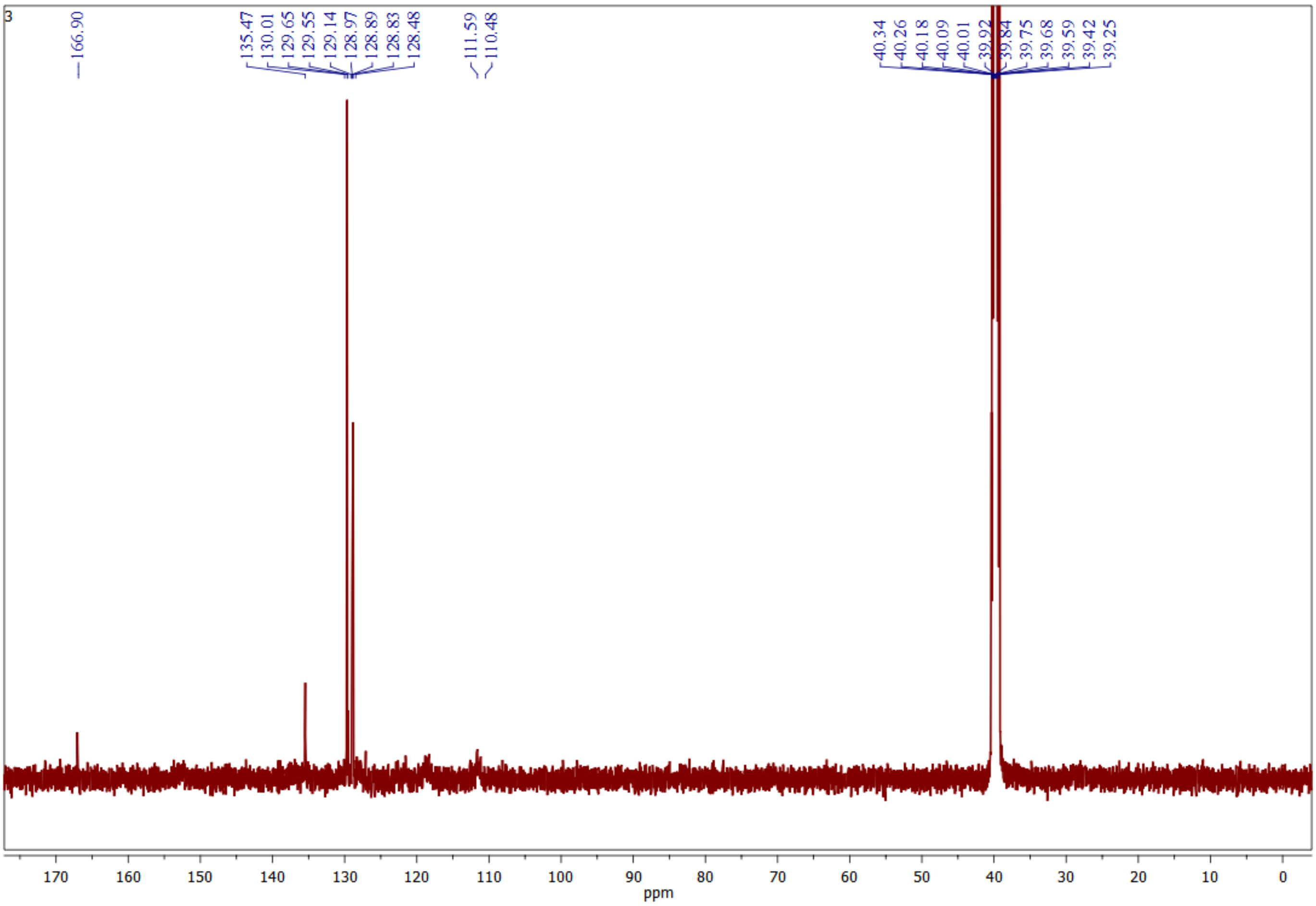

Figure S9: IR spectrum of compound 3 (KBr, υ_{max} cm^{-1})

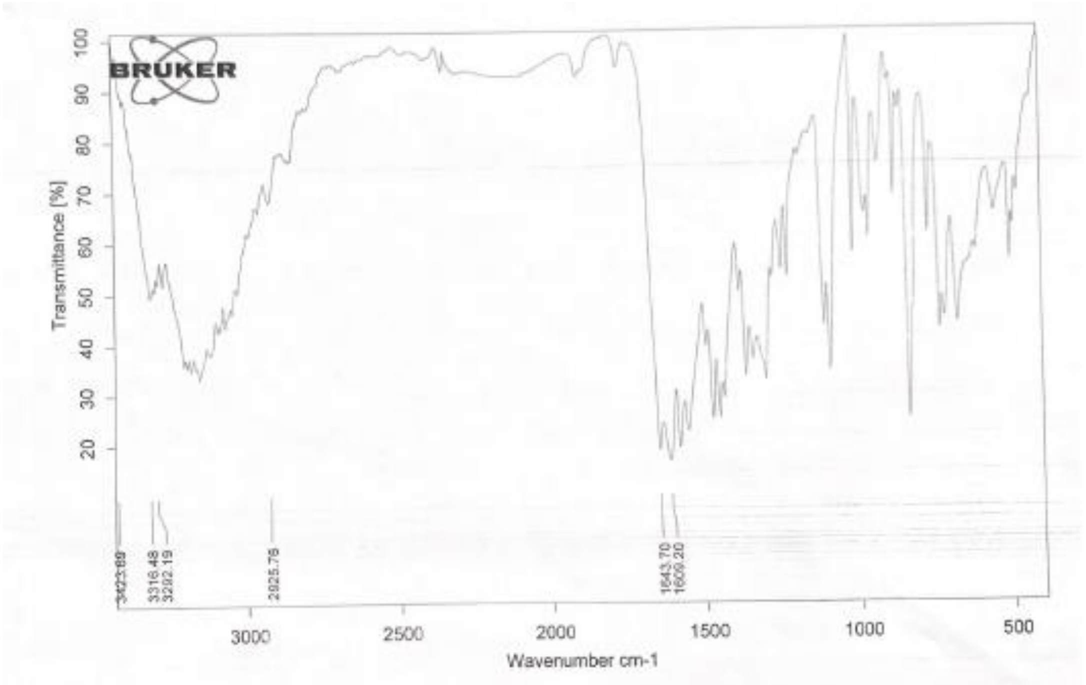

Figure S10: ^{1}H- NMR spectrum of compound 4a (400 MHz, DMSO-*d6*)

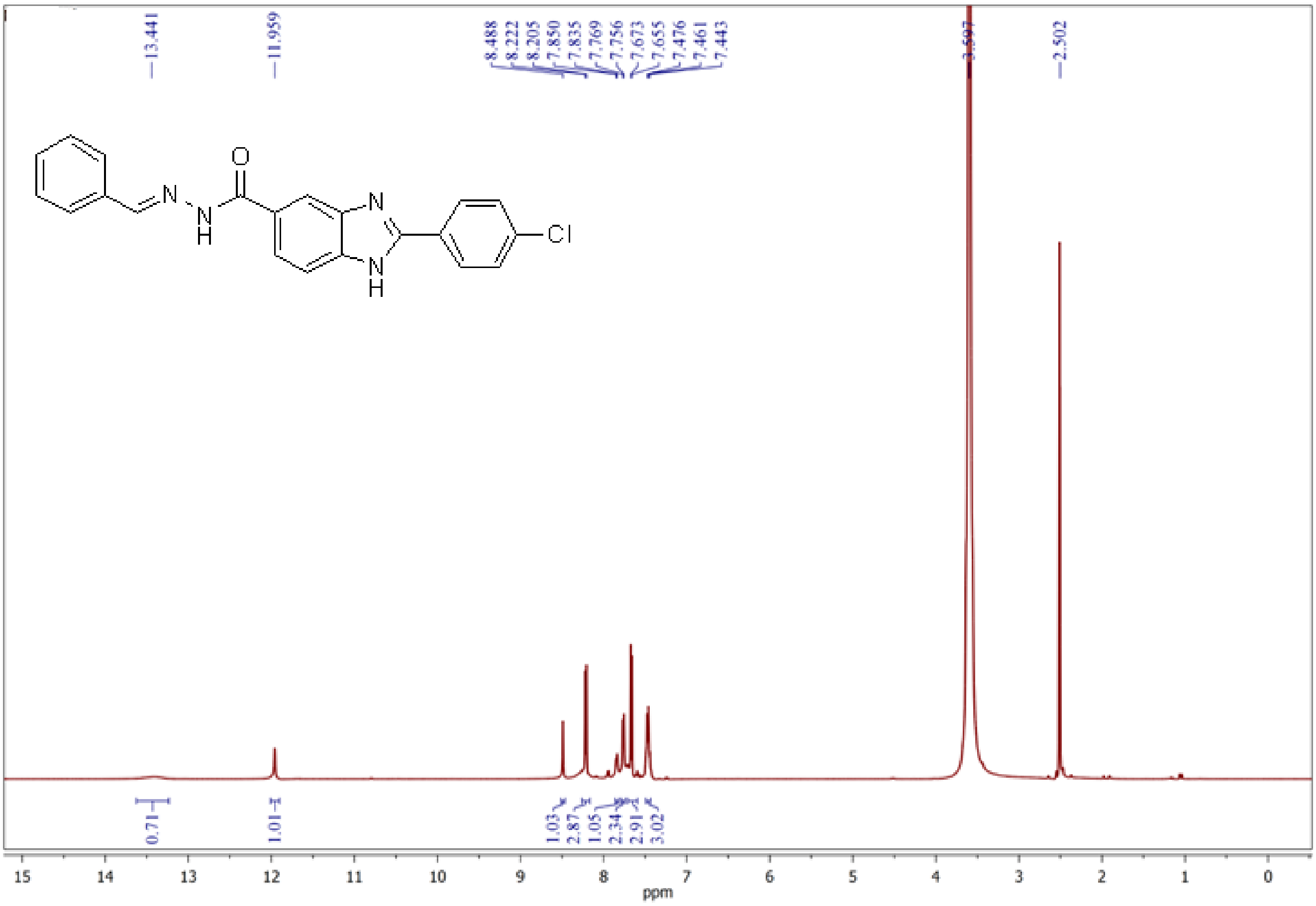

Figure S11: ^{13}C- NMR spectrum of compound 4a (100 MHz, DMSO-*d6*)

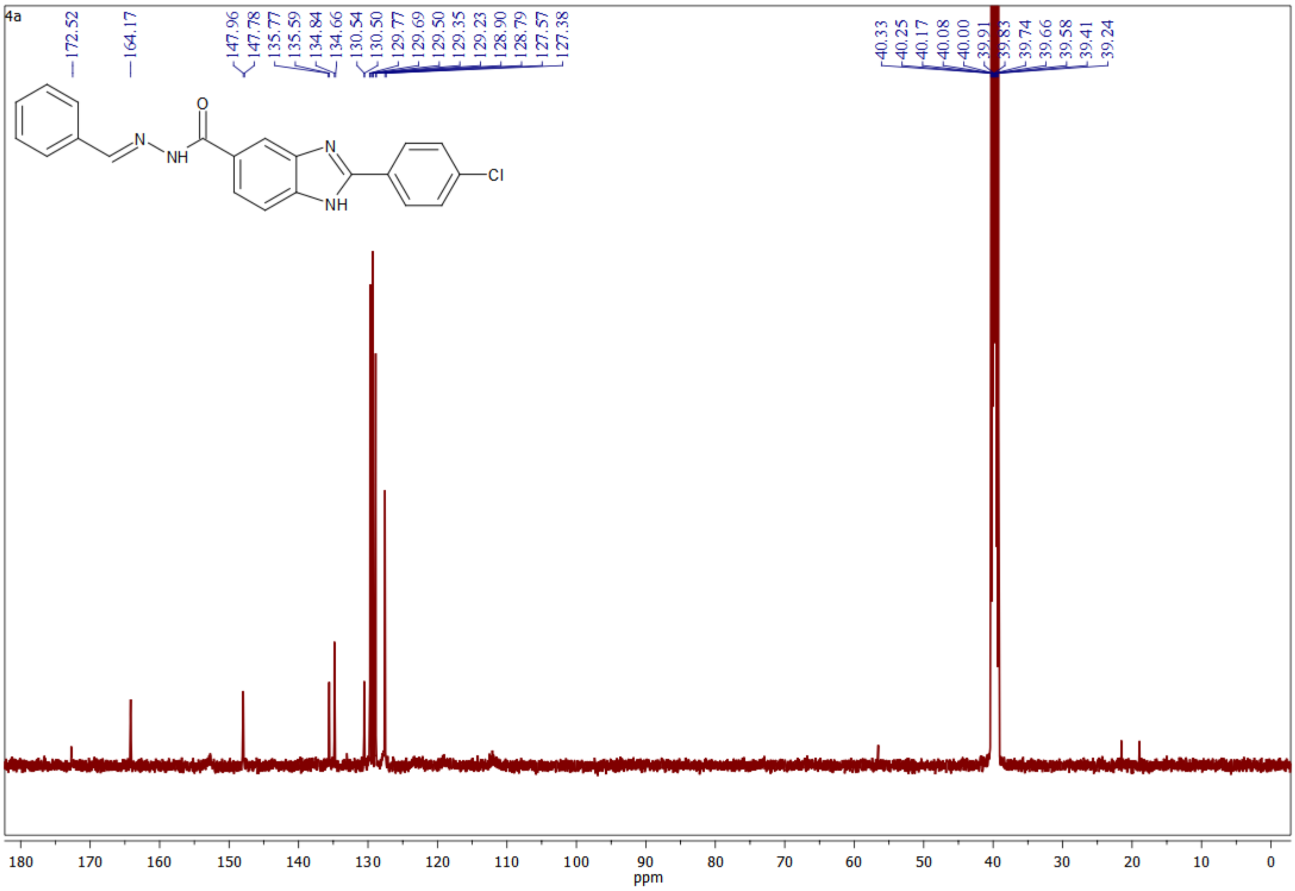

Figure S12: IR spectrum of compound 4a (KBr, υ_{max} cm^{-1})

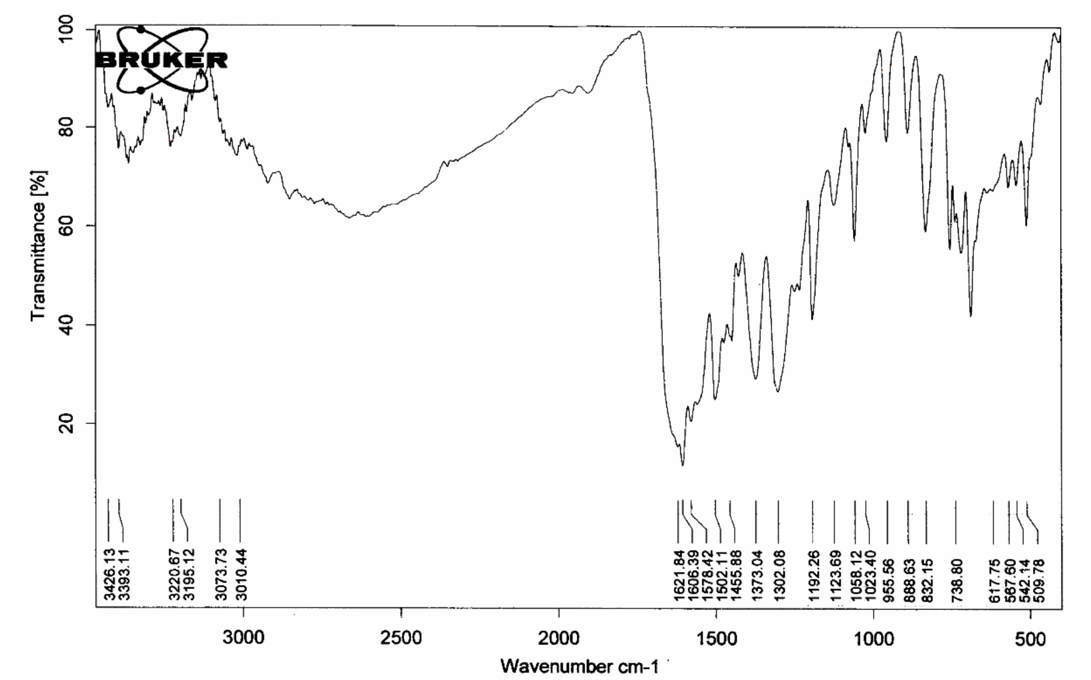

Figure S13: ^{1}H- NMR spectrum of compound 4b (500 MHz, DMSO-*d6*)

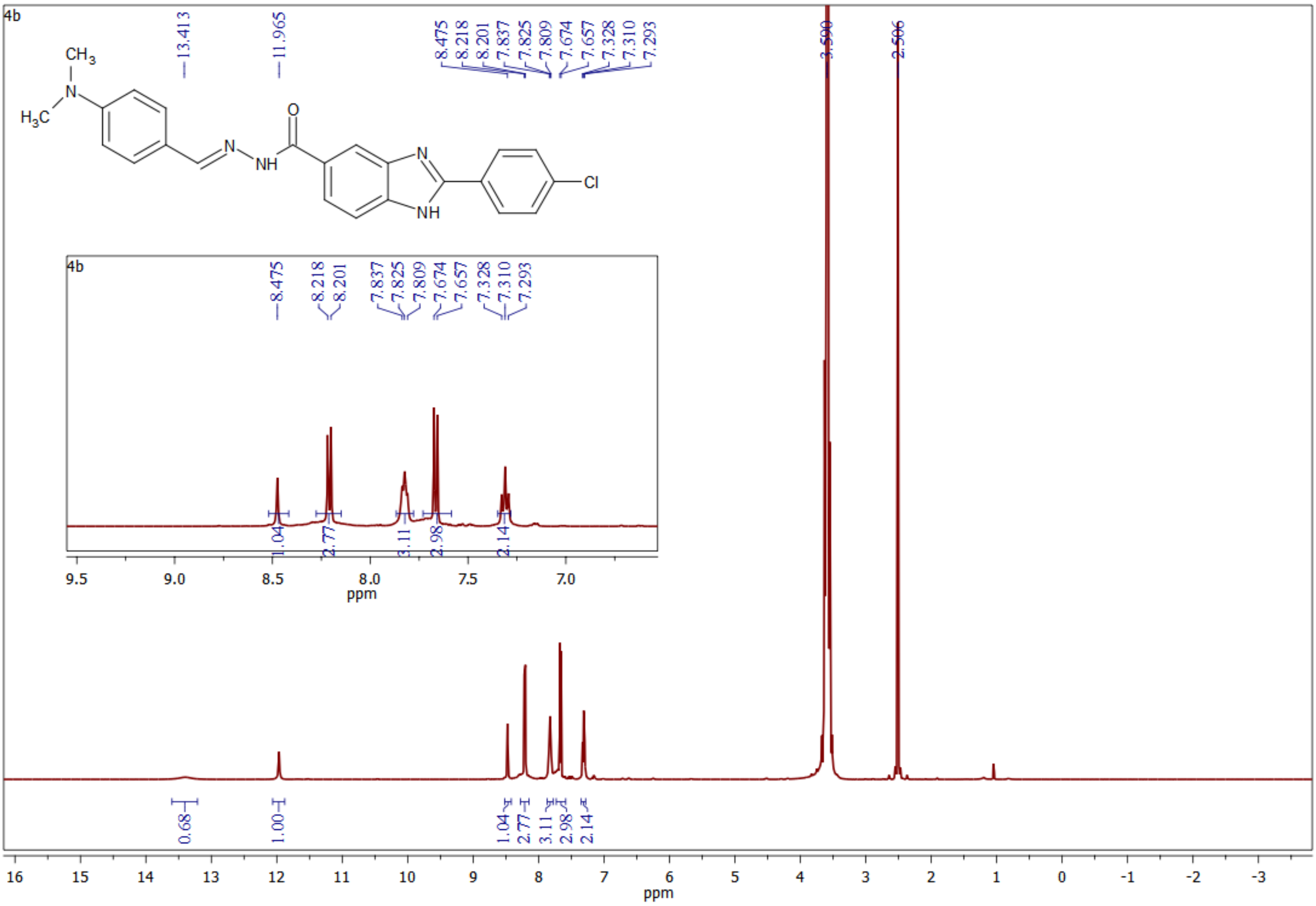

Figure S14: ^{13}C- NMR spectrum of compound 4b (125 MHz, DMSO-*d6*)

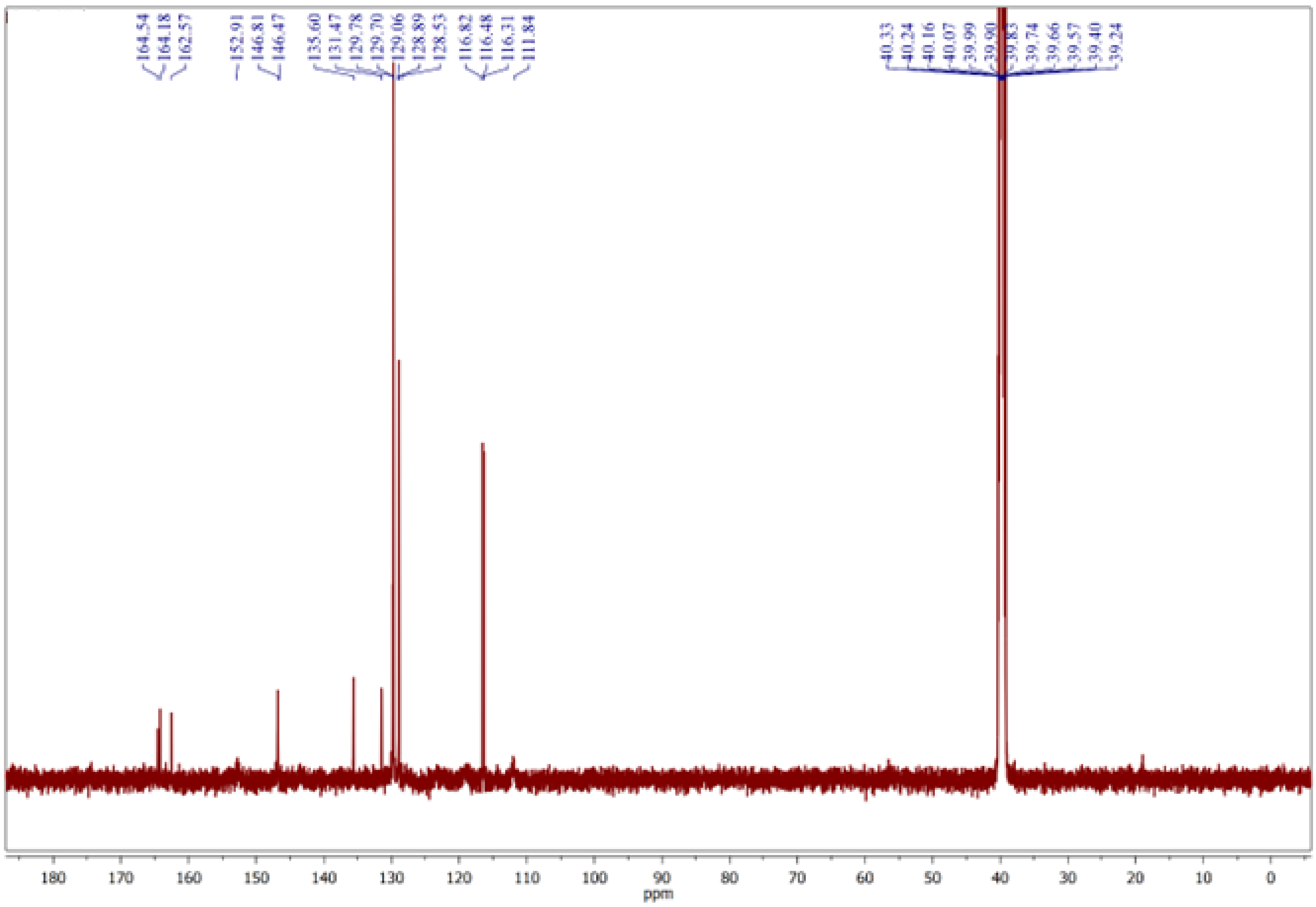

Figure S15: IR spectrum of compound <u>4b</u> (KBr, υ_{max} cm^{-1})
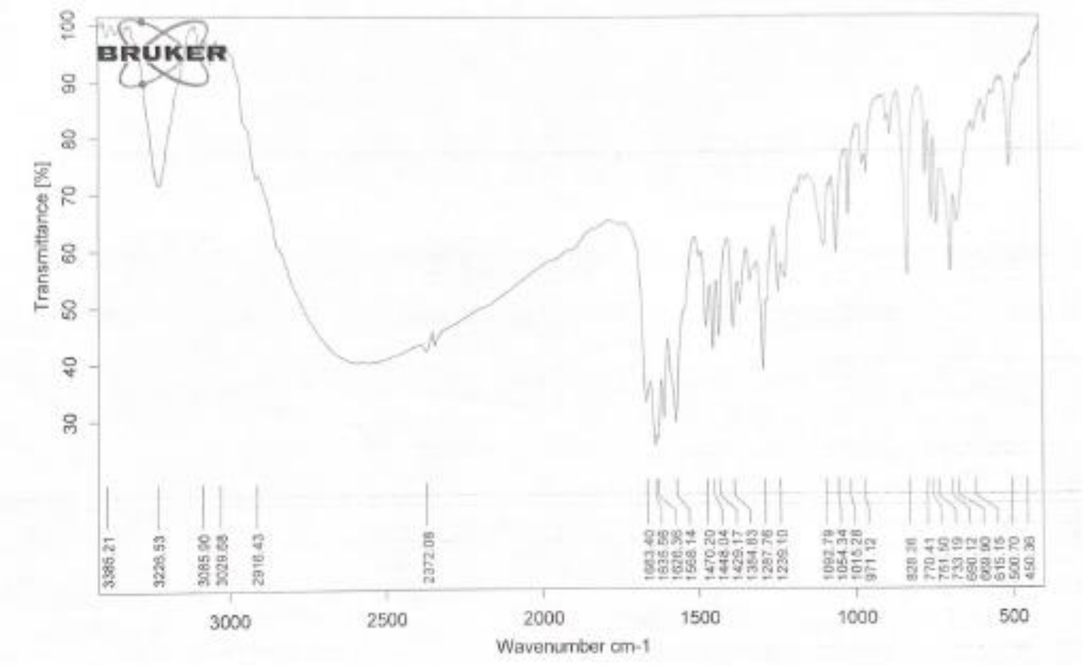

Figure S16: ^{1}H- NMR spectrum of compound 4c (500 MHz, DMSO-*d6*)

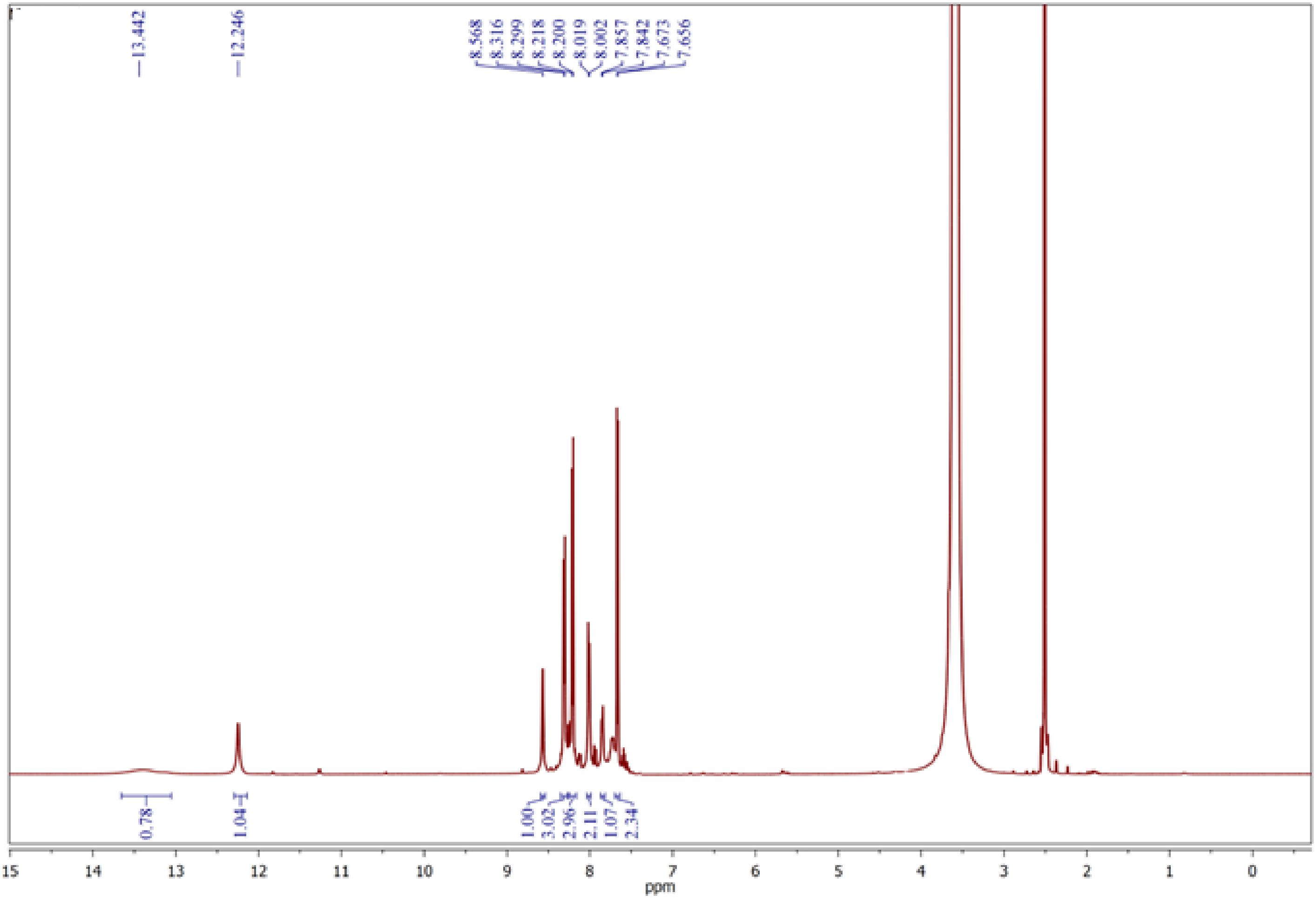

Figure S17: ^{13}C- NMR spectrum of compound 4c (125 MHz, DMSO-*d6*)

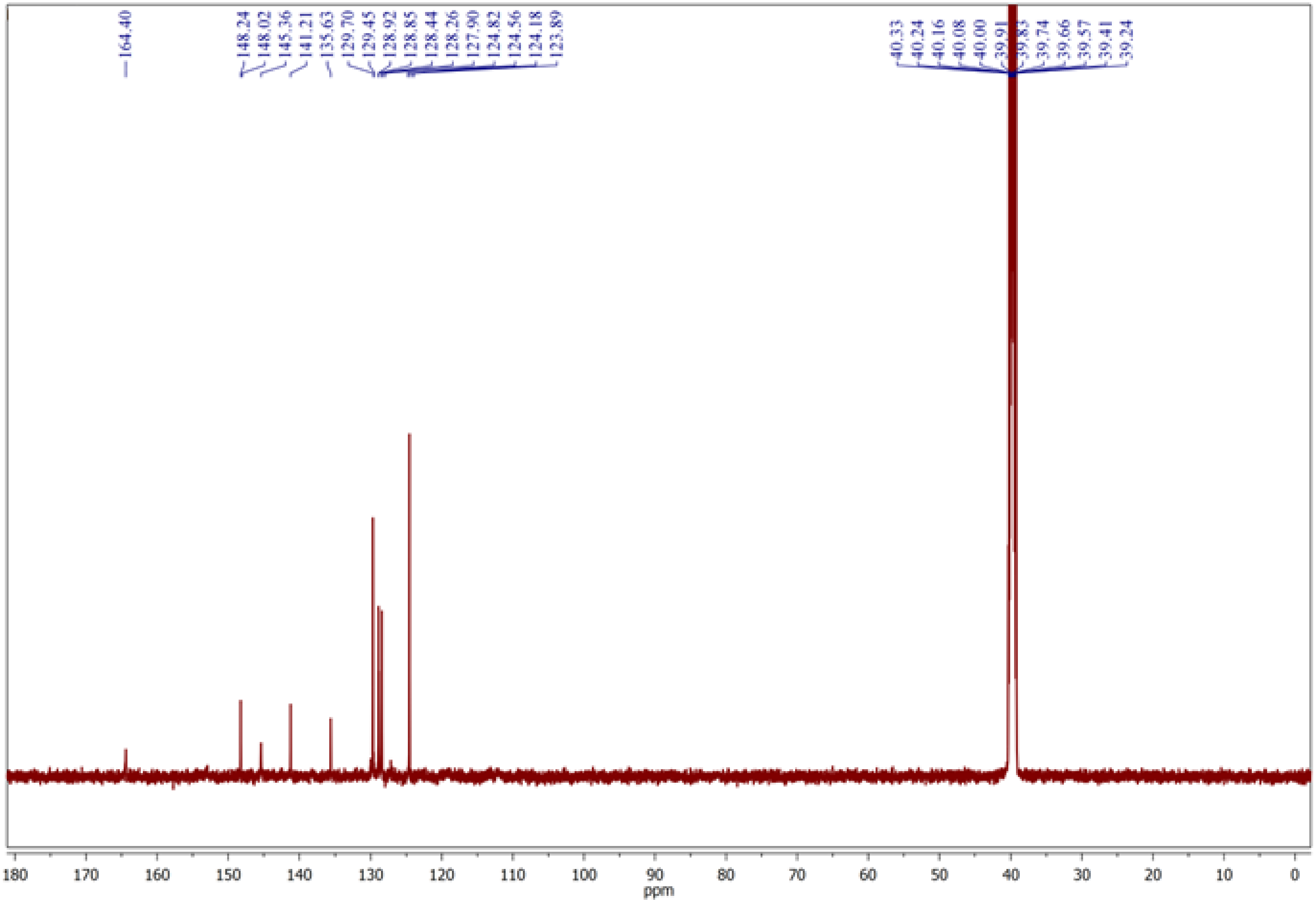

**Figure S18: IR spectrum of compound <u>4c</u> (KBr, υ_{max} cm^{-1})**
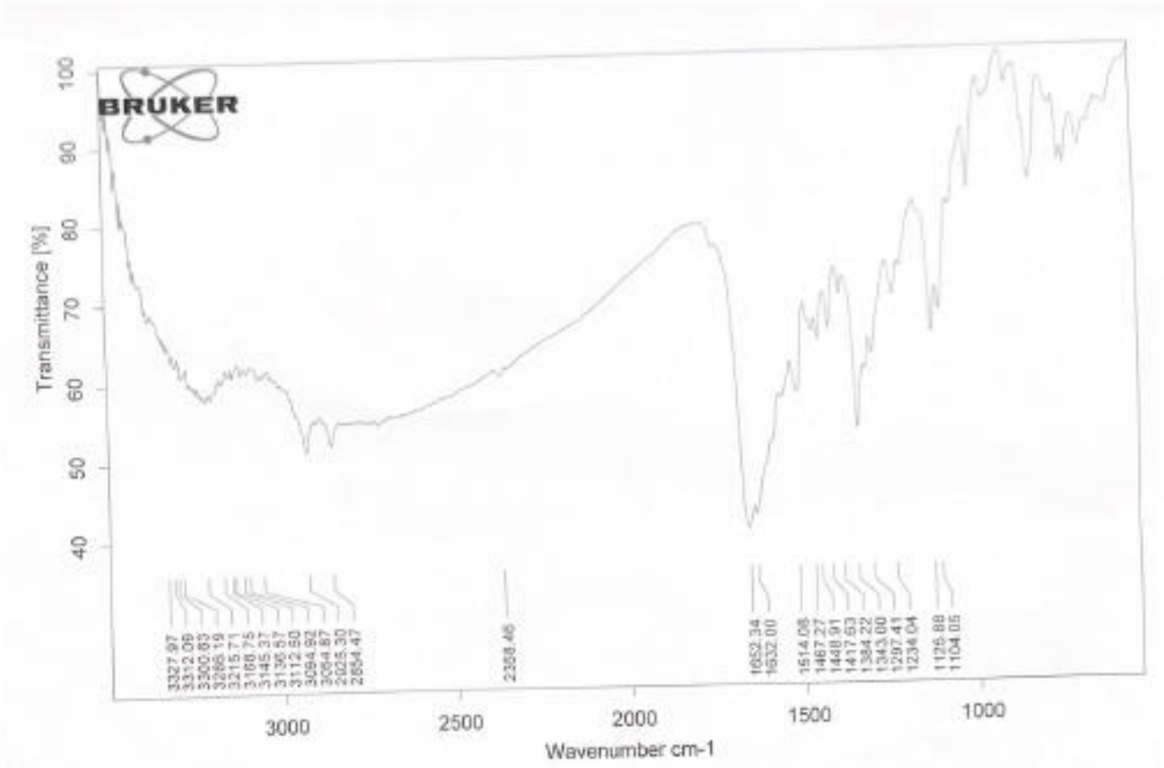

Figure S19: ^{1}H- NMR spectrum of compound 4d (400 MHz, DMSO-*d6*)

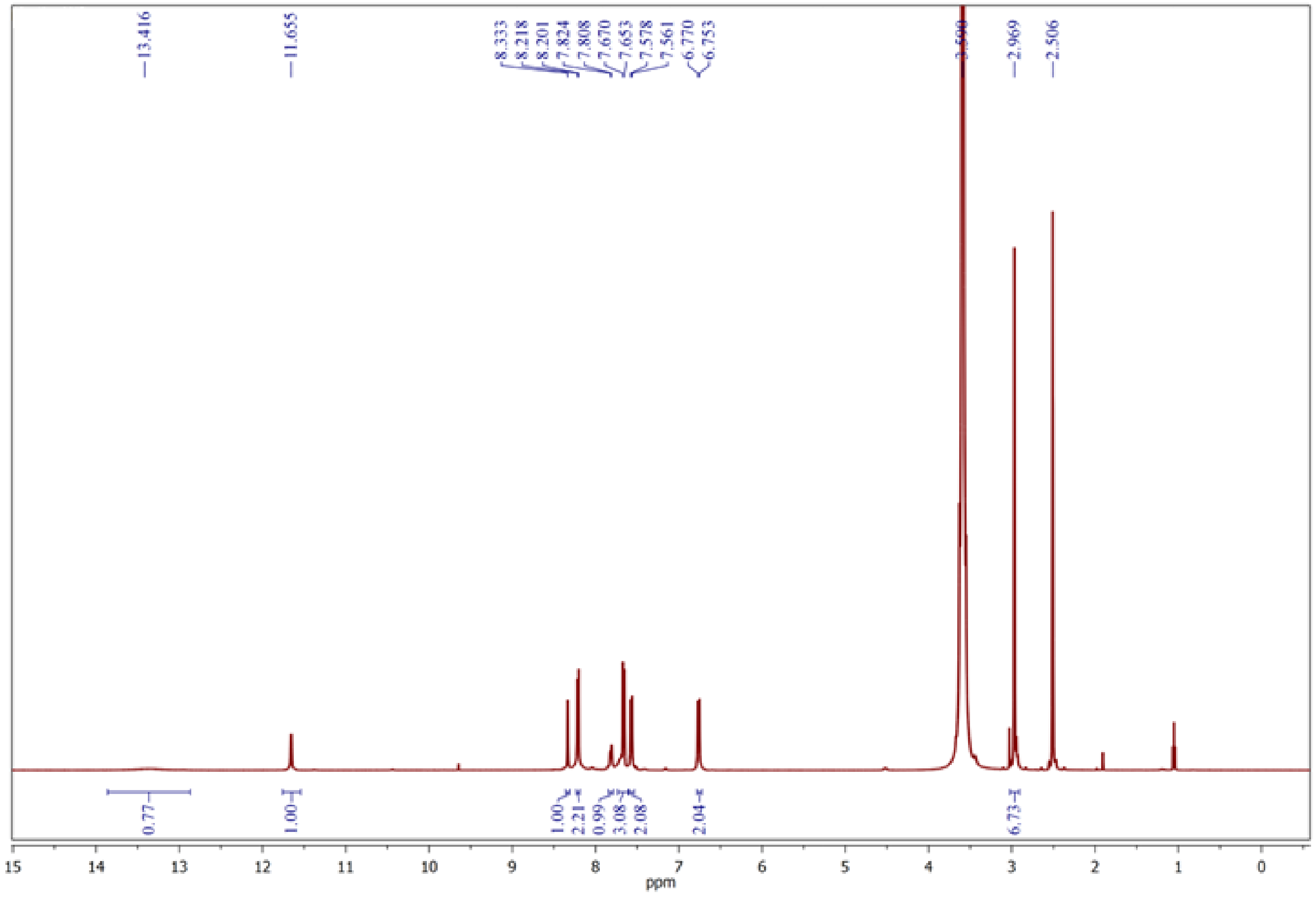

Figure S20: ^{13}C- NMR spectrum of compound 4d (100 MHz, DMSO-*d6*)

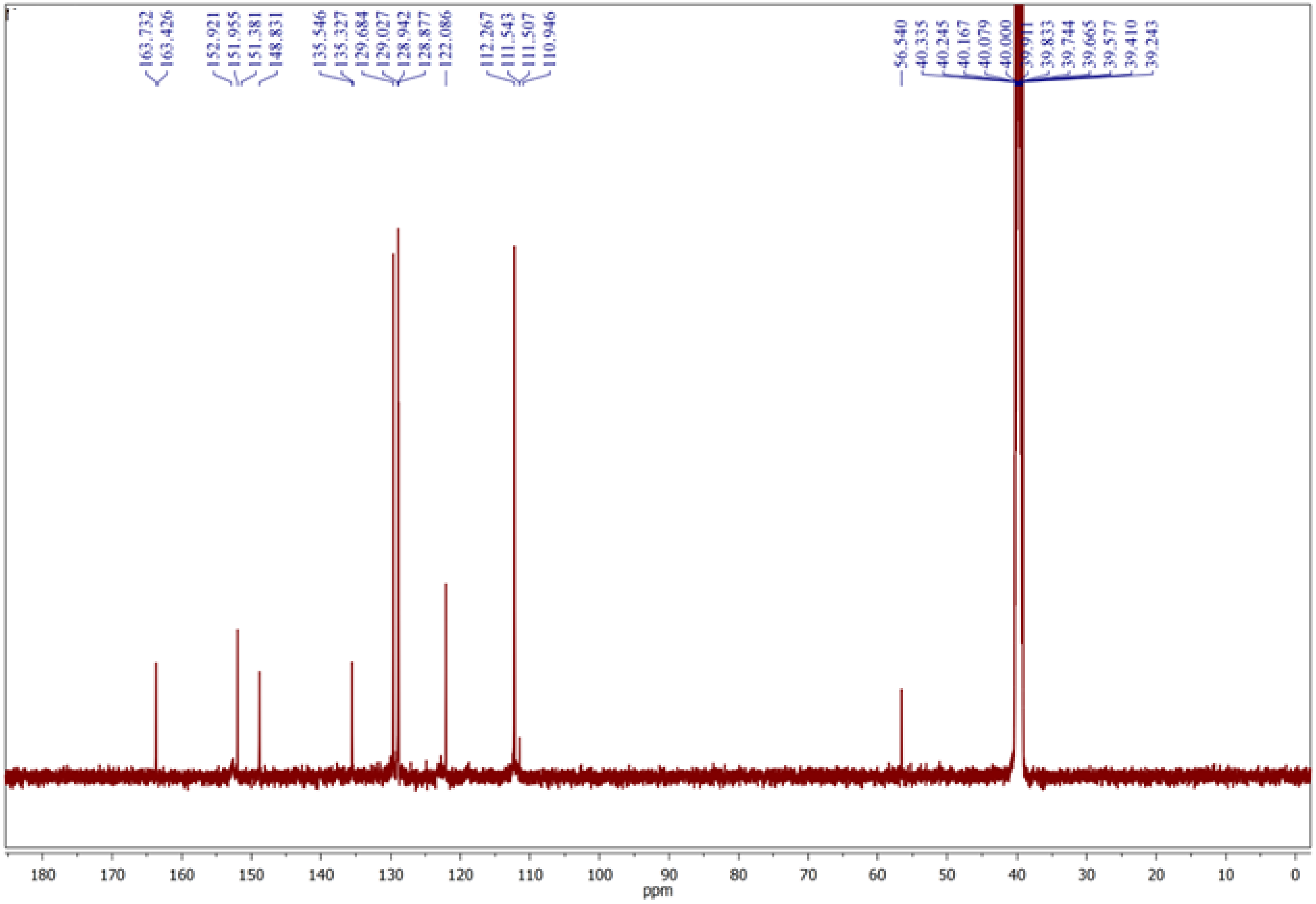

Figure S21: IR spectrum of compound <u>4d</u> (KBr, υ_{max} cm^{-1})

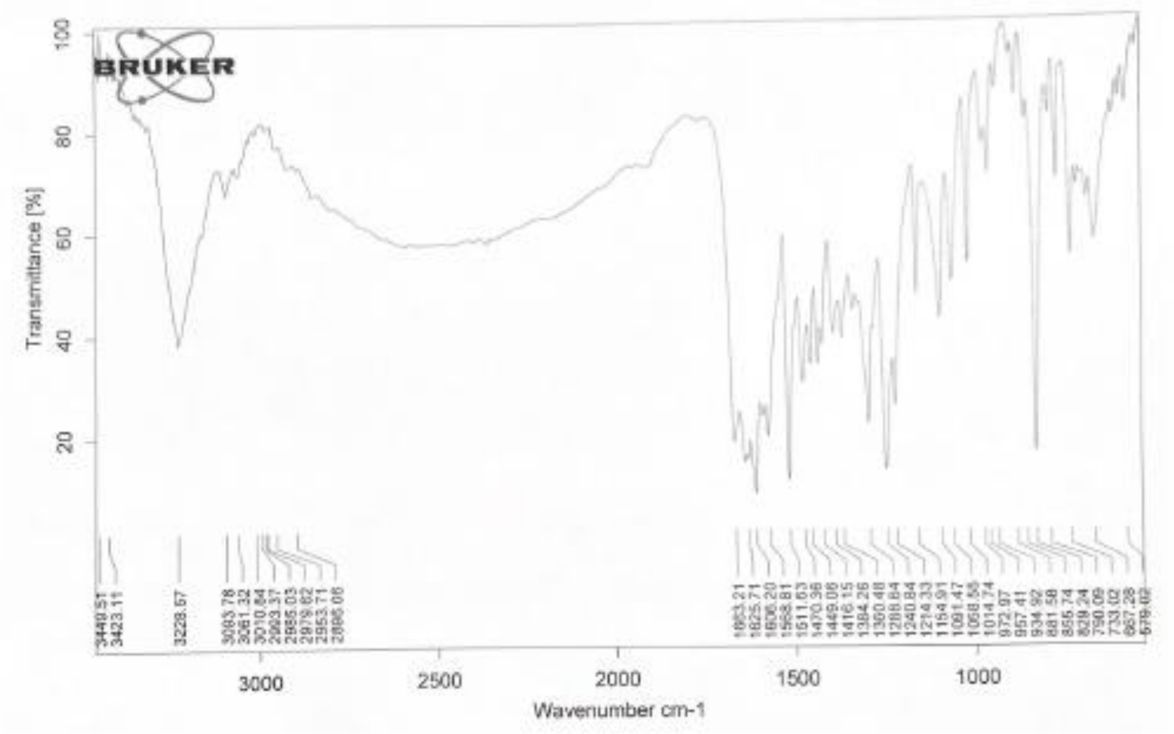

Figure S22: ^{1}H- NMR spectrum of compound <u>4e</u> (500 MHz, DMSO-*d6*)

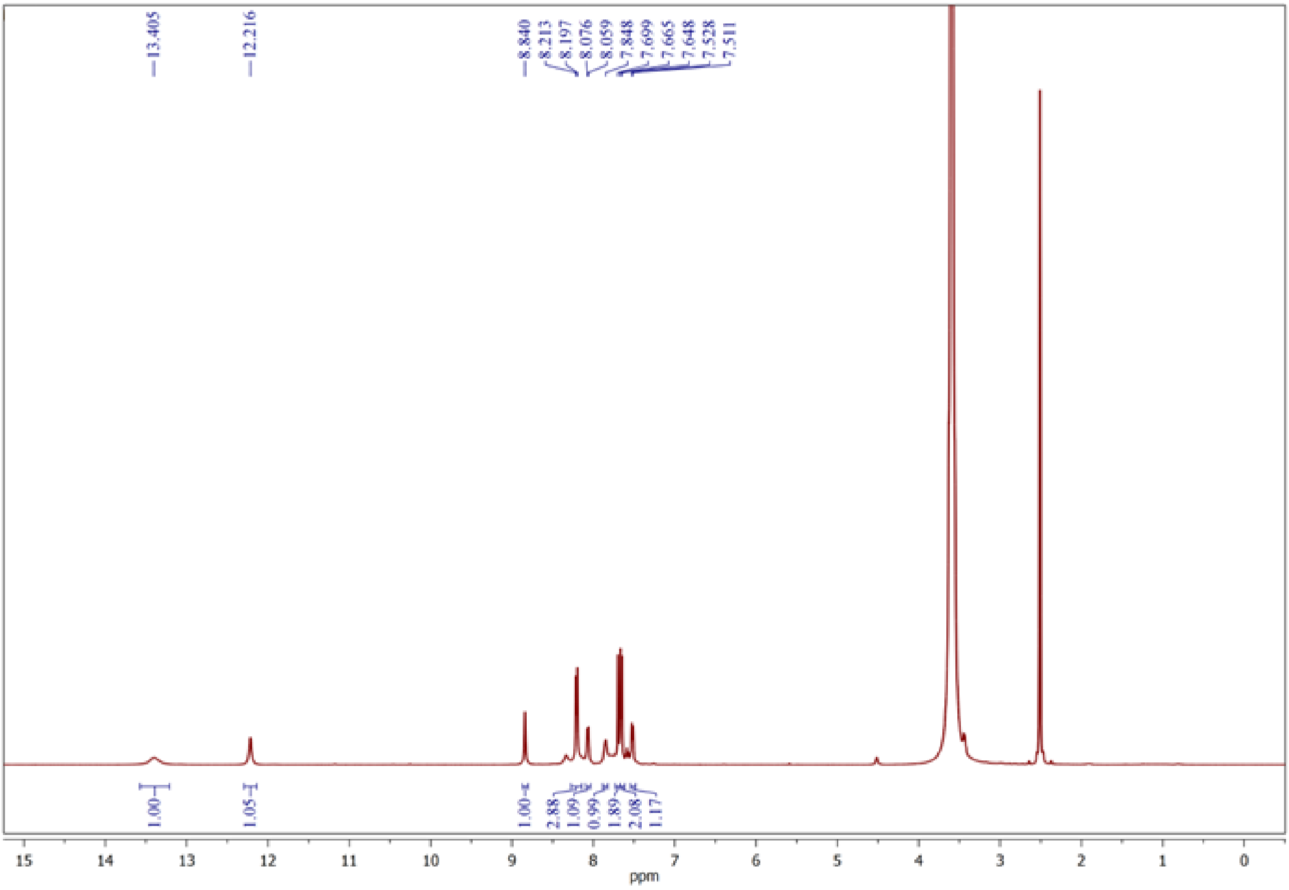

Figure S23: ^{13}C- NMR spectrum of compound <u>4e</u> (125 MHz, DMSO-*d6*)

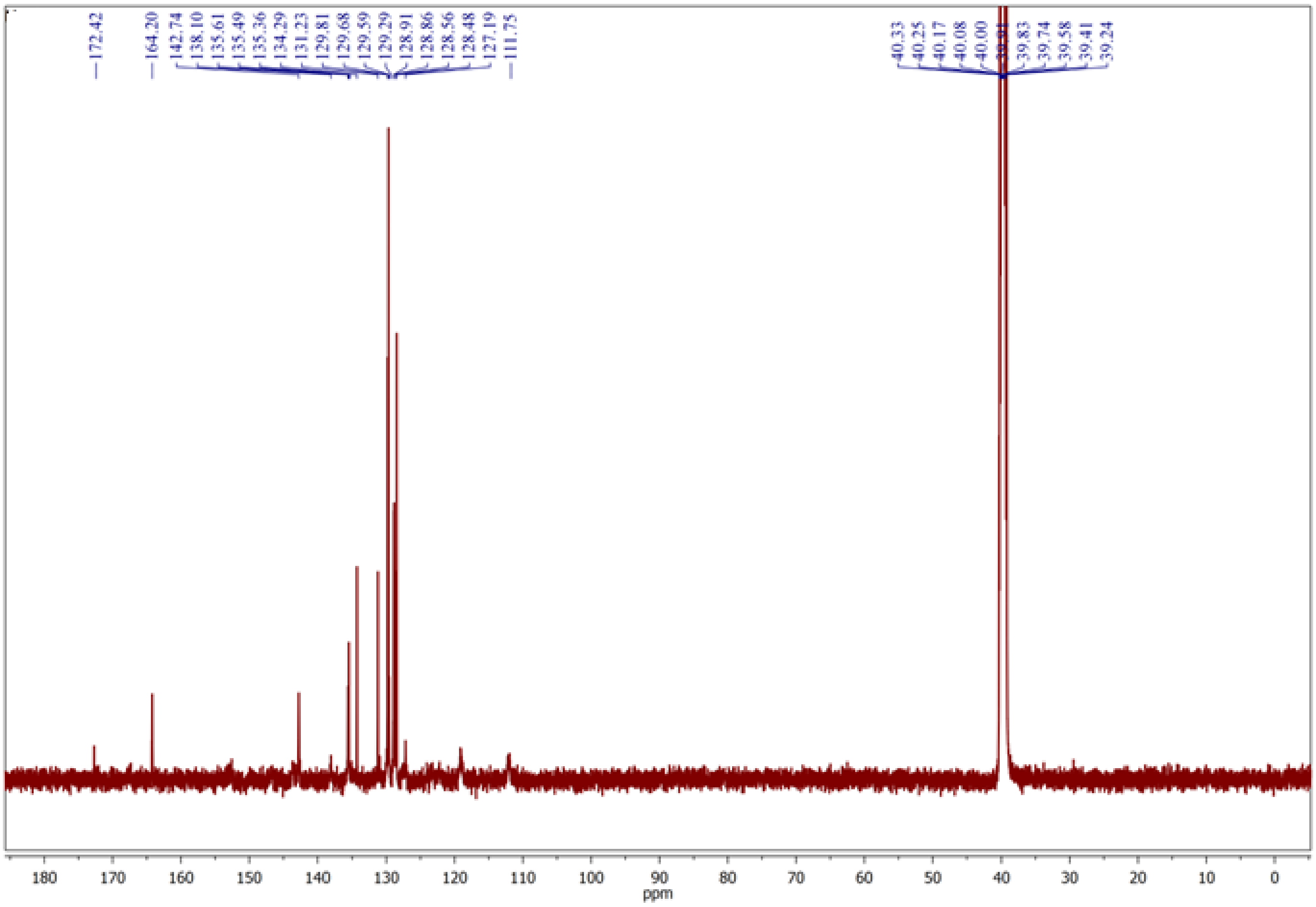

Figure S24: IR spectrum of compound <u>4e</u> (KBr, υ_{max} cm^{-1})

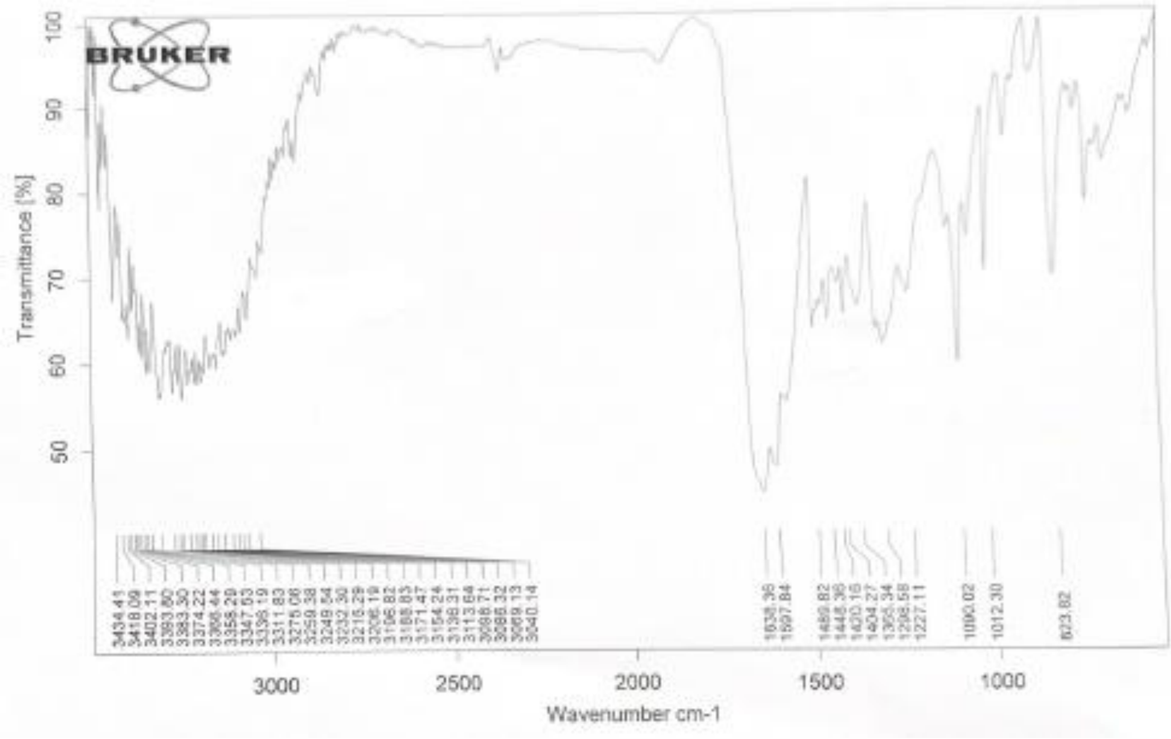

Figure S25: ^{1}H- NMR spectrum of compound <u>4f</u> (500 MHz, DMSO-*d6*)

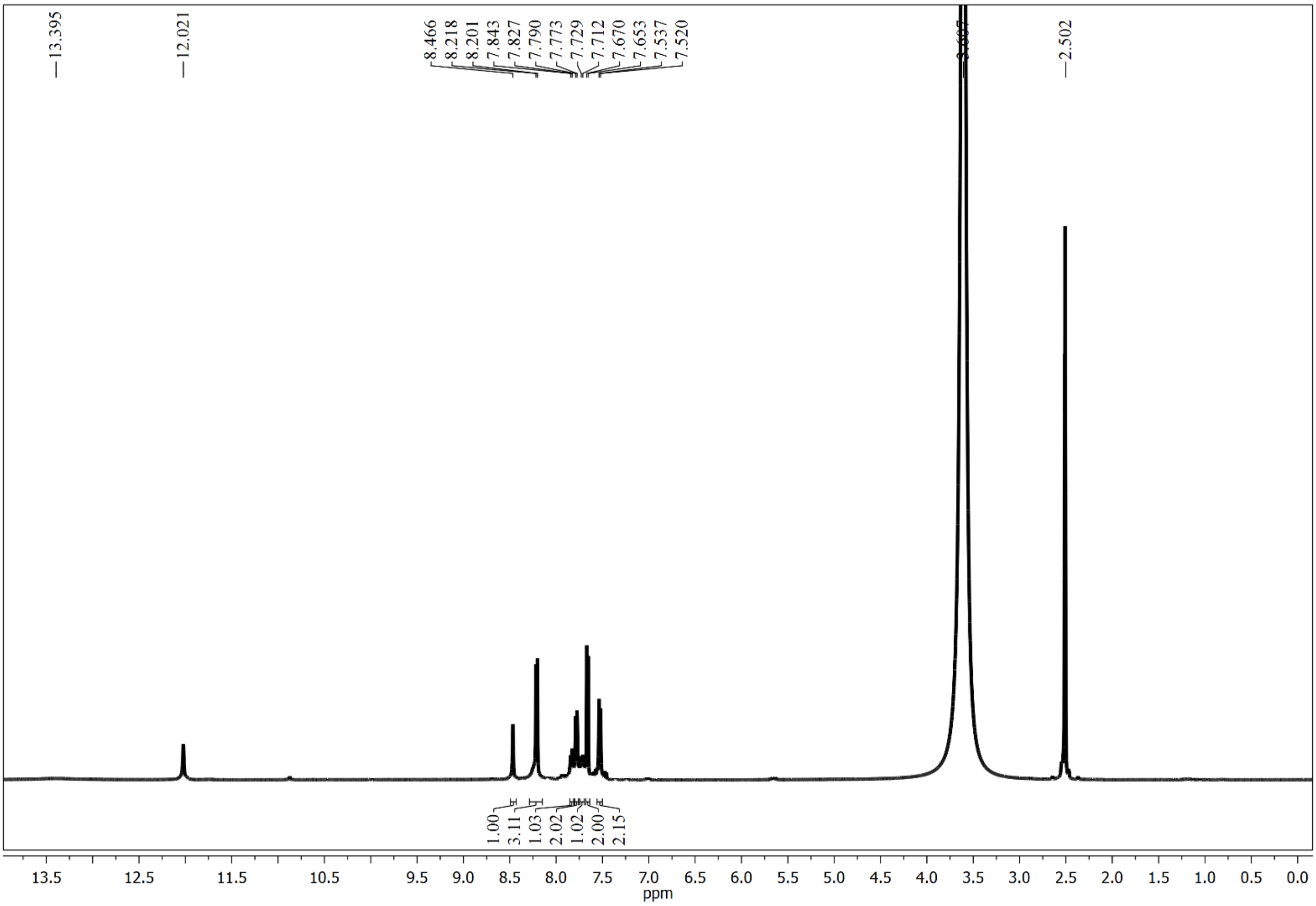

Figure S26: ^{13}C- NMR spectrum of compound 4f (125 MHz, DMSO-*d6*)

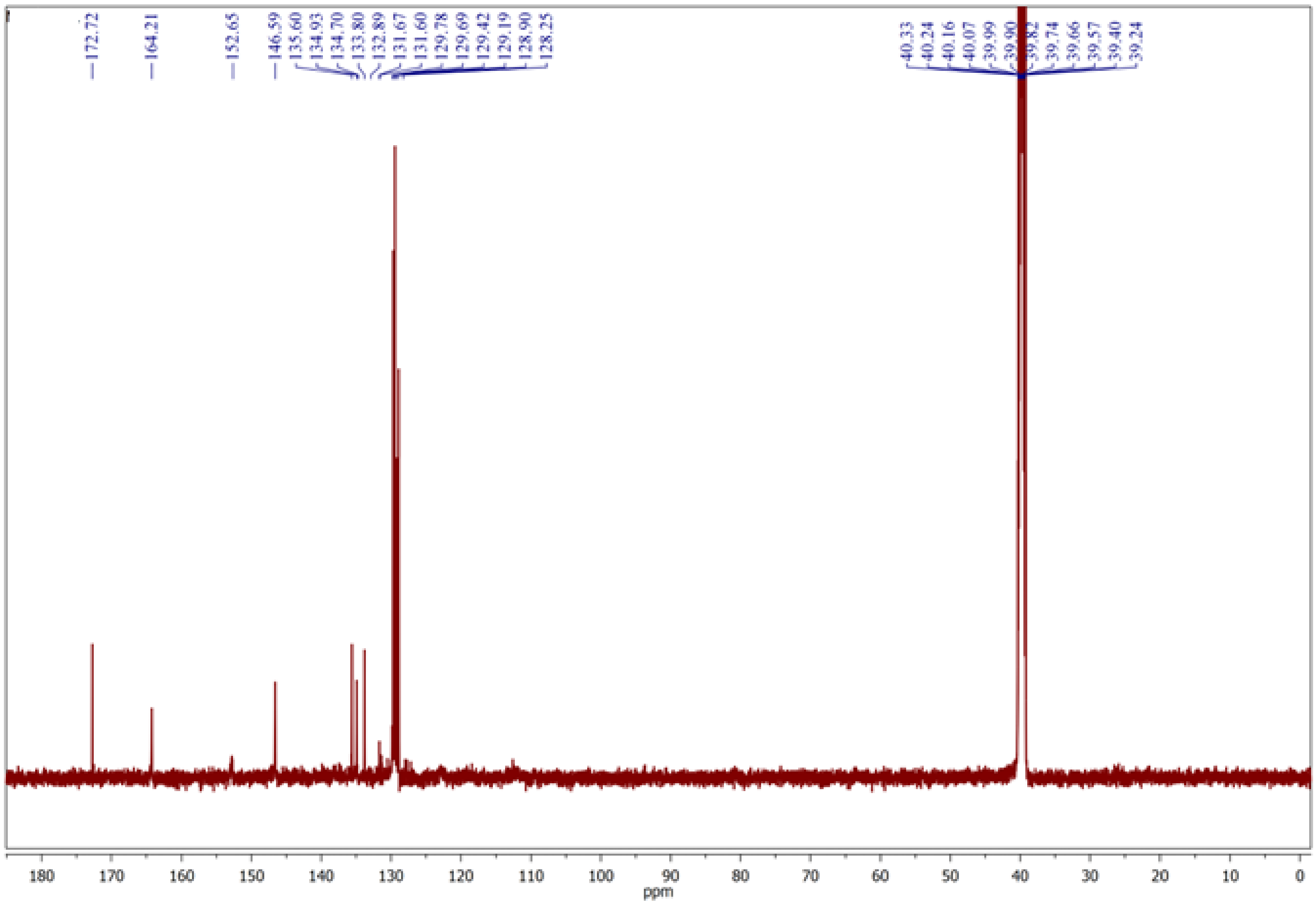

**Figure S27: IR spectrum of compound <u>4f</u> (KBr, υ_{max} cm^{-1})**
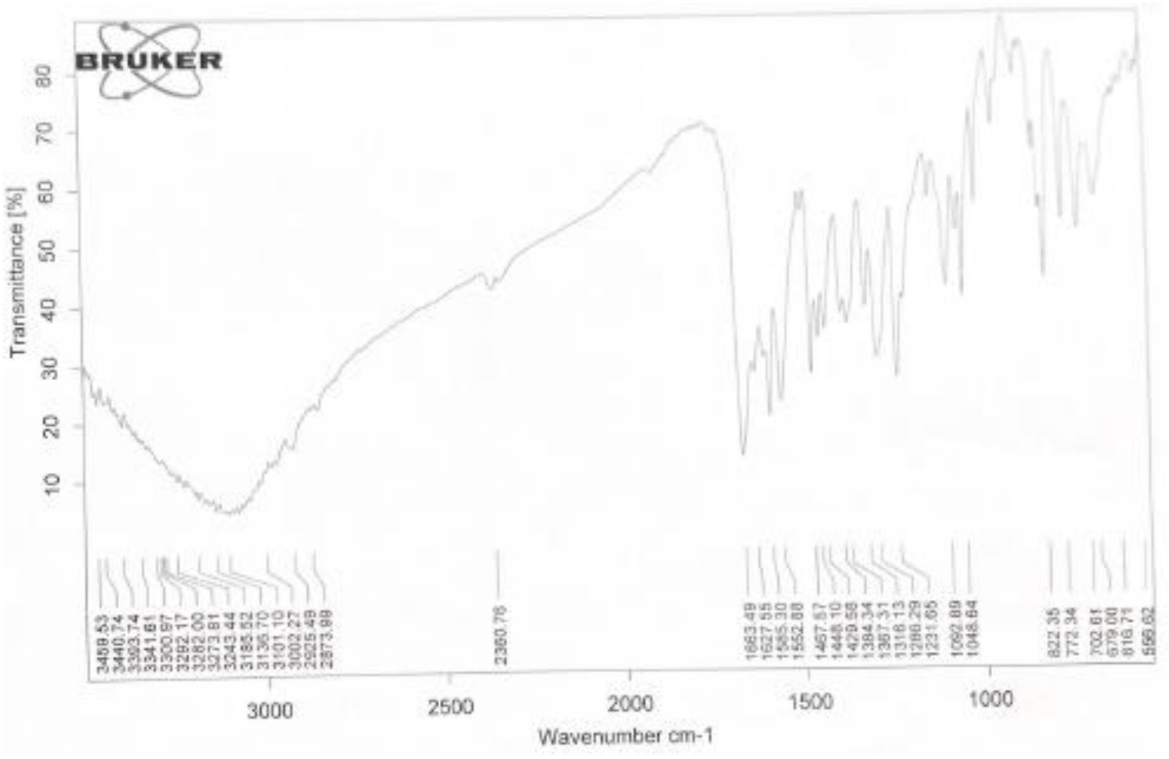

Figure S28: ^{1}H- NMR spectrum of compound 4g (500 MHz, DMSO-*d6*)

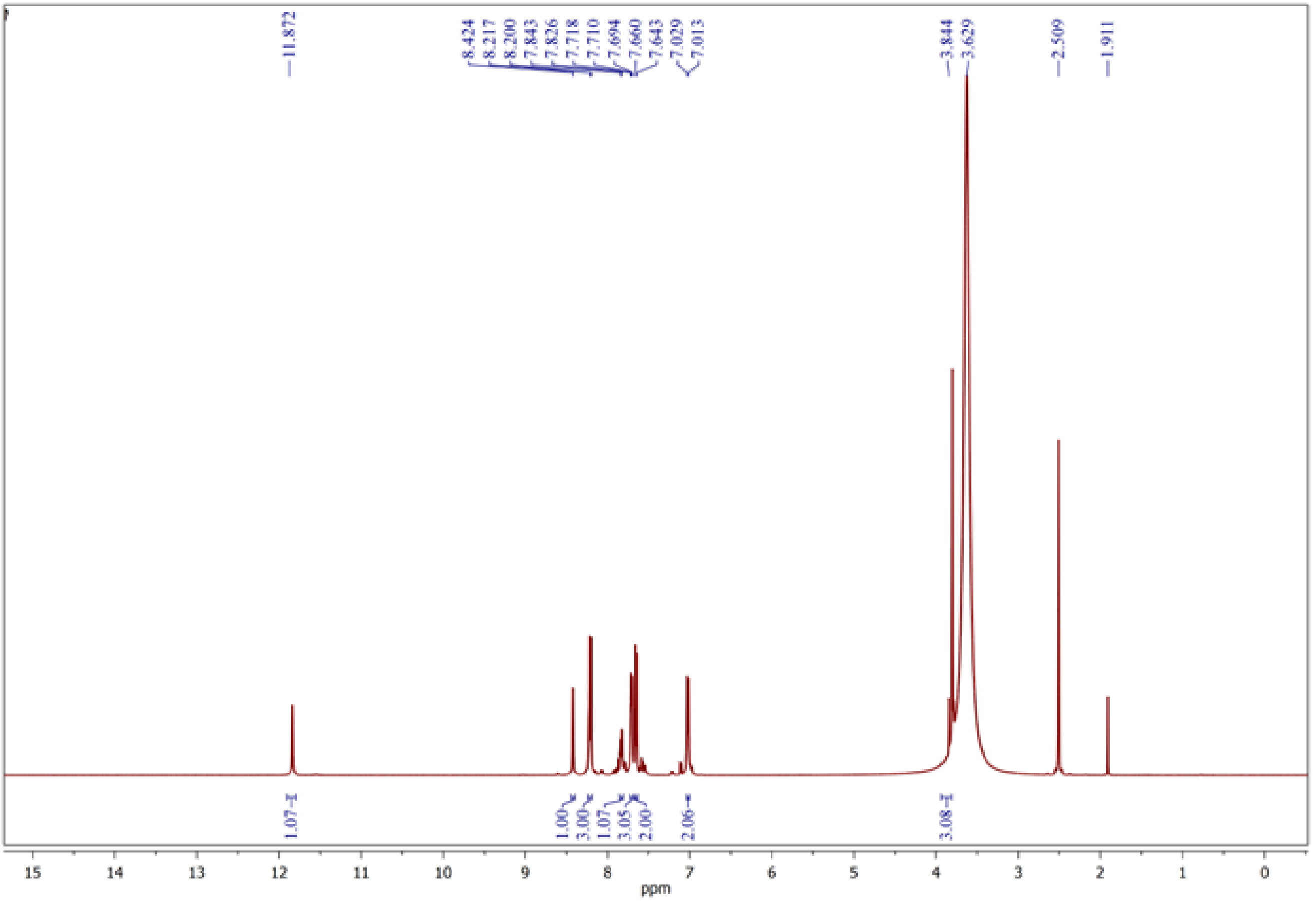

Figure S29: ^{13}**C- NMR spectrum of compound** **4g** **(125 MHz, DMSO-*d6*)**

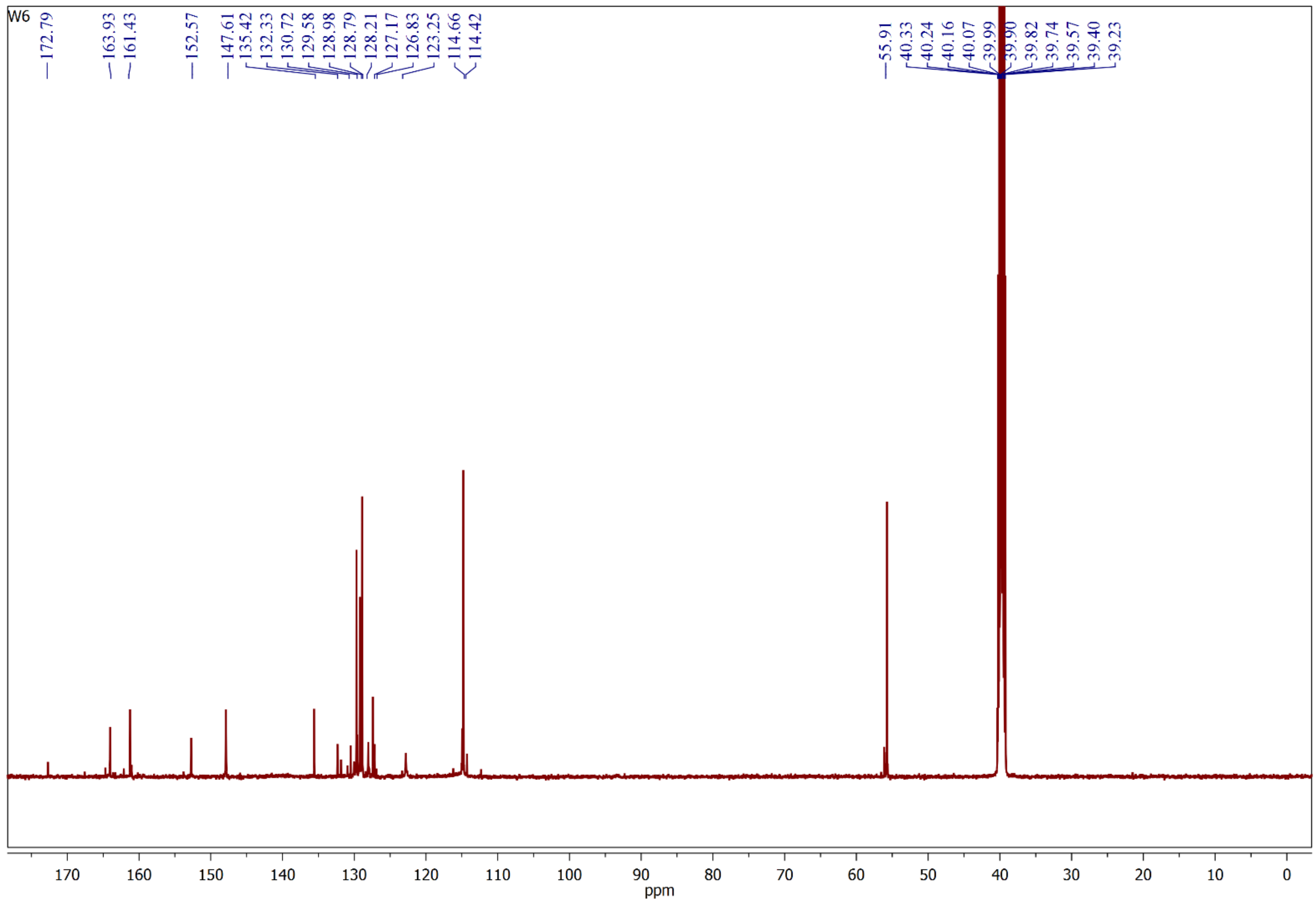

Figure S30: IR spectrum of compound 4g (KBr, υ_{max} cm^{-1})

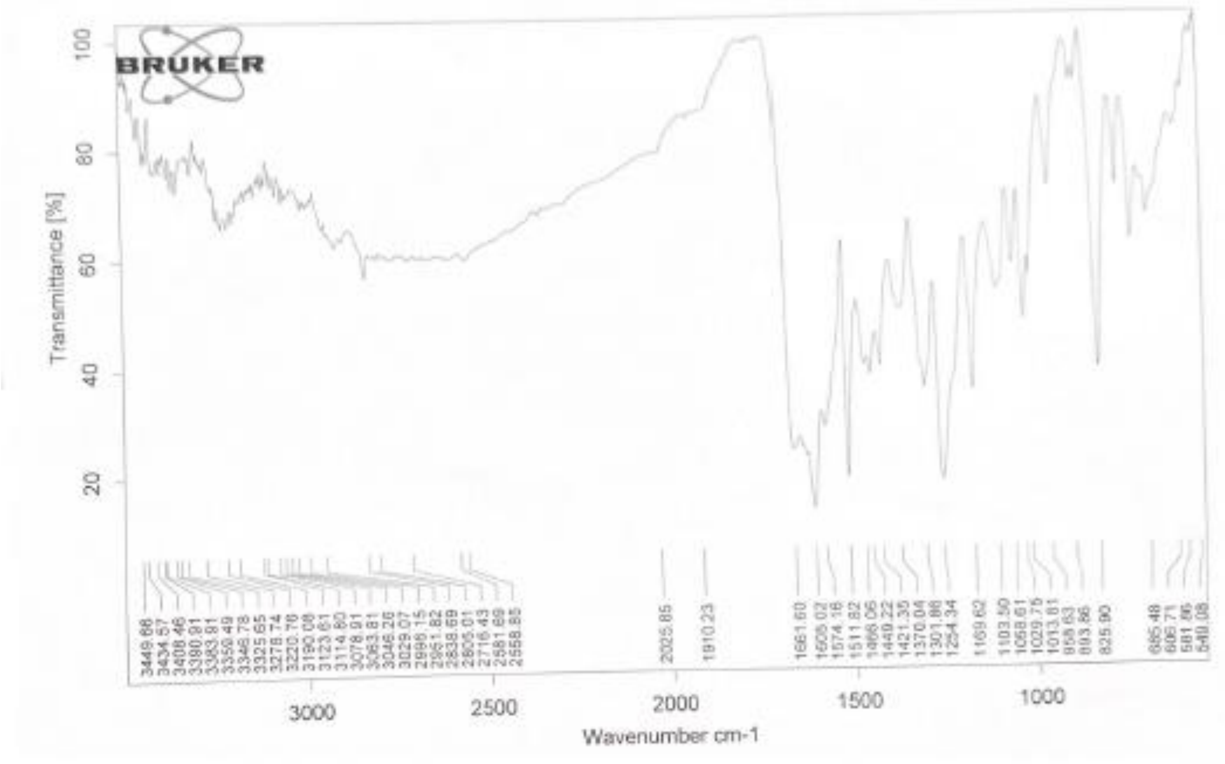

Figure S31: ^{1}H- NMR spectrum of compound 4h (500 MHz, DMSO-*d6*)

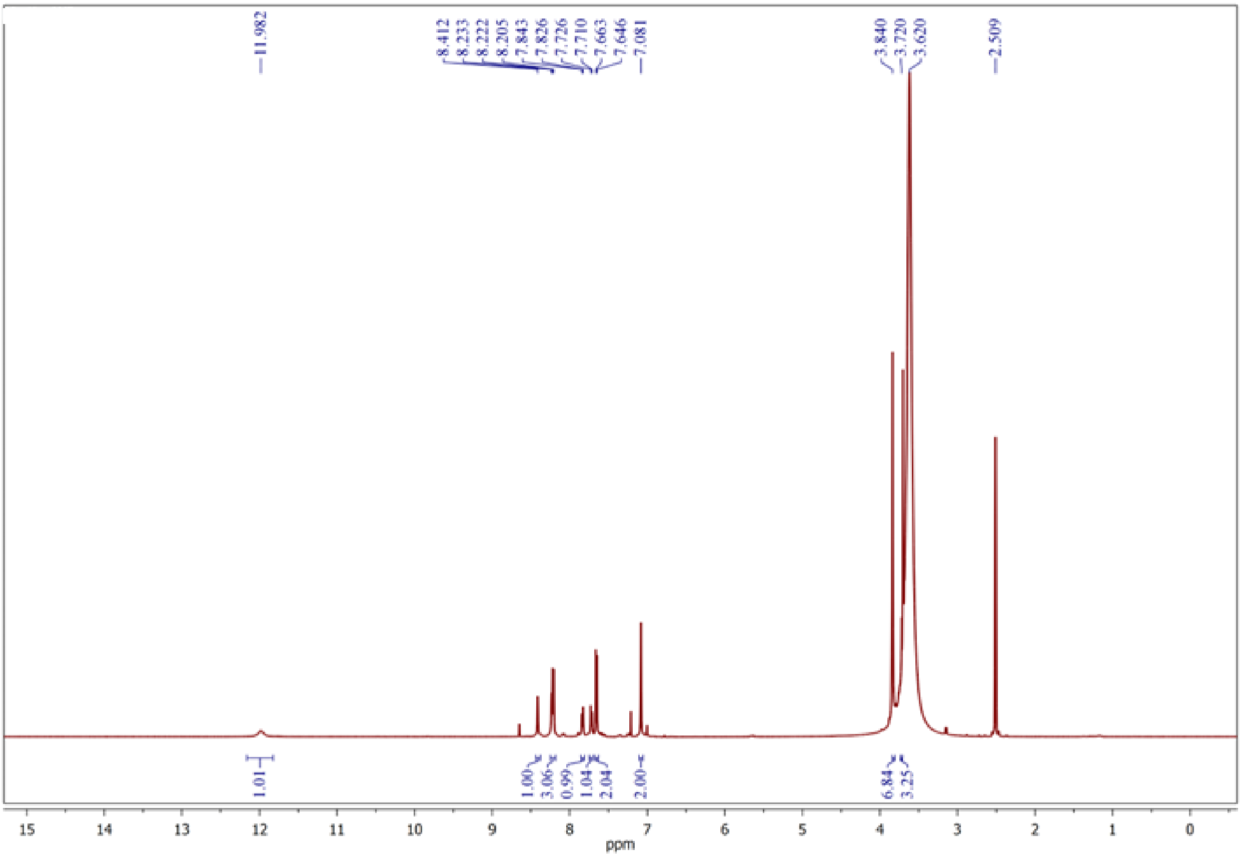

Figure S32: ^{13}C- NMR spectrum of compound 4h (125 MHz, DMSO-*d6*)

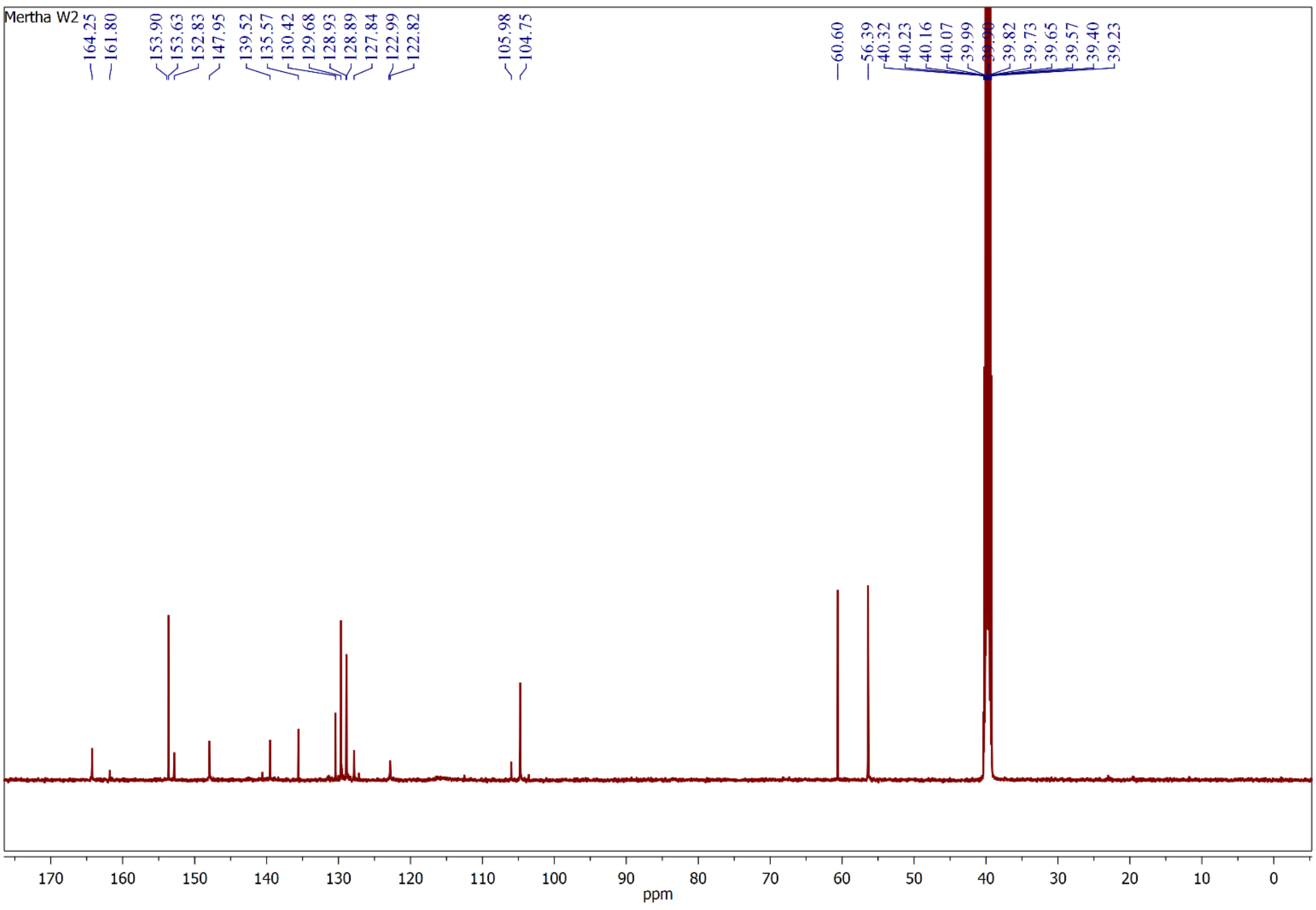

Figure S33: IR spectrum of compound 4h (KBr, υ_{max} cm^{-1})

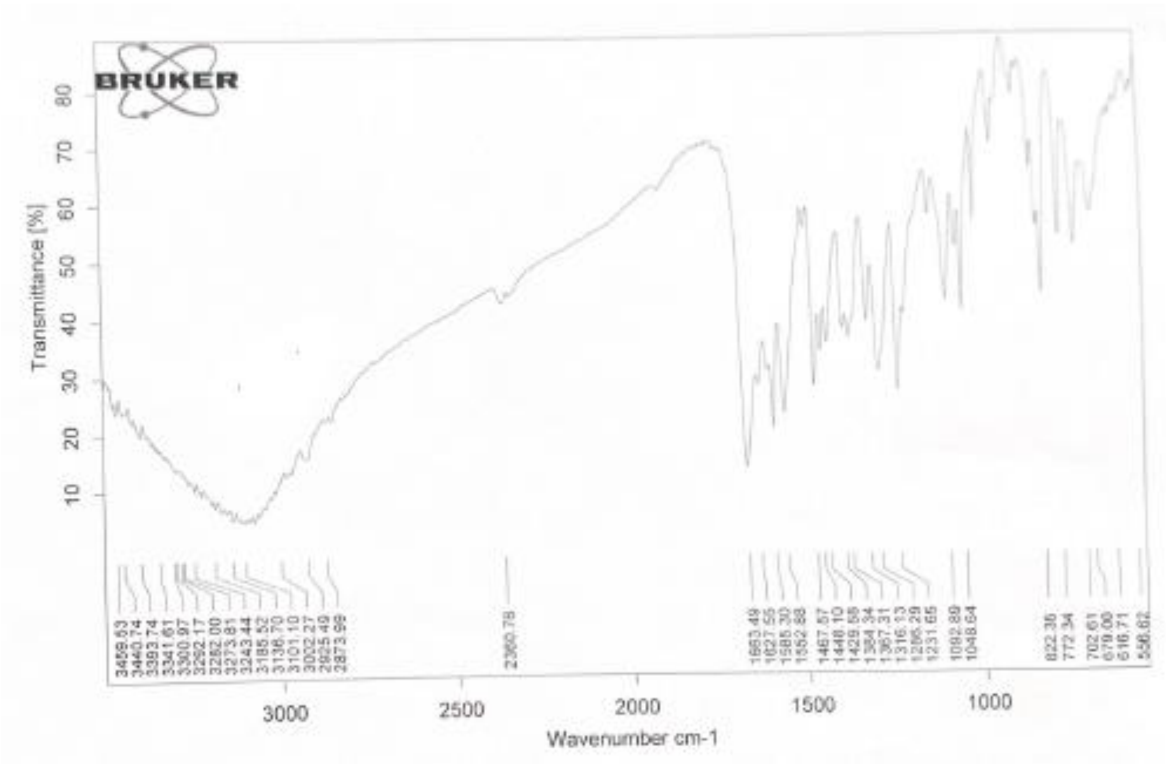

Figure S34: ^{1}H- NMR spectrum of compound 4i (500 MHz, DMSO-*d6*)

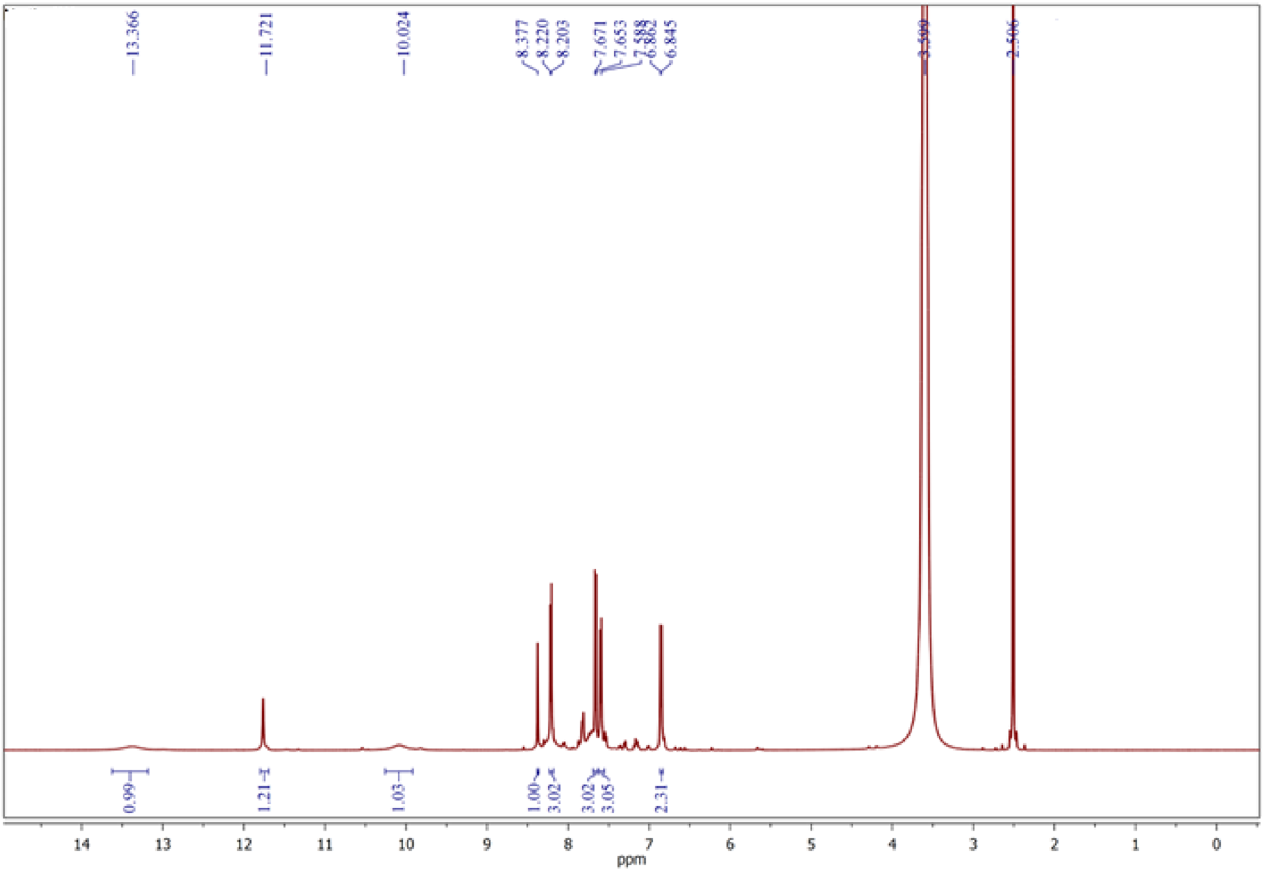

Figure S35: ^{13}C- NMR spectrum of compound 4i (125 MHz, DMSO-*d6*)

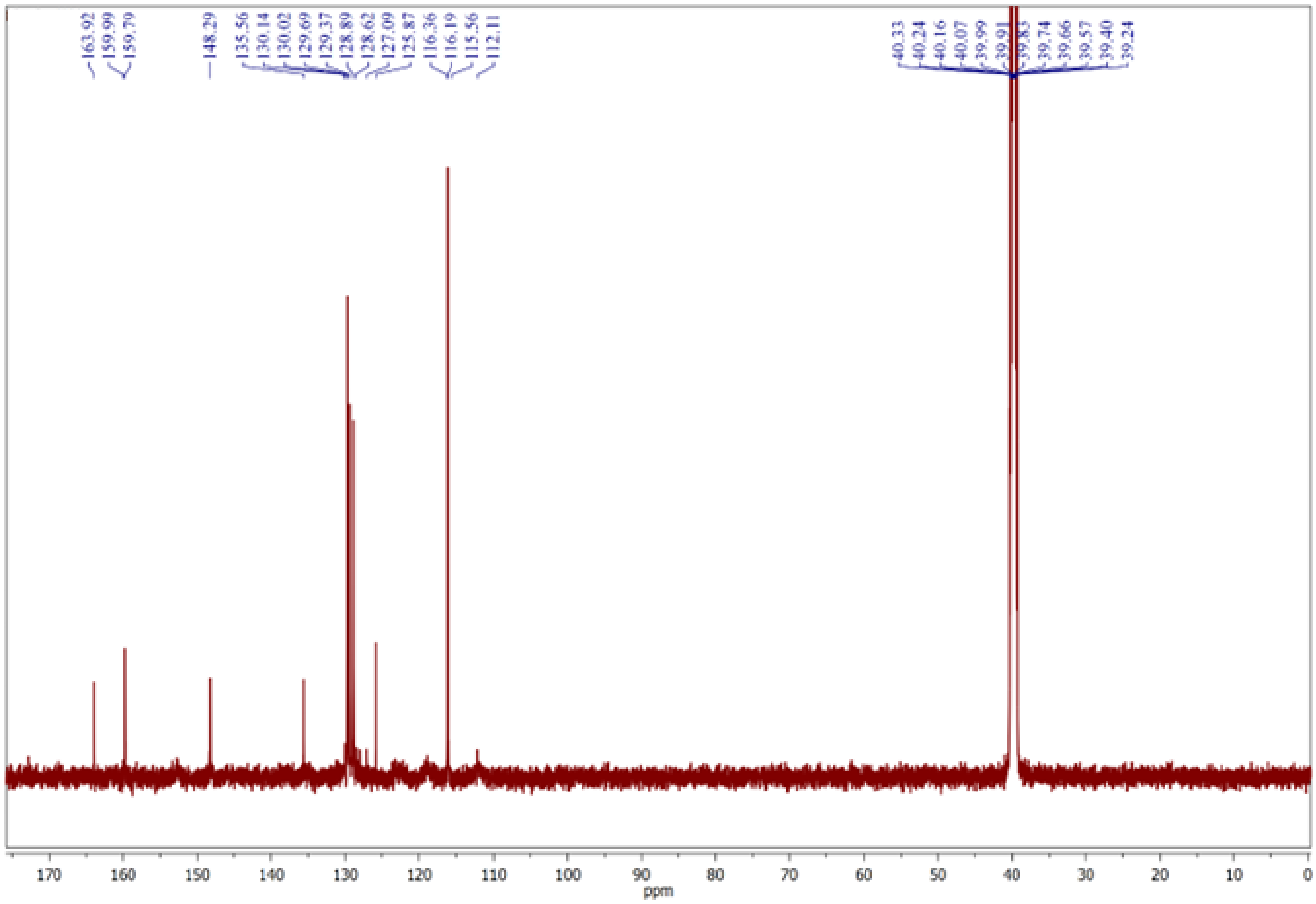

Figure S36: IR spectrum of compound 4i (KBr, υ_{max} cm^{-1})

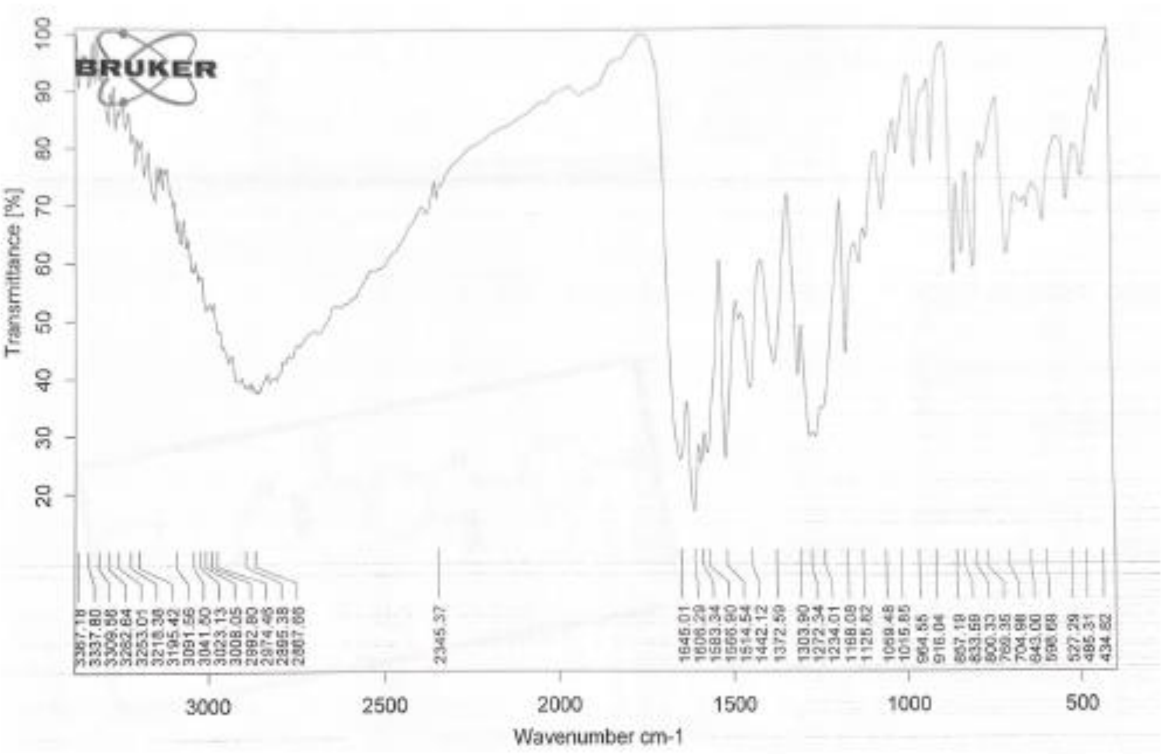

Figure S37: ^{1}H- NMR spectrum of compound <u>4j</u> (500 MHz, DMSO-*d6*)

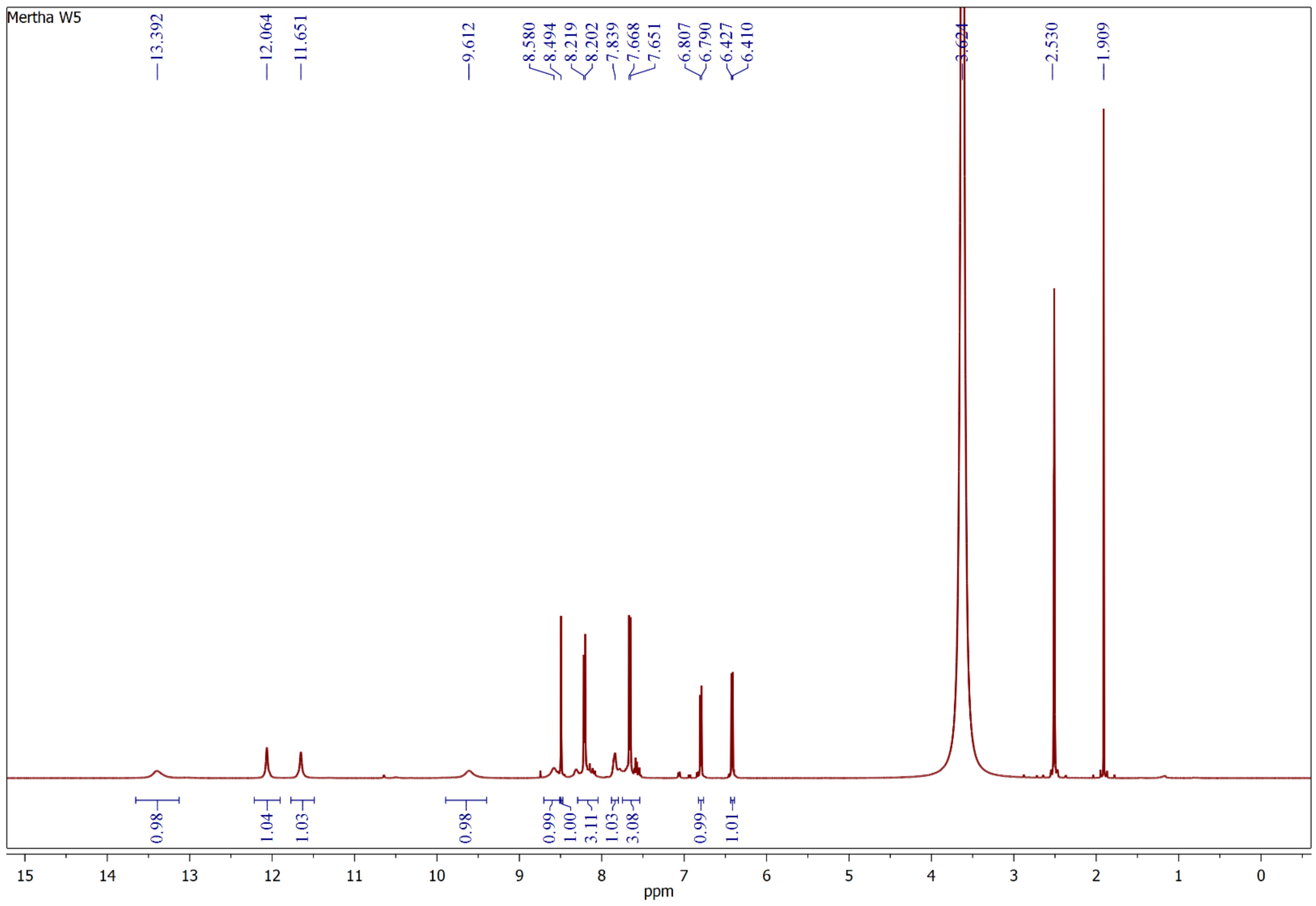

Figure S38: ^{13}C- NMR spectrum of compound 4j (125 MHz, DMSO-*d6*)

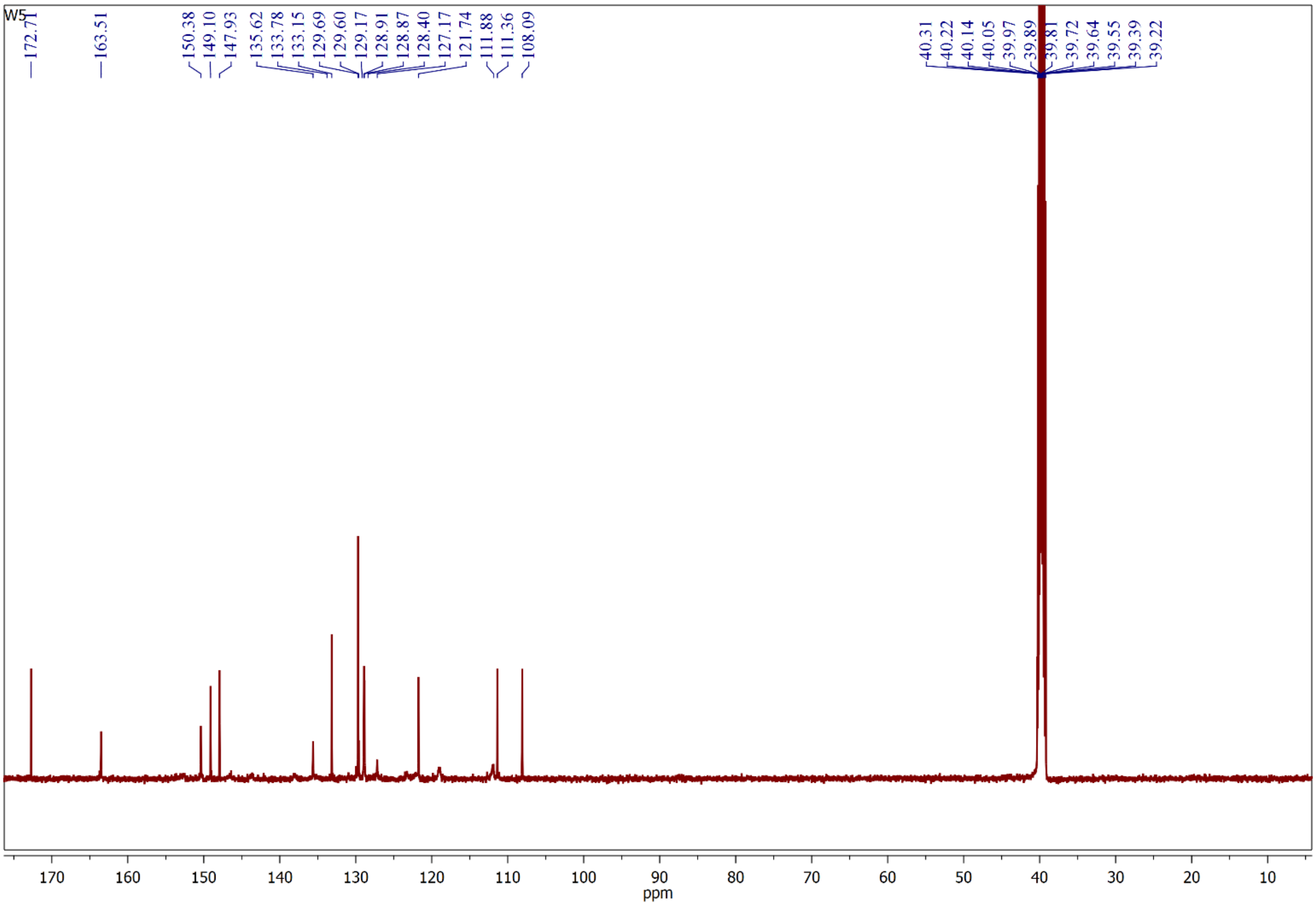

Figure S39: IR spectrum of compound 4j (KBr, υ_{max} cm^{-1})

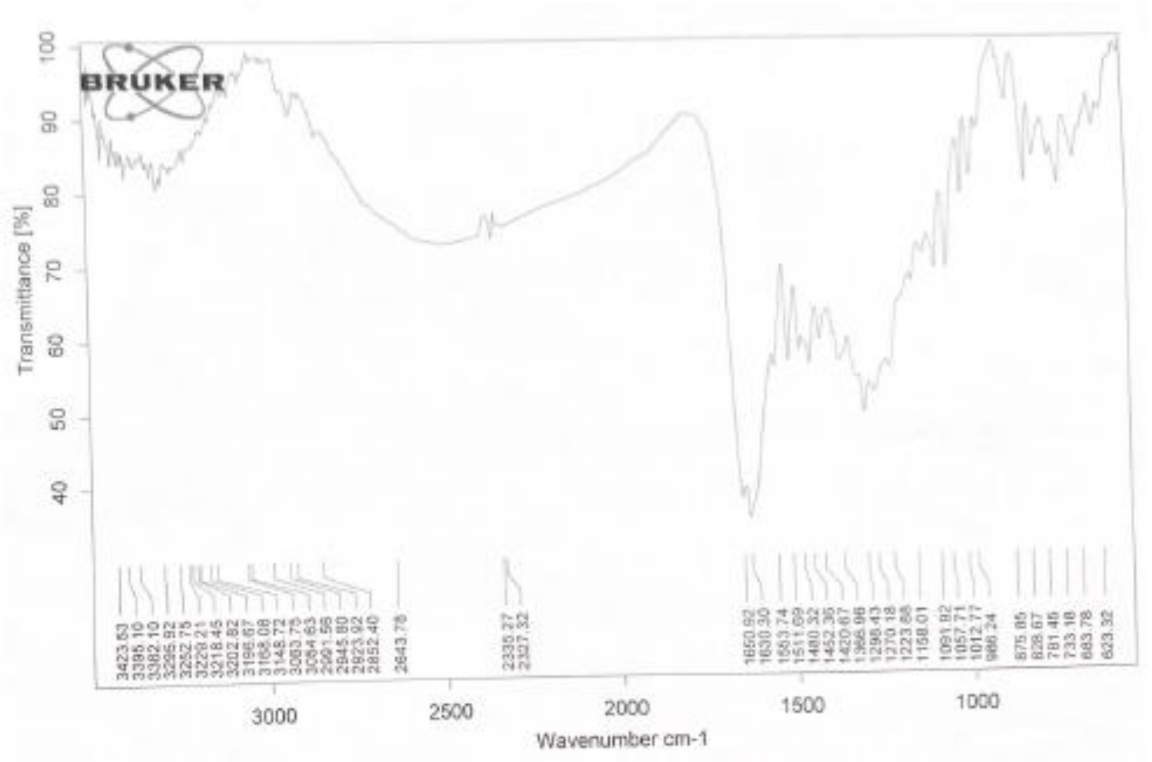

Figure S40: ^{1}H- NMR spectrum of compound 6 (500 MHz, DMSO-*d6*)

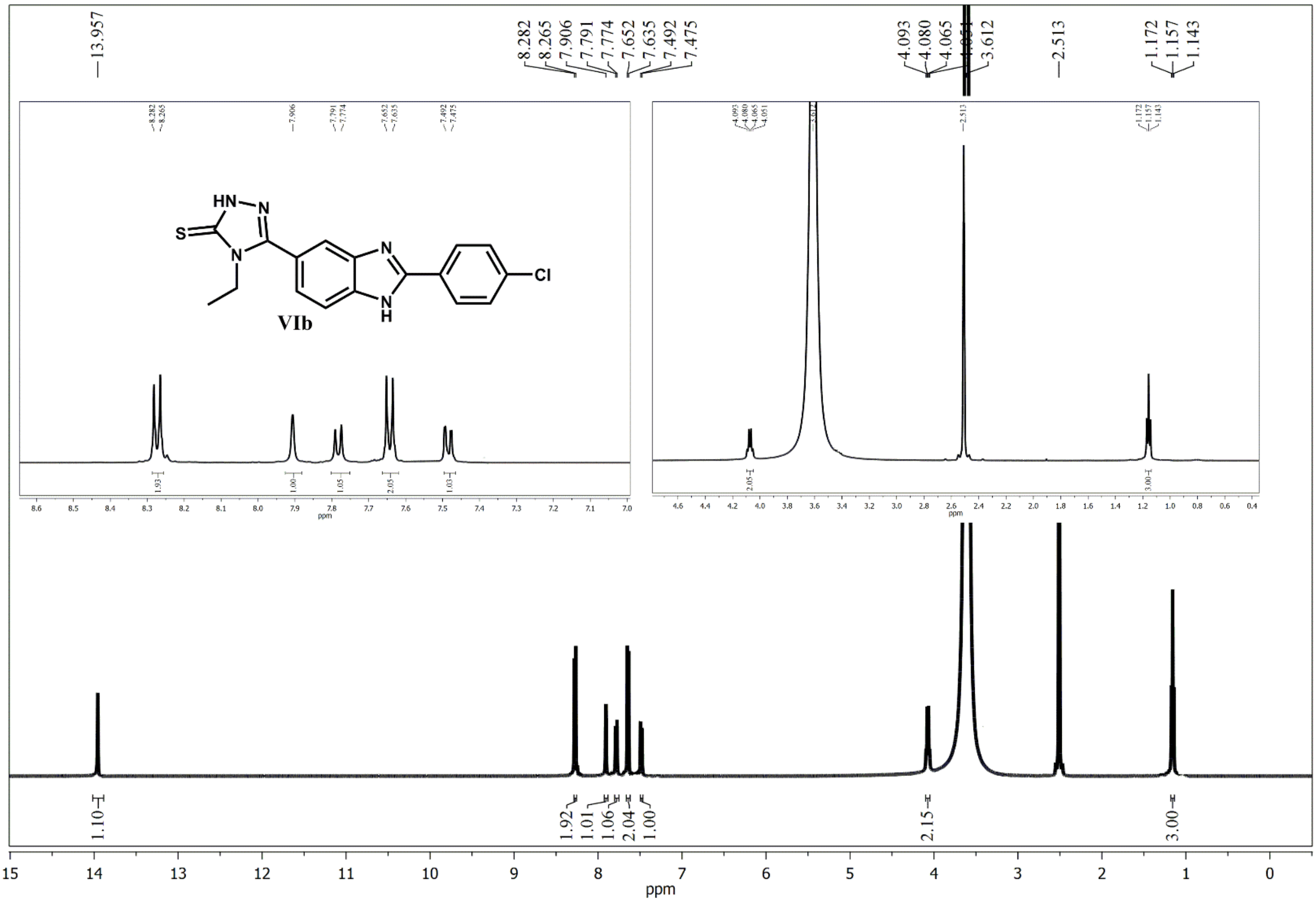

Figure S41: ^{13}C- NMR spectrum of compound 6 (125 MHz, DMSO-*d6*)

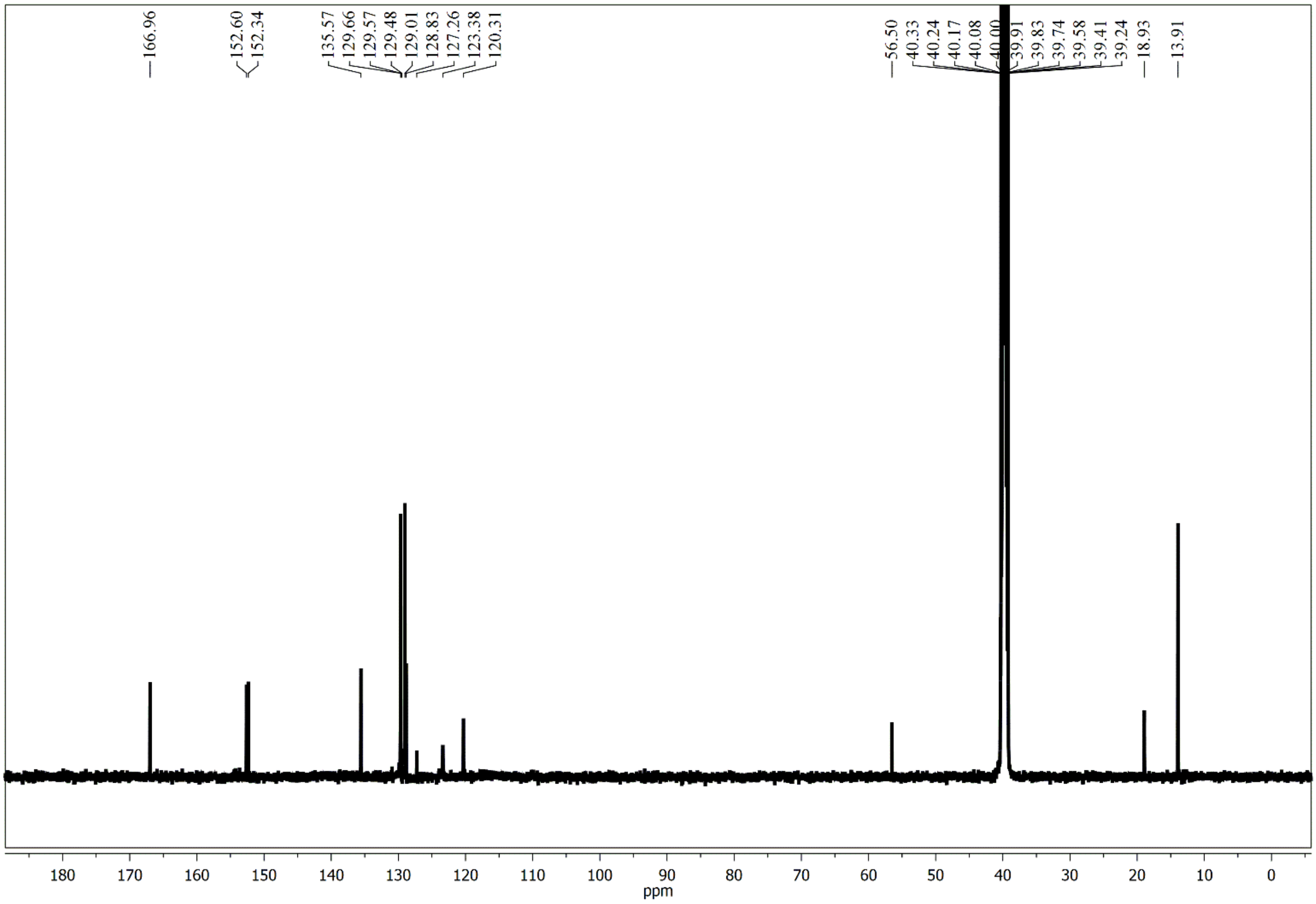

Figure S42: IR spectrum of compound 6 (KBr, υ_{max} cm^{-1})

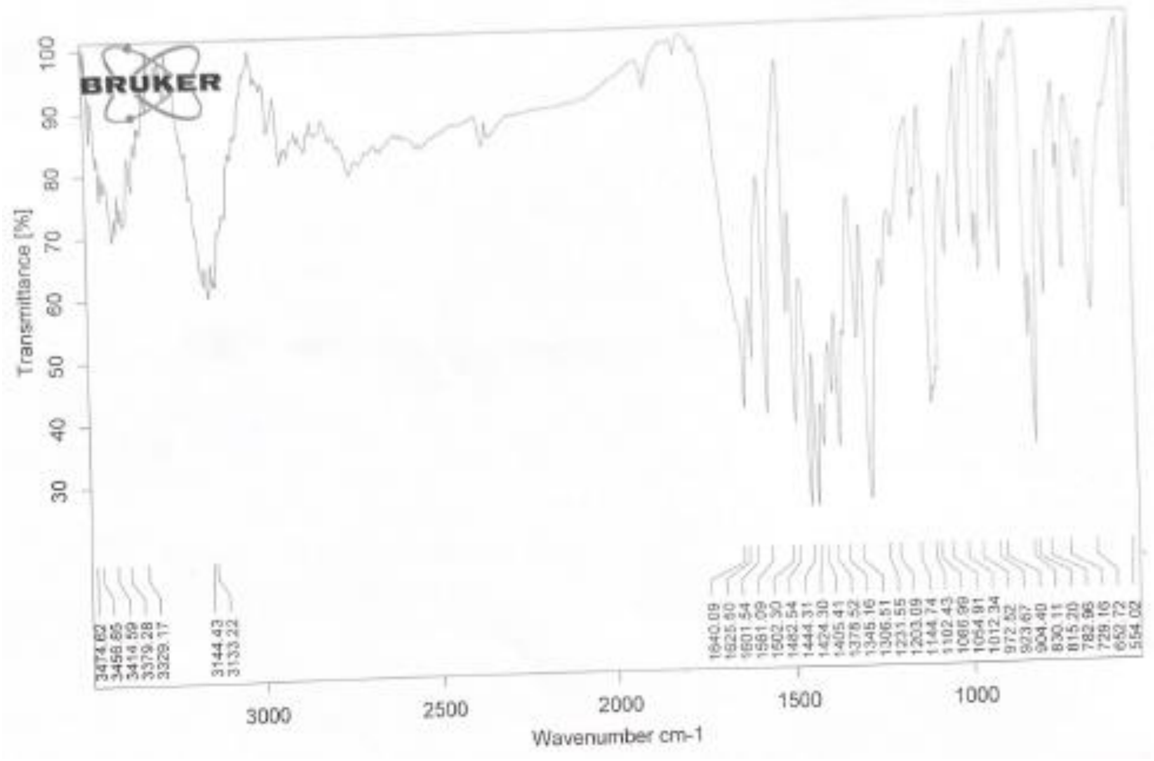

Panel/Cell Line	Growth Percent
Leukemia	
CCRF-CEM	9.87
HL-60(TB)	6.58
K-562	9.68
MOLT-4	4.24
RPMI-8226	10.78
SR	-8.49
Non-Small Cell Lung Cancer	
A549/ATCC	34.54
EKVX	49.03
HOP-62	44.78
HOP-92	59.68
NCI-H226	54.78
NCI-H23	16.25
NCI-H322M	70.07
NCI-H460	5.44
NCI-H522	33.03
Colon Cancer	
COLO 205	2.28
HCC-2998	14.91
HCT-116	14.60
HCT-15	29.88
HT29	8.25
KM12	13.03
SW-620	12.66
CNS Cancer	
SF-268	48.50
SF-295	21.68
SF-539	41.66
SNB-19	52.05
SNB-75	69.29
U251	22.33
Melanoma	
LOX IMVI	-0.91
MALME-3M	7.79
M14	25.91
MDA-MB-435	47.58
SK-MEL-2	66.72
SK-MEL-28	45.35
SK-MEL-5	2.58
UACC-257	25.08
UACC-62	43.65
Ovarian Cancer	
IGROV1	19.12
OVCAR-3	11.32
OVCAR-4	23.01
OVCAR-5	53.24
OVCAR-8	21.60
NCI/ADR-RES	77.27
SK-OV-3	60.50
Renal Cancer	
786-0	49.03
A498	-0.50
ACHN	32.29
CAKI-1	42.75
RXF 393	31.60
SN12C	19.65
TK-10	89.84
UO-31	42.56
Prostate Cancer	
PC-3	21.43
DU-145	42.03
Breast Cancer	
MCF7	0.72
MDA-MB-231/ATCC	76.85
HS 578T	36.76
BT-549	14.04
T-47D	10.50
MDA-MB-468	-38.86
Mean	29.20
Delta	68.06
Range	128.70

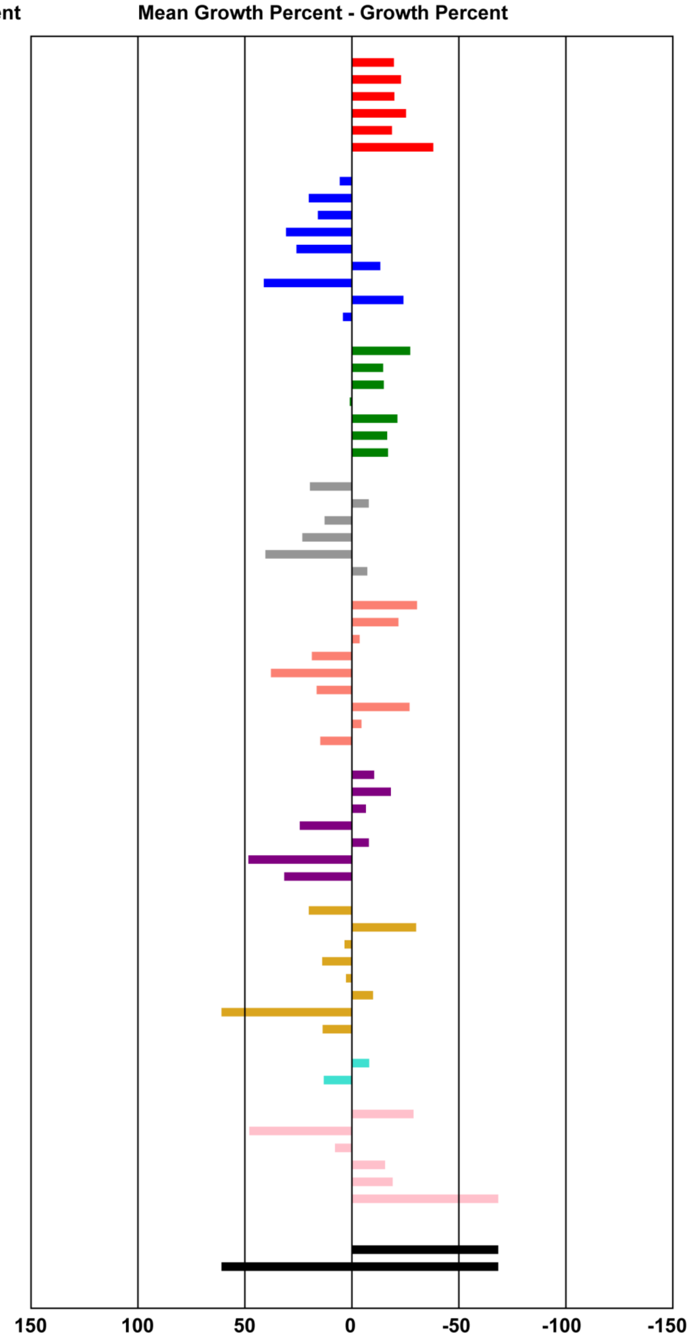

Figure S43: One-dose growth % and mean graph of compound **4c**

Panel/Cell Line	Growth Percent
**Leukemia**	
CCRF-CEM	21.37
HL-60(TB)	37.38
K-562	38.79
MOLT-4	23.55
RPMI-8226	67.45
SR	20.68
**Non-Small Cell Lung Cancer**	
A549/ATCC	17.84
EKVX	73.21
HOP-62	60.52
HOP-92	84.56
NCI-H226	93.14
NCI-H23	61.38
NCI-H322M	77.55
NCI-H460	36.23
NCI-H522	96.13
**Colon Cancer**	
COLO 205	41.29
HCC-2998	79.02
HCT-116	48.65
HCT-15	66.81
HT29	4.33
KM12	64.89
SW-620	46.29
**CNS Cancer**	
SF-268	78.20
SF-295	31.28
SF-539	87.96
SNB-19	60.41
SNB-75	97.23
U251	1.62
**Melanoma**	
LOX IMVI	34.04
MALME-3M	11.78
M14	60.25
MDA-MB-435	93.88
SK-MEL-2	83.28
SK-MEL-28	71.90
SK-MEL-5	88.98
UACC-257	100.34
UACC-62	88.15
**Ovarian Cancer**	
IGROV1	40.17
OVCAR-3	70.66
OVCAR-4	36.40
OVCAR-5	89.16
OVCAR-8	51.27
NCI/ADR-RES	77.02
SK-OV-3	98.26
**Renal Cancer**	
786-0	12.17
A498	72.89
ACHN	35.67
CAKI-1	84.94
RXF 393	77.37
SN12C	41.19
TK-10	94.37
UO-31	75.39
**Prostate Cancer**	
PC-3	46.79
DU-145	85.38
**Breast Cancer**	
MCF7	17.44
MDA-MB-231/ATCC	63.94
HS 578T	69.98
BT-549	106.15
T-47D	59.37
MDA-MB-468	76.13
Mean	60.54
Delta	58.92
Range	104.53

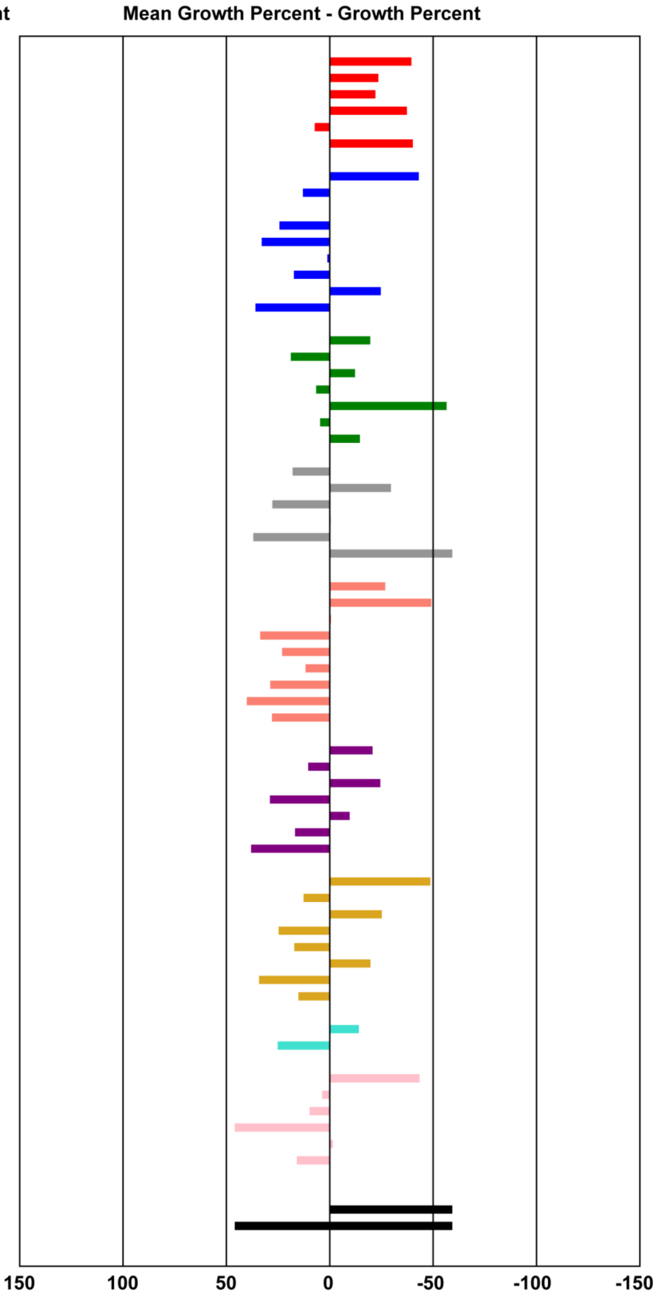

Figure S44: One-dose growth % and mean graph of compound **4d**

Panel/Cell Line	Growth Percent
Leukemia	
CCRF-CEM	15.51
HL-60(TB)	5.77
K-562	8.67
MOLT-4	4.45
RPMI-8226	2.83
SR	-24.18
Non-Small Cell Lung Cancer	
A549/ATCC	-26.66
EKVX	8.23
HOP-62	8.05
HOP-92	28.13
NCI-H226	99.33
NCI-H23	-0.37
NCI-H322M	27.69
NCI-H460	-33.56
NCI-H522	63.24
Colon Cancer	
COLO 205	-23.92
HCC-2998	3.69
HCT-116	-1.88
HCT-15	4.69
HT29	1.67
KM12	-29.12
SW-620	-28.29
CNS Cancer	
SF-268	37.97
SF-295	-49.27
SF-539	37.10
SNB-19	4.35
SNB-75	98.40
U251	-15.25
Melanoma	
LOX IMVI	-55.86
MALME-3M	-58.65
M14	19.67
MDA-MB-435	38.81
SK-MEL-2	38.91
SK-MEL-28	-9.23
SK-MEL-5	36.16
UACC-257	31.74
UACC-62	16.85
Ovarian Cancer	
IGROV1	-27.35
OVCAR-3	-52.88
OVCAR-4	-17.88
OVCAR-5	23.72
OVCAR-8	14.23
NCI/ADR-RES	41.22
SK-OV-3	49.82
Renal Cancer	
786-0	-42.12
A498	56.15
ACHN	-20.18
CAKI-1	61.23
RXF 393	18.92
SN12C	-36.90
TK-10	-4.33
UO-31	21.58
Prostate Cancer	
PC-3	-24.90
DU-145	30.54
Breast Cancer	
MCF7	-19.97
MDA-MB-231/ATCC	9.48
HS 578T	32.95
BT-549	113.46
T-47D	-13.10
MDA-MB-468	-44.13
Mean	7.59
Delta	66.24
Range	172.11

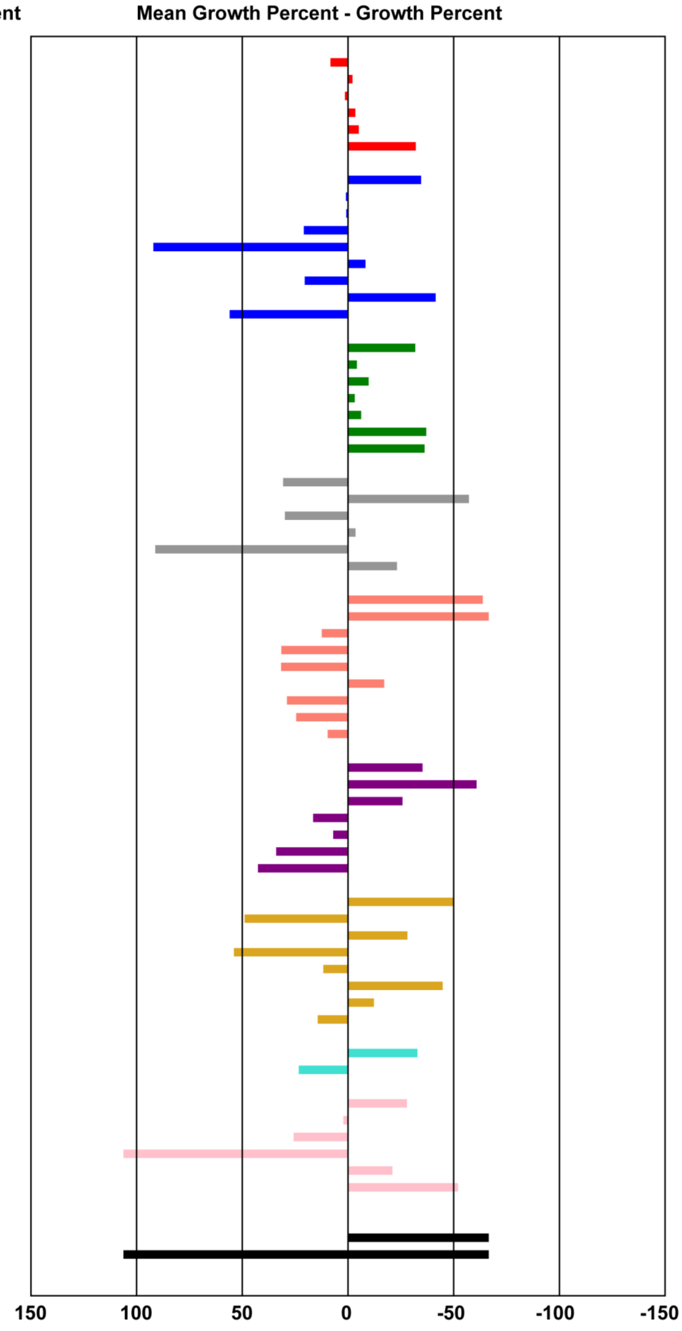

Figure S45: One-dose growth % and mean graph of compound **4e**

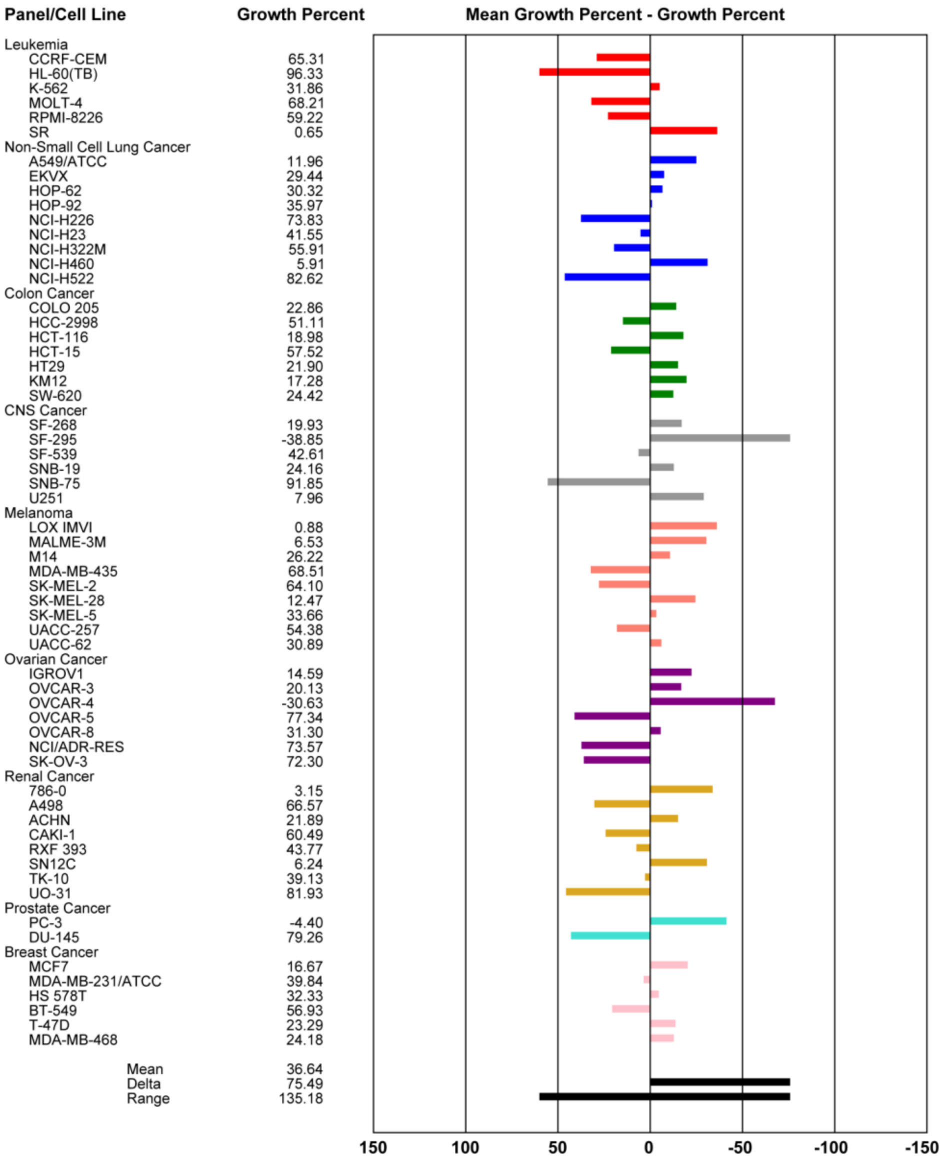

Figure S46: One-dose growth % and mean graph of compound **4g.**

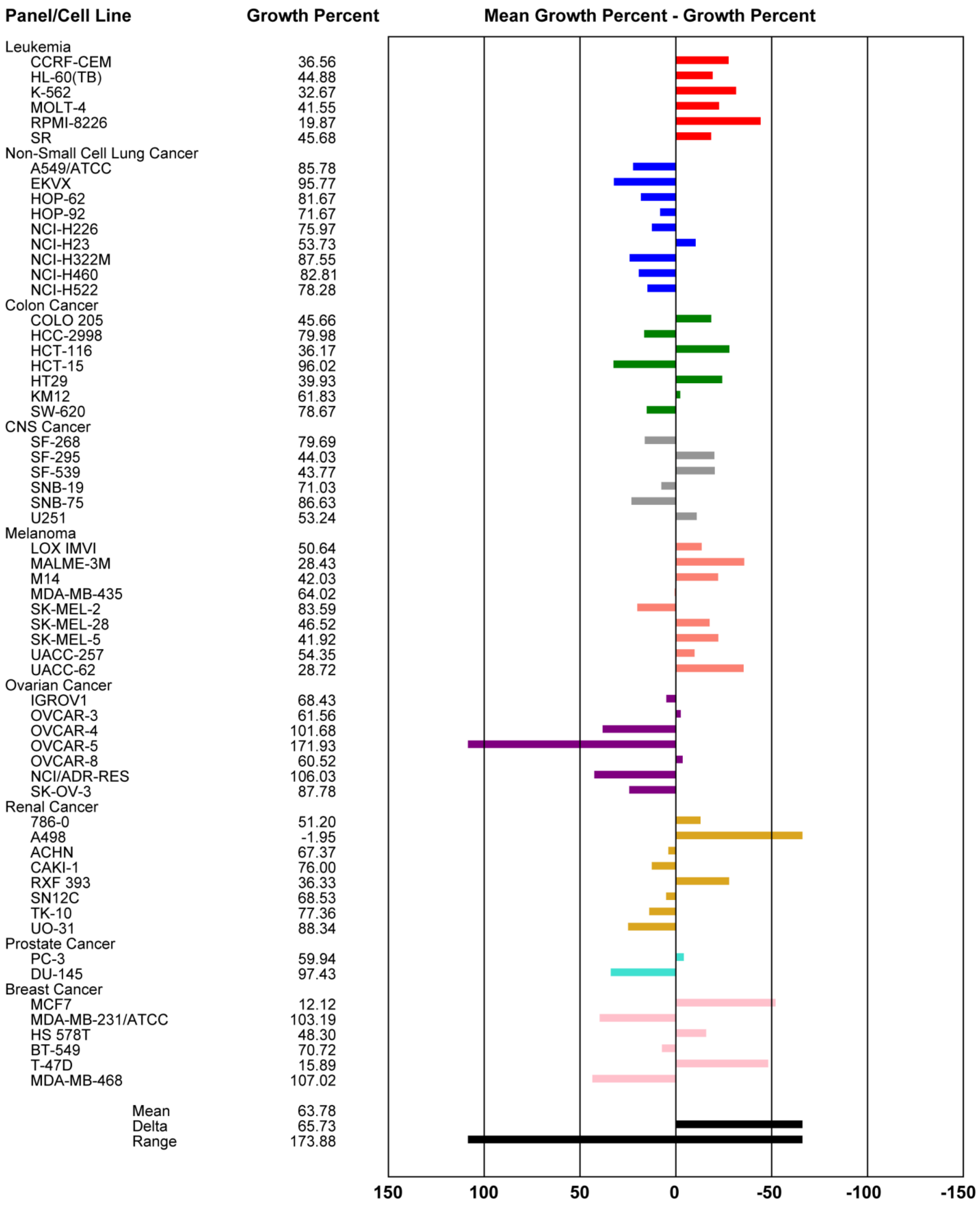

Figure S47: One-dose growth % and mean graph of compound **4h**

Appendix A

4. Experimental

4.1. Chemistry

General details:

Chemicals and solvents were purchased from the Aldrich Chemical Company (Milwaukee, WI), Cornell Lab and El Nasr pharmaceutical chemicals companies, Cairo, Egypt. The progress of the reaction was monitored by TLC sheets precoated with UV fluorescent silica gel MERCK 60 F_{254} that were visualized by U.V. lamp. Melting points were determined on Gallenkamp melting point apparatus and were uncorrected. IR spectra were determined as KBr discs on Shimadzu FT-IR 8400S spectrophotometer and expressed in wave number (υ_{max}) cm^{-1} at Faculty of Pharmacy, Nahda University, Beni-Suef, Egypt. ^{1}H NMR spectra were carried out on Burker 400 MHz spectrophotometer, at Nuclear Magnetic Resonance Center, Faculty of Pharmacy, Beni Suef University and Mansoura University, Egypt, using TMS as internal standard and DMSO-d_6 as a solvent. Chemical shifts were recorded in ppm on δ scale and coupling constants (*J*) in Hertz (HZ). Signals are designed as follows: s, singlet; d, doublet; t, triplet; q, quartet; m, multiplet; brs, broad singlet. ^{13}C NMR spectra were carried out on Burker at 100 MHz spectrophotometer, at Nuclear Magnetic Resonance Center, Faculty of Pharmacy, Beni Suef University and Mansoura University, Egypt, using TMS as internal standard and chemical shifts were recorded in ppm on δ scale. Samples were dissolved in DMSO-d_6. At the Regional Center for Mycology and Biotechnology has carried out the elemental analysis, Al-Azhar University, Cairo, Egypt.

4.2. Biological evaluation

4.2.1. EGFR inhibitory assay

Baculoviral expression vectors including pBlueBacHis2B and pFASTBacHTc were used separately to clone 1.6 kb cDNA coding for EGFR cytoplasmic domain (EGFR-CD, amino acids 645–1186). 5′ upstream to the EGFR sequence comprised a sequence that encoded (His)6. Sf-9 cells were infected for 72h for protein expression. The pellets of Sf-9 cells were solubilized in a buffer containing sodium vanadate (100 μM), aprotinin (10 μg/mL), triton (1%), HEPES buffer(50mM), ammonium molybdate (10 μM), benzamidine HCl (16 μg/mL), NaCl (10 mM),leupeptin (10 μg/mL) and pepstatin (10 μg/mL) at 0°C for 20 min at pH 7.4, followed by centrifugation for 20 min. To eliminate the nonspecifically bound material, a Ni-NTA super flow packed column was used to pass through and wash the crude extract supernatant first with 10mM and then with 100 mM imidazole. Histidine-linked proteins were first eluted with 250 and then with 500 mM imidazole subsequent to dialysis against NaCl (50 mM), HEPES (20 mM), glycerol (10%) and 1 μg/mL each of aprotinin, leupeptin and pepstatin for 120 min. The purification was performed either at 4 °C or on ice. To record autophosphorylation level, EGFR kinase assay was carried out on the basis of DELFIA/Time-Resolved Fluorometry. The compounds were first dissolved in DMSO absolute, subsequent to dilution to appropriate concentration using HEPES (25 mM) at pH 7.4. Each compound (10 μL) was incubated with recombinant enzyme (10 μL, 5 ng for EGFR, 1:80 dilution in 100 mM HEPES) for 10 min at 25°C, subsequent to the addition of 5X buffer (10 μL, containing 2 mM MnCl2, 100 μM Na3VO4, 20 mM HEPES and 1 mM DTT) and ATP-MgCl2 (20 μL, containing 0.1 mM ATP and 50 mM MgCl2) and incubation for 1h. The negative and positive controls were included in each plate by the incubation of enzyme either with or without ATP-MgCl2. The liquid was removed after incubation

and the plates were washed thrice using wash buffer. Europium-tagged antiphosphotyrosine antibody (75 µL, 400 ng) was added to each well followed by incubation of 1h and then washing of the plates using buffer. The enhancement solution was added to each well and the signal was recorded at excitation and emission wavelengths of 340 at 615 nm. The autophosphorylation percentage inhibition by compounds was calculated using the following equation:

$$100\% - [(negative\ control)/(positive\ control) - (negative\ control)]$$

Using the curves of percentage inhibition of eight concentrations of each compound, IC50 was calculated. Majority of signals detected by antiphosphotyrosine antibody were from EGFR because the enzyme preparation contained low impurities.

4.2.2. BRAF^{V600E} inhibitory assay

V^{600E} mutant BRAF kinase assay was performed to investigate the activity of tested compounds against BRAF. Mouse full-length GST-tagged BRAF^{V600E} (7.5 ng, Invitrogen, PV3849) was pre-incubated with drug (1 µL) and assay dilution buffer (4 µL) for 60 min at 25°C. In assay dilution buffer, a solution (5 µL) containing $MgCl_2$ (30 mM), ATP (200 µM), recombinant human full length (200 ng) and *N*-terminal His-tagged MEK1 (Invitrogen) was added to start the assay, subsequent to incubation for 25 min at 25°C. The assay was stopped using 5X protein denaturing buffer (LDS) solution (5 µL). To further denature the protein, heat (70° C) was applied for 5 min. 4-12% precast NuPage gel plates (Invitrogen) were used to carry out electrophoresis (at 200 V). 10 µL of each reaction was loaded into the precast plates and electrophoresis was allowed to proceed. After completion of electrophoresis, the front part of the precast gel plate (holding hot ATP) was cut and afterwards cast-off. The dried gel was developed using a phosphor screen. A reaction without active enzyme was used as

negative control while that containing no inhibitor served as positive control. To study the effect of compounds on cell-based pERK1/2 activity in cancer cells, commercially available ELISA kits (Invitrogen) were used according to manufacturer's instructions.

4.2.3. Caspase-3 activation assay

Allow all reagents to reach room temperature before use. Gently mix all liquid reagents prior to use. Determine the number of 8-well strips needed for the assay. Insert these in the frame(s) for current use. Add 100 µl of the *Standard Diluent Buffer* to the zero standard wells. Well(s) reserved for chromogen blank should be left empty. Add 100 µl of standards and controls or diluted samples to the appropriate microtiter wells. The sample dilution chosen should be optimized for each experimental system. Tap gently on side of plate to mix. Cover wells with *plate cover* and incubate for 2 hours at room temperature. Thoroughly aspirate or decant solution from wells and discard the liquid, Wash wells 4 times. Pipette 100 µl of *Caspase-3 (Active) Detection Antibod*y solution into each well except the chromogen blank(s). Tap gently on the side of the plate to mix. Cover plate with *plate cover* and incubate for 1 hour at room temperature. Thoroughly aspirate or decant solution from wells and discard the liquid, Wash wells 4 times. Add 100 µl Anti-Rabbit IgG HRP Working Solution to each well except the chromogen blank(s). Prepare the working dilution as described in Preparing IgG HRP. Cover wells with the *plate cover* and incubate for 30 minutes at room temperature. Thoroughly aspirate or decant solution from wells and discard the liquid. Wash wells 4 times. Add 100 µl of *Stabilized Chromogen* to each well. The liquid in the wells will begin to turn blue. Incubate for 30 minutes at room temperature and in the dark. The incubation time for chromogen substrate is often determined by the microtiter plate reader used. Many plate readers have the capacity to record a maximum optical density (O.D.) of 2.0. The O.D. values should be monitored, and the substrate reaction stopped before the O.D. of

the positive wells exceeds the limits of the instrument. The O.D. values at 450 nm can only be read after the *Stop Solution* has been added to each well. If using a reader that records only to 2.0 O.D., stopping the assay after 20 to 25 minutes is suggested. Add 100 µl of *Stop Solution* to each well. Tap side of plate gently to mix. The solution in the wells should change from blue to yellow. Read the absorbance of each well at 450 nm having blanked the plate reader against a chromogen blank composed of 100 µl each of *Stabilized Chromogen* and *Stop Solution*. Read the plate within 2 hours after adding the *Stop Solution*. Use a curve fitting software to generate the standard curve. A four-parameter algorithm provides the best standard curve fit. Read the concentrations for unknown samples and controls from the standard curve. Multiply value(s) obtained for sample(s) by the appropriate dilution factor to correct for the dilution in step 3. Samples producing signals greater than that of the highest standard should be diluted in *Standard Diluent Buffer* and reanalyzed.

4.2.4. Caspase-8 activation assay

Cells were obtained from American Type Culture Collection, cells were grown in RPMI 1640 containing 10% fetal bovine serum at 37°C, stimulated with the compounds to be tested for caspase8, and lysed with Cell Extraction Buffer. This lysate was diluted in Standard Diluent Buffer over the range of the assay and measured for human active caspase-8 content. (*Cells are Plated in a density of 1.2 – 1.8 × 10,000 cells/well in a volume of 100µl complete growth medium + 100 ul of the tested compound per well in a 96-well plate for 24 hours before the enzyme assay for Tubulin*.). The absorbance of each microwell was read on a spectro-photometer at 450 nm. A standard curve is prepared from 7human Caspase-8 standard dilutions and human Caspase-8 concentration determined.

4.2.5. Bax activation assay

Bring all reagents, except the human Bax-α Standard, to room temperature for at least 30 minutes prior to opening. The human Bax-α Standard solution should not be left at room temperature for more than 10 minutes. All standards, controls and samples should be run in duplicate. Refer to the Assay Layout Sheet to determine the number of wells to be used and put any remaining wells with the desiccant back into the pouch and seal the ziploc. Store unused wells at 4 °C. Pipet 100 µL of Assay Buffer into the S0 (0 pg/mL standard) wells. Pipet 100 µL of Standards #1 through #6 into the appropriate wells. Pipet 100 µL of the Samples into the appropriate wells. Tap the plate gently to mix the contents. Seal the plate and incubate at room temperature on a plate shaker for 1 hour at ~500 rpm. Empty the contents of the wells and wash by adding 400 µL of wash solution to every well. Repeat the wash 4 more times for a total of 5 washes. After the final wash, empty or aspirate the wells and firmly tap the plate on a lint free paper towel to remove any remaining wash buffer. Pipet 100 µL of yellow Antibody into each well, except the Blank. Seal the plate and incubate at room temperature on a plate shaker for 1 hour at ~500 rpm. Empty the contents of the wells and wash by adding 400 µL of wash solution to every well. Repeat the wash 4 more times for a total of **5** washes. After the final wash, empty or aspirate the wells and firmly tap the plate on a lint free paper towel to remove any remaining wash buffer. Add 100 µL of blue Conjugate to each well, except the Blank. Seal the plate and incubate at room temperature on a plate shaker for 30 minutes at ~500 rpm. Empty the contents of the wells and wash by adding 400 µL of wash solution to every well. Repeat the wash 4 more times for a total of **5 washes**. After the final wash, empty or aspirate the wells and firmly tap the plate on a lint free paper towel to remove any remaining wash buffer. Pipet 100 µL of Substrate Solution into each well. Incubate for 30 minutes at room temperature on a plate shaker at ~500

rpm. Pipet 100 µL Stop Solution to each well. Blank the plate reader against the Blank wells, read the optical density at 450 nm. Calculate the average net Optical Density (OD) bound for each standard and sample by subtracting the average Blank OD from the average OD for each standard and sample. Using linear graph paper, plot the Average Net OD for each standard versus Bax concentration in each standard. Approximate a straight line through the points. The concentration of Bax in the unknowns can be determined by interpolation.

4.2.6. Bcl-2 inhibition assay

Mix all reagents thoroughly without foaming before use. Wash the microwells twice with approximately 300 µL Wash Buffer per well with thorough aspiration of microwell contents between washes. Take caution not to scratch the surface of the microwells. After the last wash, empty the wells and tap microwell strips on absorbent pad or paper towel to remove excess Wash Buffer. Use the microwell strips immediately after washing or place upside down on a wet absorbent paper for not longer than 15 minutes. Do not allow wells to dry. Add 100 µL of Sample Diluent in duplicate to all standard wells and to the blank wells. Prepare standard (1:2 dilution) in duplicate ranging from 32 ng/mL to 0.5 ng/mL. Add 100 µL of Sample Diluent, in duplicate, to the blank wells. Add 80 µL of Sample Diluent, in duplicate, to the sample wells. Add 20 µL of each Sample, in duplicate, to the designated wells. Add 50 µL of diluted biotin-conjugate to all wells, including the blank wells. Cover with a plate cover and incubate at room temperature, on a microplate shaker at 100 rpm if available, for 2 hours. Remove plate cover and empty the wells. Wash microwell strips 3 times as described in step 2. Add 100 µL of diluted Streptavidin-HRP to all wells, including the blank wells. Cover with a plate cover and incubate at room temperature, on a microplate shaker at 100 rpm if available, for 1 hour. Remove plate cover and empty the wells. Wash microwell strips

3 times as described in step 2. Proceed to the next step. Pipette 100 µl of mixed TMB Substrate Solution to all wells, including the blanks. Incubate the microwell strips at room temperature (18° to 25°C) for about 15 minutes, if available on a rotator set at 100 rpm. Avoid direct exposure to intense light. The point, at which the substrate reaction is stopped, is often determined by the ELISA reader. Many ELISA readers record absorbance only up to 2.0 O.D. Therefore, the color development within individual microwells must be watched by the person running the assay and the substrate reaction stopped before positive wells are no longer properly detectable. Stop the enzyme reaction by quickly pipetting 100 µL of Stop Solution into each well, including the blank wells. It is important that the Stop Solution is spread quickly and uniformly throughout the microwells to completely inactivate the enzyme. Results must be read immediately after the Stop Solution is added or within one hour if the microwell strips are stored at 2 - 8°C in the dark. Read absorbance of each microwell on a spectrophotometer using 450 nm as the primary wavelength.

4.3. Statistical analysis

Computerized Prism 5 program was used to statistically analyzed data using one-way ANOVA test followed by Tukey's as post ANOVA for multiple comparison at $P \leq .05$. Data were presented as mean ± SEM.
